# *Prot-SpaM*: fast alignment-free phylogeny reconstruction based on whole-proteome sequences

**DOI:** 10.1093/gigascience/giy148

**Published:** 2018-12-07

**Authors:** Chris-Andre Leimeister, Jendrik Schellhorn, Svenja Dörrer, Michael Gerth, Christoph Bleidorn, Burkhard Morgenstern

**Affiliations:** 1 University of Göttingen, Department of Bioinformatics, Goldschmidtstr. 1, 37077 Göttingen, Germany; 2 Institute for Integrative Biology, University of Liverpool, Biosciences Building, Crown Street, L69 7ZB Liverpool, UK; 3 University of Göttingen, Department of Animal Evolution and Biodiversity, Untere Karspüle 2, 37073 Göttingen, Germany; 4 Museo Nacional de Ciencias Naturales, Spanish National Research Council (CSIC), 28006 Madrid, Spain; 5 Göttingen Center of Molecular Biosciences (GZMB), Justus-von-Liebig-Weg 11, 37077 Göttingen

**Keywords:** alignment-free, phylogeny, spaced words, micro-alignment, proteome, protein comparison, distance method, Kimura, Wolbachia, amino-acid substitutions

## Abstract

Word-based or ‘alignment-free’ sequence comparison has become an active research area in bioinformatics. While previous word-frequency approaches calculated rough measures of sequence similarity or dissimilarity, some new alignment-free methods are able to accurately estimate phylogenetic distances between genomic sequences. One of these approaches is Filtered Spaced Word Matches. Here, we extend this approach to estimate evolutionary distances between complete or incomplete proteomes; our implementation of this approach is called Prot-SpaM. We compare the performance of Prot-SpaM to other alignment-free methods on simulated sequences and on various groups of eukaryotic and prokaryotic taxa. Prot-SpaM can be used to calculate high-quality phylogenetic trees for dozens of whole-proteome sequences in a matter of seconds or minutes and often outperforms other alignment-free approaches. The source code of our software is available through Github: https://github.com/jschellh/ProtSpaM.

## Introduction

Evolutionary relationships between species are usually inferred by comparing homologous gene or protein sequences to each other. Here, groups of orthologous sequences have to be identified first, for which then multiple alignments are to be calculated. There are generally two different strategies of resolving phylogenies based on multiple alignments. In the so-called supermatrix approach, multiple sequence alignments of single genes or proteins are concatenated. A phylogenetic tree is inferred from the resulting matrix, *e.g*., using maximum likelihood [1] or Bayesian inference [2]. Alternatively, gene or protein trees are inferred for every single multiple sequence alignment, and the resulting phylogeny is inferred using coalescent models [3] or supertree [4] approaches.

All of these steps are time consuming, and manual intervention is often required. Therefore, word-based or alignment-free alternatives have been proposed recently, which are much faster and require much less data preparation. Most alignment-free methods compare the word composition of sequences [5, 6, 7, 8, 9, 10], with some approaches also considering background word frequencies [11, 12, 13, 14]; see [15] for a review of these latter approaches. More recently, the spaced-word composition of sequences has been used for sequence comparison [16, 17, 18, 19]. Other alignment-free methods are based on the so-called matching statistics, i.e., they use the length of maximal common subwords [20, 21]. This has been extended to maximal common subwords with a certain number of mismatches [22, 23, 24, 25]. Alignment-free approaches have been recently reviewed in detail [26, 27, 28].

Accurate alignment-free tools are urgently needed because of the huge volume of data generated by new sequencing techniques. Another advantage of alignment-free methods, compared to alignment-based approaches, is the fact that they can be applied to incomplete data, e.g., to unassembled sequencing reads or to partially sequenced genomes [29]. Note that some of the so-called alignment-free approaches are based on comparing words of the input sequences to each other. So, strictly speaking, they are not alignment-free since they align these words to each other. The term "alignment-free" is used nevertheless by most researchers, since these word-based approaches circumvent the need to calculate full pairwise or multiple alignments of the sequences under study.

The above-mentioned approaches to alignment-free sequence comparison calculate *ad hoc* measures of sequence similarity or dissimilarity. They are not based on stochastic models of molecular evolution, and they do not try to estimate distances between sequences in a statistically rigorous way. More recently, some alignment-free approaches have been proposed that are based on explicit models of DNA evolution. These methods are able to estimate the number of substitutions per site that have happened since two nucleic-acid sequences have evolved from their last common ancestor [30, 31, 32, 33, 34, 35].

A main application of alignment-free approaches is comparison of whole genomes. Consequently, most alignment-free methods have been designed to work on DNA sequences. If distantly related species are studied though, phylogenetic trees are usually inferred from protein sequences rather than from DNA sequences. The reason for this is that protein sequences are more conserved than DNA sequences, as synonymous substitutions are not visible in proteins. Thus, for distal species, it may be hard to detect similarities between genes at the DNA sequence level, while homologies may still be detectable among protein sequences. It is therefore highly desirable to have accurate alignment-free software tools that work on protein sequences, in addition to the available tools for DNA sequence comparison. Generic word-frequency methods can be applied to both DNA and protein sequences; the program Feature Frequency Profile (FFP), e.g., has been used for whole-proteome comparison [36]. As mentioned above, however, these methods do not estimate phylogenetic distances in a rigorous way. To date, there are no alignment-free approaches available that can accurately estimate evolutionary distances between protein sequences.

Here, we propose an alignment-free method that estimates the phylogenetic distance between two taxa based on the average number of amino-acid substitutions in the whole proteoms since they evolved from their last common ancestor. Our approach is based on filtered spaced word matches (FSWM), a concept we introduced recently for whole-genome sequence comparison [33]; see [31, 35] for related approaches. We call the implementation of this new approach proteome-based spaced-word matches (Prot-SpaM). The basic idea is to use gap-free pairwise alignments of fixed-length words with matching amino-acid residues at certain pre-defined positions. Such spaced-word matches can be rapidly identified and, after discarding random background matches, the remaining "homologous" spaced-word matches can be used to estimate the phylogenetic distance between two taxa. To our knowledge, this is the first approach that accurately estimates evolutionary distances between protein sequences without the need to calculate full sequence alignments.

To evaluate our approach, we used simulated protein sequences and real-world whole proteomes. Test runs on the simulated sequences show that our distance estimates are very close to the true distances for distance values of up to around 2.0 substitutions per sequence position. On the real-world sequences, we evaluated our approach indirectly by phylogenetic analysis, as is common practice in the field. We used Prot-SpaM to estimate pairwise distances for various sets of taxa, and we applied the neighbor-joining algorithm [37] to calculate phylogenetic trees from the resulting distance matrices. These trees were finally evaluated by comparing them to reference trees that were determined by standard methods and can be considered to be reliable. We show that the trees obtained with our approach are often of high quality, and they are generally more similar to the respective reference trees than trees generated with other alignment-free approaches.

## Method

We consider sequences over an alphabet ${\cal A}$. Here, ${\cal A}$ consists of 20 characters representing the 20 different amino acids. Prot-SpaM is based on so-called spaced-word matches between sequences. For a wildcard character ‘*’ with $*\not\in {\cal A}$ and a binary pattern *P* of length ℓ – , i.e., for a length-ℓ word *P* over {0, 1} –, a spaced-word with respect to *P* is a length-ℓ word *W* over the alphabet ${\cal A} \cup \lbrace *\rbrace$ such that *W*(*i*) = * if and only if *P*(*i*) = 0. An index *i* ∈ {1, …, ℓ} is called a match position of *P* or *W*, respectively, if *P*(*i*) = 1, and a don’t care position otherwise. The number of match positions in a pattern or spaced-word is called its weight *w*. We say that a spaced word *W* with respect to *P* occurs in a sequence *S* at some position *i* if one has *W*(*k*) = *S*(*i* + *k* − 1) for all *k* ∈ {1, …, ℓ} with *P*(*k*) = 1 – ,i.e., for all match positions of *P*.

Moreover, we say that there is a spaced-word match w.r.t. *P* between two sequences *S*_1_ and *S*_2_ at (*i*_1_, *i*_2_) if the same spaced word w.r.t *P* occurs at position *i*_1_ in *S*_1_ and at position *i*_2_ at *S*_2_. In other words, there is a spaced-word match between *S*_1_ and *S*_2_ at (*i*_1_, *i*_2_) if and only if one has *S*_1_(*i*_1_ + *k* − 1) = *S*_2_(*i*_2_ + *k* − 1) for all match positions *k* of *P*. Below is an example for a spaced-word match between two sequences *S*_1_ and *S*_2_ at (2,3) with respect to the pattern *P* = 1100101; the spaced word TN**D*P occurs at position 2 in *S*_1_ and at position 3 in *S*_2_:

**Figure:**
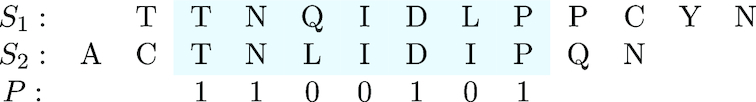


Similar to our original FSWM approach, we estimate distances between protein sequences based on selected spaced-word matches between them, with respect to one or several pre-defined patterns. Distance values are obtained by comparing the amino-acid residues that are aligned to each other at the don’t-care positions of the selected spaced-word matches. This is similar to estimating distances in standard alignment-based approaches; the only difference from those standard approaches is that we are using don’t-care positions of spaced-word matches instead of full sequence alignments.

**Figure 1:**
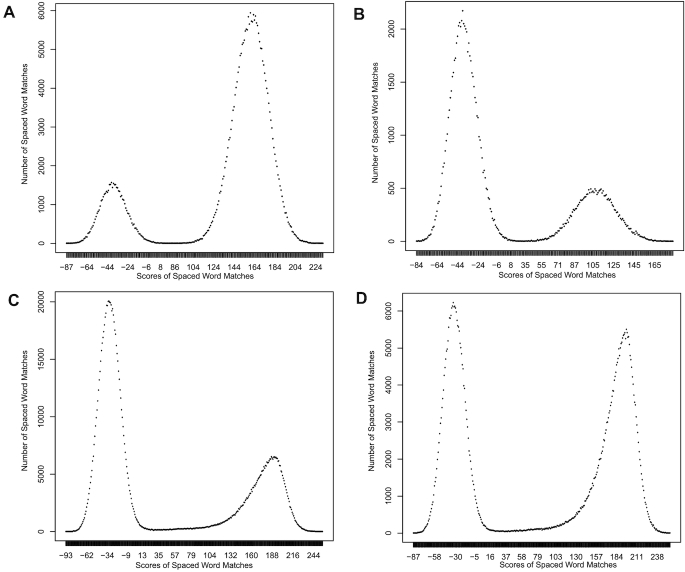
Spaced-word histograms (spamograms) for different datasets. **(A)** and **(B)** are based on simulated insertion and deletion (indel)-free protein sequences with a total length of of 1.6 × 10^6^ amino-acid residues each and with 0.3 **(A)** and 0.75 **(B)** substitutions per position, respectively. **(C)** and **(D)** are from a whole-proteome comparisons of plants, **(C)** comparing *Eucalyptus grandis* with *Capsella rubella* and b comparing *Gossypium raimondii* with *Carica papaya*.

To estimate distances in this way, one has to make sure that only those spaced-word matches are selected that represent homologies, i.e., that the involved spaced-word occurrences go back to the same origin in the last common ancestor of the two proteins that are compared. To distinguish such "homologous" spaced-word matches from random background matches, we calculate a score for each spaced-word match using the BLOSUM62 substitution matrix [38]. Similar to the previous version of our program for nucleic-acid sequences, we define the score of a spaced-word match as the sum of substitution scores of the aligned amino acids at the don’t-care positions. Based on this score, our algorithm decides if a spaced-word match is homologous or not. If its score is below a certain threshold *T*, then a spaced-word match is considered a random match and is not further considered. As a default, we use a threshold value of *T* = 0. To see that this threshold accurately separates homologous from background spaced-word matches, one can plot the number of spaced-word matches with a score *s* against *s*, see Fig. 1. We call such a plot a spaced-word*-*match histogram, or spamogram for short. In these plots, two peaks are typically visible, a peak on the right-hand side representing homologous spaced-word matches and a peak on the left-hand side representing background matches. By default, we are using patterns with a weight of *w* = 6 and with 40 don’t-care positions, i.e., with a length of ℓ = 46.

Moreover, we use a one-to-one mapping of spaced-word occurrences. Note that if sequences *S*_1_ and *S*_2_ are compared and a spaced word *W* occurs *n* time in *S*_1_ and *n*′ times in *S*_2_, then this gives rise to a total of *n* × *n*^′^ spaced-word matches. Taking all of these spaced-word matches into account for phylogeny reconstruction would overemphasize repeated regions where the same spaced words occur multiple times. Instead of using all possible spaced-word matches, we therefore use a one-to-one mapping of spaced-word occurrences in the compared sequences. That is, we ensure that each spaced word occurrence is involved in at least one of the selected spaced-word matches. Formally, if there are two spaced word matches at (*i*_1_, *i*_2_) and at (*j*_1_, *j*_2_), respectively, then we can include both of them simultaneously in our list of selected spaced-word matches only if $i_1 \not= j_1$ and $i_2 \not= j_2$ hold. To achieve this, we use the same "greedy" algorithm that we described previously [33]. For a given spaced word *W*, we calculate the scores of all spaced-word matches involving *W*. We then select them one-by-one in descending order of their scores, always ensuring that each occurrence of *W* is used in at least one of the selected spaced-word matches.

Finally, in order to estimate pairwise distances between two input sequences, we consider the pairs of amino acids aligned to each other at the don’t-care positions of the selected spaced-word matches. Here, we are using the Kimura model [39] that approximates the PAM distance [40] between sequences based on the number of mismatches per position. We are using these two different models since the Kimura model is commonly used to infer distances from the number of mismatches per position in alignments. The BLOSUM matrices, on the other hand, are standard in homology searching. Generally, our procedure to filter out background spaced-word matches is rather robust since the homologous and background regions in our spamograms can be easily distinguished, as can be seen, e.g., in Fig. 1. So, the choice of the substitution matrix to distinguish homologous from background spaced-word matches does not affect the results of our approach very much.

The accuracy and statistical stability of the described approach depends on the number of selected spaced-word matches; the more matches we obtain, the more accurate and stable the results of our method will be. To increase the number of spaced-word matches, the default version of our program uses multiple patterns instead of one single pattern *P*. More precisely, we are using a set ${\cal P} = \lbrace P_1,\dots ,P_m\rbrace$ of *m* binary patterns such that all patterns in ${\cal P}$ have the same length ℓ and the same weight *w* but have their *match* and don’t-care positions arranged differently. We then use spaced-word matches with respect to all patterns $P_i\in {\cal P}$. By default, our program uses sets of *m* = 5 patterns. To find suitable pattern sets, we integrated the tool rasbhari [41] into our implementation. rasbhari uses a "hill climbing" algorithm to optimize pattern sets according to a user-defined criterion. In our program, we use rasbhari to minimize the overlap complexity [42] of pattern sets. Note that rasbhari uses a probabilistic algorithm. It is therefore possible that different program runs of rasbhari return different pattern sets, even if the same parameter values are used. Consequently, different runs of Prot-SpaM on the same sequences and with the same parameter setting can produce slightly different distance estimates.

## Results

To assess the quality of our new approach and to compare it to other alignment-free methods, we used artificially generated as well as real-world protein sequences. For the test runs, we used the default parameters of our program, namely, 6 match positions and 40 don’t care positions, i.e., a total pattern length of 46 –, a threshold of *T* = 0 to discard background spaced-word matches, and sets of *m* = 5 patterns. We compared our program to the following four other alignment-free methods that can be run on protein sequences: Average Common Substring Approach (ACS) [21], FFP [36, 8], kmacs [22], and CVTree [11]. Here, we used version 3.19 of FFP, the other programs that we evaluated did not have version numbers at the time of writing. Since the original implementation of ACS is not publicly available, we used our own implementation of this approach by running kmacs with *k* = 0. The competing tools were used with their default parameters. In addition to evaluating these tools on protein sequences, we ran filtered spaced word matches on the complete genome sequences of the same taxa. All test runs were done on a 10 x *I*ntel(R) Xeon(R) central processing using E7-4850 with 2.00 GHz with four cores each, equaling 40 cores and 1,000 GB of random access memory.

### Distance estimation on simulated sequences

**Figure 2:**
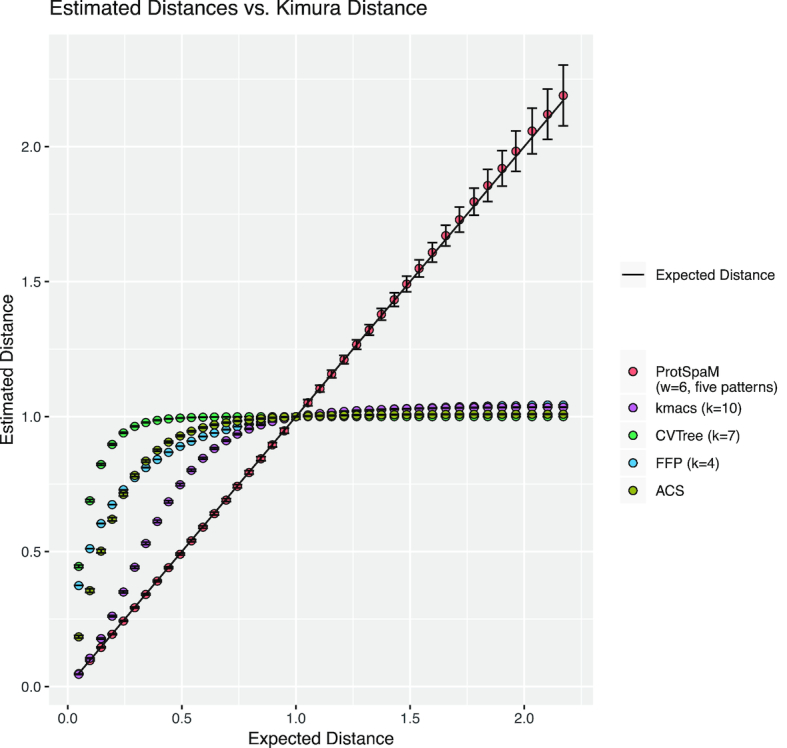
Distances calculated by Prot-SpaM and four other alignment-free methods calculated for pairs of simulated protein sequences plotted against their distances calculated with the Kimura model. Error bars denote standard deviations. Note that Prot-SpaM estimates phylogenetic distances in terms of substitutions that have happened since two sequences evolved from their last common ancestor. The programs kmacs,CVTree,FFP, and ACS, by contrast, do not estimate distances in a rigorous way but rather use *ad hoc* measures of sequence dissimilarity that are not linear functions of the real distances. Also, the absolute values of these distance measures are rather arbitrary for these four other programs. We therefore normalized the distances calculated by kmacs, CVTree, FFP, and ACS such that they have a value of one for sequence pairs with a *Kimura* distance of one.

To evaluate the distances estimated by our program, we simulated sequences with the tool pyvolve [43]. Pyvolve simulates sequences along an evolutionary tree using continuous-time Markov models. It can use various substitution models such as JTT [44] and other models. Since there are no reliable stochastic models for insertions and deletions (indels) in protein sequences, the program produces indel-free sequences. We simulated pairs of sequences of length 100,000 with distances between 0 and 2 substitutions per position in steps of 0.05 using the JTT model. To evaluate the estimated distance values, we generated 1,000 sequence pairs for each distance value and plotted the average of the estimated distances against the real Kimura distance of the respective sequence pairs, calculated with the program protdist from the PHYLIP package [45]. To study the robustness of the estimated distances, we added error bars representing standard deviations to the plot. In addition to running Prot-SpaM with default parameters, i.e., with sets ${\cal P}$ of *m* = 5 patterns , we did a second series of test runs with *m* = 1, i.e., with single patterns. Figure 2 shows the results of these test runs.

### Phylogenetic tree reconstruction

Next, we applied the above alignment-free methods to calculate phylogenetic trees from real-world protein sequences. For four different groups of species, we downloaded all available protein sequences from GenBank [46], in addition we used two data sets from Wolbachia [55], see Table 1 for details. Within each group, we calculated all pairwise distances between the species. We used the distance matrices obtained in this way as input for neighbor-joining [37] and compared the resulting trees to reference trees that we assume reflect the respective correct phylogeny for each group. The Robinson-Foulds (RF) distances [47] between the reconstructed trees and the respective reference trees are shown in Table 2.

**Table 1:** Datasets used in this study to evaluate alignment-free methods, with number of taxa, total size, and source of the reference tree

Taxa	# taxa	Total size (MB)	Source
*Escherichia coli/Shigella*	29	56.41	Zhou et al. [48]
*Wolbachia I*, 252 proteins	19	1.15	Gerth et al. [49]
*Wolbachia I*, whole proteomes	19	7.96	Gerth et al. [49]
*Wolbachia II*	47	14.78	See Supplementary Material
Plants	11	245.05	Hatje and Kollmar [50]
Prokaryotes	813	784.86	Lang et al. [51]
Metazoa	36	585.0	Borowiec et al. [52]

**Table 2:** Robinson-Foulds (RF) distances and relative*RF* distances between trees generated with alignment-free methods and the respective reference trees for various sets of taxa.

Taxa	Prot-SpaM	FSWM	CVTree	FFP, *k* = 4	kmacs, *k* = 10	ACS
	RF distances					
*Escherichia coli/Shigella*	4.00	6	24	40	42	38
*Wolbachia I*, 252 proteins	7.68	8	6	4	8	4
*Wolbachia I*, whole proteomes	6.00	6	8	16	8	12
*Wolbachia II*	19.62	20	44	54	26	16
Plants	0.82	0	6	8	2	6
Prokaryotes	1,020	1,348	886	1,452	880	960
Metazoa	27.1	-	40	62	30	36
	Relative RF distances					
*E. coli/Shigella*	0.08	0.12	0.46	0.77	0.81	0.73
*Wolbachia I*, 252 proteins	0.24	0.25	0.19	0.13	0.25	0.13
*Wolbachia I*, whole proteomes	0.19	0.19	0.25	0.50	0.25	0.38
*Wolbachia II*	0.23	0.23	0.50	0.61	0.30	0.18
Plants	0.05	0.00	0.37	0.50	0.12	0.37
Prokaryotes	0.63	0.83	0.55	0.90	0.54	0.59
Metazoa	0.41	-	0.61	0.94	0.45	0.55

See the main text for details. Since Prot-SpaM uses a probabilistic algorithm, different program runs may produce slightly different results. Therefore, we performed 100 program runs on each dataset and report the average RF distances, except for the large prokaryote dataset where we did only a single program run. All programs were run on protein sequences or whole proteomes, respectively, except for FSWM, which was run on whole-genome sequences of the same species (or on the gene sequences coding for the 252 selected proteins from *Wolbachia I*). We were unable to run FSWM on the whole genomes of the 31 metazoan species since this dataset was too large. Since the original implementation of ACS is not publicly available, we ran our own implementation, kmacs, with *k* = 0 instead.

As mentioned above, Prot-SpaM uses a probabilistic algorithm to generate pattern sets, so the results of different program runs on a sequence set can differ slightly, even if the same parameter values are used. We therefore performed 100 program runs on each dataset. Table 2 lists the average RF distances for these 100 program runs. An exception was the large prokaryotic dataset where we only performed a single program run. Since absolute RF distances are not easy to interpret, Table 2 also reports the relative RF distances, which are obtained from the absolute RF distances by dividing by the maximum possible RF distance for a given dataset. The maximum possible RF distances for a set of *n* taxa is 2 · *n* − 6 [53]. Program run times for the different approaches are shown in Table 3. Trees were visualized with iTOL [54]. Neighbor-joining trees and RF distances were calculated with the phylip package [45].

**Table 3:** Program run time in seconds for different alignment-free approaches on our benchmark datasets.

Taxa	Prot-SpaM	FSWM	CVTree	FFP, *k* = 4	kmacs, *k* = 10	ACS
*Escherichia coli/Shigella*	55	110	125	10	2,518	193
*Wolbachia II*	19	68	46	9	5,302	135
*Wolbachia I*, 252 proteins	3	5	2	1	36	3
*Wolbachia I*, whole proteomes	11	22	21	2	178	26
Plants	464	1,107,720	365	17	17,693	850
Prokaryotes	5,502	244,139	5,492	1,929	915,635	123,520
Metazoa	1,719	-	1,973	43	151,612	9,512

Prot-SpaM and FSWM were run on 40 threads. The other tools do not support multi-threading; therefore, they were run single threaded.

#### Escherichia coli/Shigella

Our first dataset consists of 29 strains of *Escherichia coli* and *Shigella*. For each strain, we were able to download about 4,000-5,000 protein sequences; the total size of this dataset is around 41 MB. Figure 5 shows the reference tree that we used and the tree obtained with the algorithm described here. The reference tree was published by Zhou et al. [48] and is based on a multiple sequence alignment of 2,034 core genes and a maximum likelihood method. As can be seen in Table 2, our approach produced a tree with a topology almost identical to that of the reference tree. All of the 100 program runs that we performed with Prot-SpaM produced the same tree topology; the RF distance between these trees and the reference tree was 4. The other protein-based alignment-free methods led to phylogenies with RF distances to the reference tree of between 24 and 42, while the genome-based tree obtained with FSWM had an RF distance of 6 to the reference tree. These trees are shown in the Supplementary Material.

#### Wolbachia

As a second test case for benchmarking, we analyzed the phylogeny of *Wolbachia* strains, a group of Alphaproteobacteria that are intracellular endosymbionts of arthropods and nematodes [55]. Within *Wolbachia*, 16 distinct genetic lineages (“supergroups”) are currently distinguished (named by letters A-F and H-Q) that may differ in host specificity and type of symbiosis [56]. We re-analyzed a phylogenomic dataset by [57], thereby focusing on relationships of strains within supergroups (*Wolbachia I*). A tree generated with Prot-SpaM from this dataset is shown Fig. 4.

For a second *Wolbachia* benchmarking dataset, we analyzed relationships between supergroups based on available (draft) genomes see below *(Wolbachia II)*. For within supergroup relationships (*Wolbachia I*), a program run of Prot-SpaM on the whole proteome recovered a tree that is largely congruent in topology and branch lengths in comparison to a phylogenomic supermatrix analysis of 252 single-copy orthologs that excluded genes that showed signs of recombination. A comparison based on RF distances showed that our new method outcompetes other available alignment-free programs (Table 2). Interestingly, when analyzing only the 252 ortholog dataset of [57] instead of whole proteomes, RF distances become bigger and other alignment-free methods perform better (Table 2).

Analyzing relationships between supergroups has been historically regarded as a difficult phylogenetic problem [58, 49]. Analyzing all annotated proteins from available genomes with Prot-SpaM supported the monophyly of all supergroups. Moreover, this analysis found the same *Wolbachia* strains basally branching as recent analyses suggested. Surprisingly, the phylogenomic supermatrix analysis of 252 single-copy orthologs that excluded genes that showed signs of recombination of this dataset recovered a topology that differs from the previous study in not supporting the sister group relationship of supergroups A and B. In contrast, as found in previous analyses, the sister group relationship of supergroups A and B is supported by the Prot-SpaM analysis. The Prot-SpaM analysis also recovered some relationships between supergroups that differ from the topologies of our phylogenomic analysis or expectations from a recently published phylogenomic study [59]. However, it is known that supergroups differ in their base (and amino acid) composition, and it is currently unknown how this may impact alignment-free methods. More sophisticated evolutionary models could alleviate these differences in future studies. Nevertheless, in this test case, Prot-SpaM also outperforms other alignment-free methods when comparing the resulting phylogenetic tree with a phylogenomic analyses based on a concatenated supermatrix (Table 2).

For the *Wolbachia II* dataset, we downloaded (if available) proteomes for all available *Wolbachia* draft and fully assembled genomes (47 in total; see Supplementary Material for details). Proteins for *Wolbachia* strains that were lacking this information in the National Center for Biotechnology Information GenBank were derived from translations using GeneMark version 2.5 [60]. We predicted groups of orthologous genes between these proteomes using Orthofinder version 2.1.2 [61] running under default parameters. Single-copy genes present in all analyzed strains (83 in total) were aligned using MAFFT version 7.271 with the ‘L-INS-i’ algorithm [62] and tested for evidence of recombination using the pairwise homoplasy index [63] with window sizes of 10, 20, 30, and 50. Recombining loci were subsequently removed from the dataset and the remaining loci concatenated using FasConCat version 1.0 [64]. The resulting supermatrix (68 loci, 20,787 amino acid positions) was subject to partitioned maximum likelihood analysis following best model and partition scheme selection in IQ-TREE version 1.6.2 [65, 66, 67].

For the whole-proteome sequences of the dataset *Wolbachia I*, the RF distance to the reference tree was 6 for each of the 100 program runs. By contrast, for the 100 runs on the selected protein sequences of the same set of taxa, the average RF distance was 7.68 and the standard deviation was 0.736. For *Wolbachia II*, the average RF distance was 19.62 and the standard deviation was 0.89.

#### Large-scale microbial phylogeny reconstruction

In 2013, J. Eisen’s group published a paper on the phylogeny of the microbial genomes that were available at the time [51]. As a basis of their study, they selected 24 single-copy marker genes and a non-redundant subset of taxa. To obtain such a subset, they used a greedy algorithm by M. Steel [68], making sure that marker genes from different taxa in the resulting subset had a distance to each other of at least 2 substitutions per 100 positions. This way, they obtained a non-redundant subset of 841 bacterial and archeal genomes from the more than 3,000 microbial genomes that were publicly available. Multiple sequence alignments of the marker genes were calculated with hmmalign [69] and were concatenated to a supermatrix that was used as input for the phylogeny programs RAxML [70] and MrBayes [2]. In addition, the authors used the Bayesian tree-reconciliation program BUCKy [71] to the same set of marker genes. The trees they obtained with these different methods were found to be similar to trees obtained based on 16S RNA genes.

To evaluate Prot-SpaM, we used the 841 microbial genomes from Lang et al. [51] and downloaded all protein sequences from these taxa that were available through GenBank. For 28 of the 841 taxa, we were unable to obtain protein sequences, so we obtained a slightly reduced subset of 813 taxa compared to the taxa used by Lang et al. First, we applied Prot-SpaM to all available protein sequences from these 813 taxa. In addition, we ran Prot-SpaM on the protein sequences encoded by the 24 marker genes from Lang et al. and, finally, we applied our previous approach FSWM  [33] to the 841 genome sequences. The trees that we obtained with our different alignment-free approaches are shown in Fig. 6, together with the maximum likelihood tree from [51], which we considered as a reliable reference. Clades from this reference tree are color coded in Fig. 6. As can be see from the color coding, the tree obtained with Prot-SpaM from the available protein sequences contains essentially the same clades as the reference tree. There are some differences within the clades that should be further investigated (J. Eisen, personal communication). The RF distance between the tree obtained with Prot-SpaM and the reference tree was 1,020.

#### Plants

**Figure 3:**
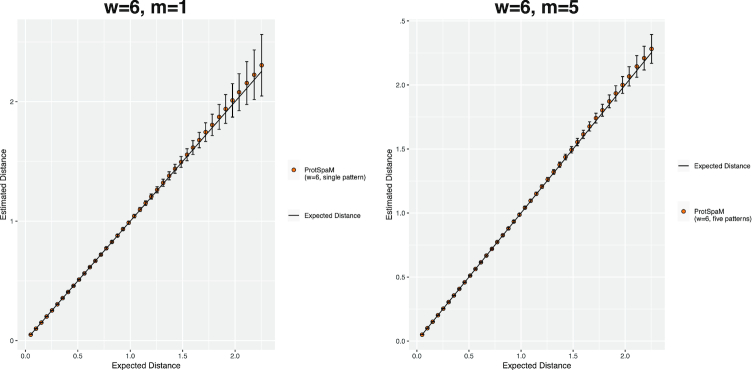
Distances calculated by Prot-SpaM for pairs of simulated protein sequences with a single binary pattern (*m* = 1, left) and with the default multiple-pattern option (*m* = 5, right). We performed 1,000 program runs for each value of *m*. The plot shows the average of the calculated distances; standard deviations are shown as error bars.

Next, we used a set of plant taxa that has been studied by Hatje and Kollmar [50] and that we had used in previous studies to evaluate alignment-free approaches to genome sequence comparison [17, 22, 33]. The dataset that we used in these previous studies consisted of 14 Brassicales species. In GenBank, however, the proteomes could be downloaded for only 11 of the 14 species, so we had to limit our test runs to these 11 species. To obtain a reference tree, we used a tree that has been obtained with multiple sequence alignment and maximum likelihood, as published by Hatje and Kollmar [50; Figure 3 B]. From this tree, we removed the three species for which we could not obtain the proteome sequences in GenBank. Figure 7 shows the reference tree of the 14 original species, together with trees of the 11 species with available proteomes, calculated with the alignment-free methods that we evaluated here. For the 100 program runs with Prot-SpaM, the average RF distance between the resulting trees and the reference tree from [50] was 0.82 and the standard deviation was 0.9.

#### Metazoa

Finally, we used a set of 36 proteomes from 34 metazoan and 2 choanoflagellate taxa. These taxa have been previously used by Borowiec et al. [52] to study the position of the Ctenophora within the phylogenetic tree of the metazoan kingdom. The same set of taxa has also been used in a study by Zhou et al. [72] to evaluate maximum-likelihood programs for phylogeny reconstruction. As a reference tree, we used the tree published in [52]. The average RF distance of the Prot-SpaM trees to this reference tree was 27.1, with a standard deviation of 1.51.

#### Parameter values and number of selected spaced-word matches

*Prot-SpaM*has four major parameters that can be adjusted by the user: the weight *w* (=number of match positions) of the binary patterns and spaced words, their length ℓ, the number *m* of different binary patterns used by the program, and the cutoff value *T* to separate homologous from background spaced-word matches. To see how these parameters influence the results of our software and to find suitable default values, we ran Prot-SpaM with varying values of these four parameters. Here, we modified one parameter at a time, using the respective default values of the remaining three parameters. The results are summarized in Tables 4 and 5. As can be seen in these tables, there are no values for *w*, ℓ, and *T* that work best for all datasets, but our default values seem to be a reasonable compromise. Using sets of *m* = 5 binary patterns does not improve the quality of the produced trees in terms of their RF distances to the reference trees compared to program runs with single patterns. Table 3 shows, however, that the distance values estimated by Prot-SpaM become statistically more stable if multiple patterns are used.

**Figure 4:**
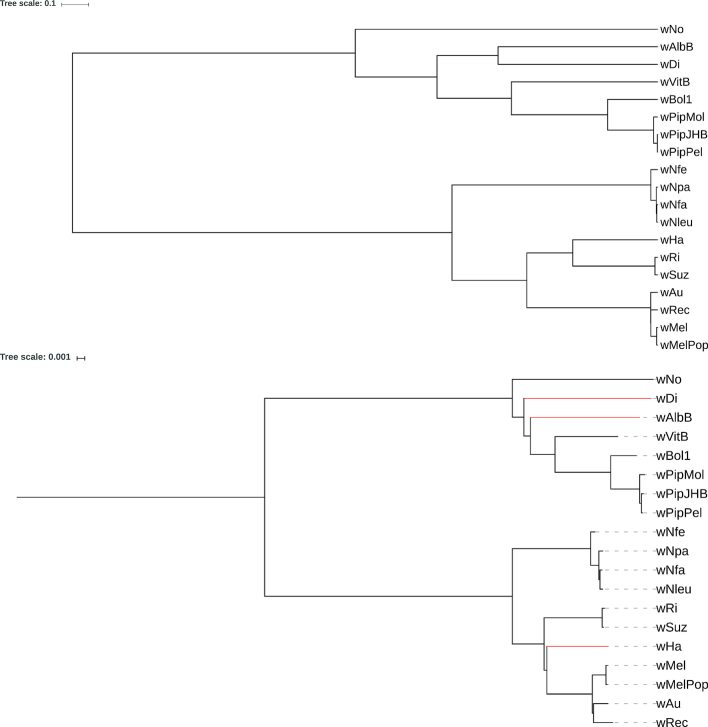
Reference tree for our dataset *Wolbachia I* (top) and tree calculated with Prot-SpaM using whole-proteome sequences of the same taxa (bottom) (see main text for details). Topological differences between the two trees are shown in red in the Prot-SpaM tree.

**Figure 5:**
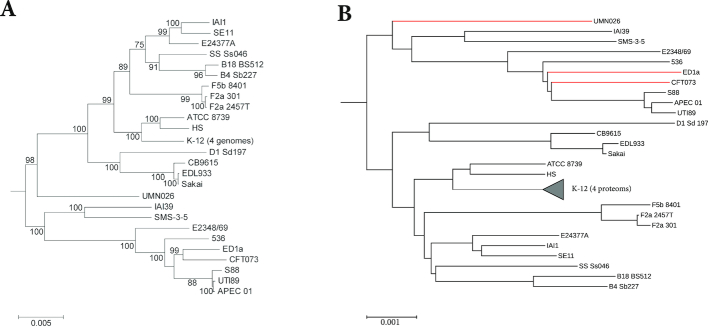
Reference tree **(A)** from [48] and tree calculated with Prot-SpaM with default parameters **(B)** for a set of 29 *Escherichia coli* and *Shigella* strains. Differences in the topologies between the two trees are marked in red.

**Table 4:** Program runtime and RF distances to reference trees for different parameter values with Prot-SpaM for the *E. coli/Shigella* and *Wolbachia* proteomes.

*E. coli/Shigella*								
Weight *w*		**6**	8	10				
Runtime [s]		**55.4**	47.4	46.9				
RF distance		**4**	6.02	6.76				
Length ℓ	36	**46**	56	66				
Runtime [s]	47.5	**55.4**	60.3	66.9				
RF distance	4	**4**	4.02	4.84				
# patterns *m*	1	3	**5**	7				
Runtime [s]	13.5	34.4	**55.4**	75.94				
RF distance	4.12	4.02	**4**	4				
Threshold *T*	-50	-25	**0**	25	50	75	100	125
Runtime [s]	55.2	55.4	**55.4**	55.1	55.3	55.3	55.1	55.2
RF distance	11.92	12	**4**	12	12	12	12	12
*Wolbachia II*								
Weight *w*	4	**6**	8	10				
Runtime [s]	112.4	**19.4**	18	17.8				
RF distance	20.38	**19.68**	22	22				
Length ℓ	36	**46**	56	66				
Runtime [s]	17	**19.4**	21.5	23.9				
RF distance	19.78	**19.68**	19.7	19.78				
# patterns *m*	1	3	**5**	7				
Runtime [s]	5.4	12.5	**19.4**	26.5				
distance	19.06	19.12	**19.68**	19.82				
Threshold *T*	-50	-25	**0**	25	50	75	100	125
Runtime [s]	19.6	19.6	**19.4**	19.4	19.4	19.4	19.4	19.4
RF distance	22.18	18	**19.68**	19.86	20.16	20.7	21.04	22

We ran our program with different values for the weight *w* and length ℓ of the spaced words for different numbers of patterns and for different values of the threshold *T*. Here, we modified the value of one of these parameters at a time and used the default values for the remaining three parameters. Default values of the modified parameters and the resulting runtimes and RF distances are shown in bold font. Since Prot-SpaM uses a probabilistic algorithm to generate pattern sets, we performed 100 program runs for each set of parameters; the table shows the average RF distances of these 100 runs.

**Table 5:** Program runtime and RF distances to reference trees for different parameter values with Prot-SpaM for the plant and metazoan proteomes

Plants								
Weight *w*	4	**6**	8	10				
Runtime [s]	57,578	**464**	320	325				
RF distance	2	**0**	4	6				
Length ℓ	36	**46**	56	66				
Runtime [s]	383	**464**	441	494				
RF distance	2	**0**	0	0				
# patterns *m*	1	3	**5**	7				
Runtime [s]	91	255	**464**	572				
RF distance	0	0	**0**	2				
Threshold *T*	-50	-25	**0**	25	50	75	100	125
Runtime [s]	383	402	**464**	409	391	459	439	430
RF distance	2	2	**0**	0	0	0	2	4
Metazoa								
Weight *w*		**6**	8	10				
Runtime [s]		**1,719**	1,584	1,518				
RF distance		**30**	26	30				
Length ℓ	36	**46**	56	66				
Runtime [s]	1,351	**1,719**	1,584	2,089				
RF distance	26	**30**	24	26				
# patterns	1	3	**5**	7				
Runtime [s]	427	890	**1,719**	2,078				
RF distance	24	28	**30**	26				
Threshold *T*	-50	-25	**0**	25	50	75	100	125
Runtime [s]	2,539	2,337	**1,719**	2,269	2,150	1,906	1,783	1,797
RF distance	30	24	**30**	26	28	28	30	34

Parameter values as in Table 4. Because of the size of these datasets, we performed only one program run per parameter set. We ran our program with different values for the weight *w* and length ℓ of the spaced words for different numbers of patterns and for different values of the threshold *T*. Here, we modified the value of one of these parameters at a time and used the default values for the remaining three parameters. Default values of the modified parameters and the resulting runtimes and RF distances are shown in bold font.

The number of spaced-word matches in a pairwise sequence comparison depends on how similar the two sequences are to each other; see [18] for details. Consequently, the number of spaced-word matches that are selected by our program to estimate phylogenetic distances also depends on the degree of similarity between the compared sequences. We found two extreme cases with our test data, one in the *E. coli/Shigella* dataset where most taxa are closely related to each other and another one in the *Metazoan* dataset that contains taxa with very large evolutionary distances. In the pairwise comparison of *E. coli*O157:H7 strain EDL933 with *E. coli*O157:H7 Sakai (EHEC), Prot-SpaM selected more than 6,000,000 spaced-word matches. These two proteomes have less than 1,600,000 amino acids each, so in this case >3.75 spaced-word matches per sequence position were selected. By contrast, less than 13,000 spaced-word matches were selected in the comparison of *Brugia malayi* and *Homo sapiens*. The latter proteome has a length of more than 75,000,000 amino acids, so here less than 0.00017 spaced-word matches per sequence position were selected.

## Discussion

A number of so-called alignment-free approaches have been proposed in recent years to rapidly calculate phylogenetic distances between genomic sequences. Earlier approaches are based on *k*-mer frequencies or on the length of common substrings. These approaches have been applied not only to DNA but also to protein sequences. A drawback of these methods is that they can only calculate rough measures of sequence similarity or dissimilarity; they do not estimate phylogenetic distances in a rigorous way. More recently, word-based methods have been developed that can accurately estimate phylogenetic distances between genomic sequences based on stochastic models of DNA evolution. One of these approaches is FSWM.

In this study, we introduced Prot-SpaM, a new implementation of FSWM to compare complete or incomplete proteome sequences to each other. To our knowledge, Prot-SpaM is the first tool that can accurately estimate phylogenetic distances between protein sequences without the need to calculate full sequence alignments. Our benchmark results show that distance estimates obtained with our approach are accurate for a large range of phylogenetic distances. Distances calculated with CVTree,ACS,FFP, and kmacs, by comparison, are monotonously increasing with the number of substitutions between the compared sequences. The obtained distance values are far from proportional to the real distances, and they flatten out somewhere between 0.5 and 1.5 substitutions per position (see Fig. 2). By contrast, Prot-SpaM estimates distances with high accuracy for up to around 2.0 substitutions per position. For higher distance values, the calculated distances become less stable, as can be seen from the error bars in Fig. 2. Moreover, for large distances, our program tends to slightly overestimate distances.

In our program evaluation, we used all competing software tools with their respective default parameters, if such default values were recommended by their developers. It should be mentioned, however, that some of the evaluated programs might produce better results with different parameter settings. The program FFP, e.g., uses a default *k*-mer length of *k* = 4, so we used this value in our study. It has been reported, however, that FFP may perform better on protein sequences if larger values of *k* are used [36]. A comprehensive investigation of the effects of different parameters on the software programs evaluated in this study is beyond the scope of this article. Interested readers are encouraged to run these programs with different parameter values to see if their results on our benchmark data can be improved.

Prot-SpaM produced high-quality trees and was superior to other alignment-free methods for the *E.coli/Shigella* and plant datasets, as shown in Table 2. On the *Wolbachia* datasets, it still performed reasonably well and was again superior to competing approaches on the whole-proteome sequences, but it was outperformed by word-frequency methods on the 252 selected orthologous proteins. A possible explanation of this result is discussed below. On the large prokaryote dataset and for the metazoan set, by contrast, none of the compared programs could reproduce the reference trees that we used in our evaluation. These are difficult datasets since they span very large evolutionary distances. Also, it should be mentioned that there are no absolutely reliable reference trees available for these datasets. For the metazoan dataset, e.g., the position of the ctenophores is still a matter of debate [73, 74, 75]. On the metazoans, Prot-SpaM performed better than other alignment-free approaches, while on the large prokaryote dataset, CVTree,ACS, and kmacs were superior.

**Figure 6:**
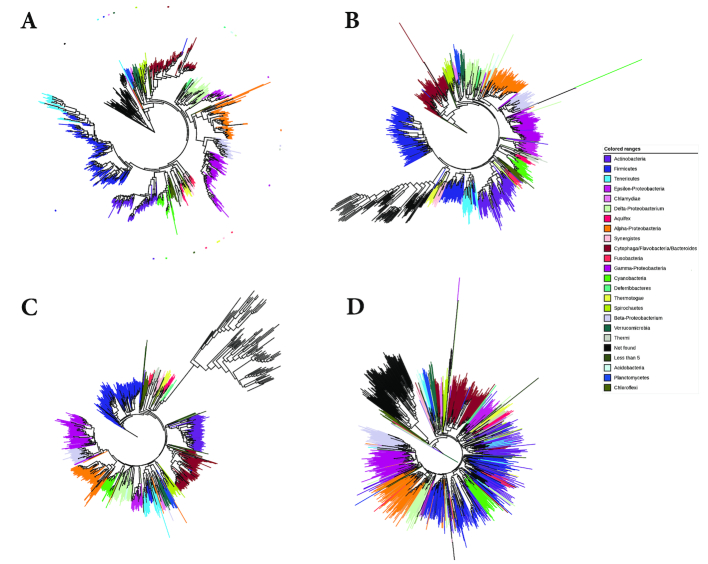
Phylogenetic trees for a large set of microbial taxa studied by Lang et al. [51]. **(A)** Maximum-likelihood tree constructed by Lang et al. based on a super alignment of 24 selected genes. **(B)** Tree constructed with our approach, as described here, for 813 taxa for which the proteomes are available in GenBank. **(C)** Tree constructed with our approach based on the proteins corresponding to the 24 genes selected by Lang et al. **(D)** Tree reconstructed using our program FSWM [33] on the 841 whole-genome sequences.

An interesting result is the performance of Prot-SpaM compared to our previous approach FSWM that takes genomic sequences as input. For most groups of taxa in our study, the results of Prot-SpaM and FSWM were of similar quality in the sense that the RF distances to the reference trees were comparable for both approaches. However, for the set of 813 prokaryote taxa, our new spaced-words approach performed better on whole proteomes than our previous approach on whole genomes, as shown in Fig. 6 and Table 2. This discrepancy is most likely due to the large phylogenetic distances in this dataset. For such distantly related sequences, homologies are generally better detectable at the protein level than at the DNA level.

**Figure 7:**
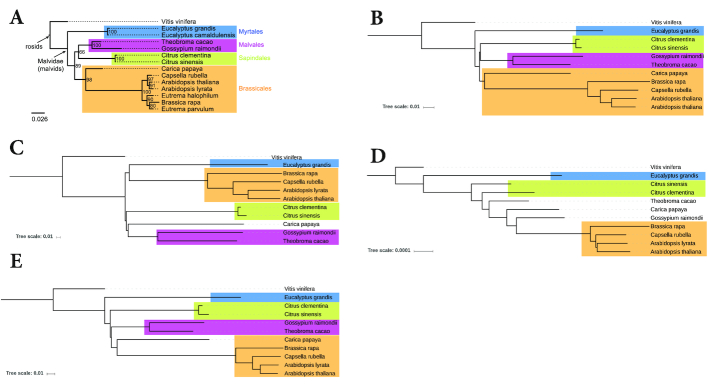
Phylogenetic trees of plant taxa. **(A)** Reference tree from [50] and trees constructed with **(B)** the approach described here and by **(C)** ACS [21], **(D)** FFP [8], and **(E)**kmacs [22]. The original dataset contained 14 taxa, but only for 11 taxa could the proteomes be downloaded through GenBank. For completeness, we show the reference for all 14 taxa.

"Alignment-free" methods for phylogeny reconstruction can be directly applied to whole-genome or whole-proteome sequences, without the need to select orthologous genes or proteins in a first step. This is generally seen as an advantage over more traditional, alignment-based approaches since the task of finding orthologs is time-consuming and often involves manual intervention. On our dataset *Wolbachia I*, we actually obtained better RF distances with Prot-SpaM and FSWM when we applied these programs to the whole-protein or whole-genome sequences, respectively, than when we applied them to the 252 selected orthologous proteins or to the genes coding for those proteins (see Table 2). These results are in contrast to those with the more traditional alignment-free methods FFP, CVTree, and ACS that are based on word frequencies or on the length of common substrings. The latter programs performed better on the selected orthologous proteins of the *Wolbachia I* dataset than on the corresponding whole-proteome sequences.

A possible explanation of this phenomenon is that Prot-SpaM and FSWM can reliably distinguish between homologous and background spaced-word matches and use only homologous matches for phylogenetic inference. With the one-to-one spaced-word matching, they can also reduce the number of paralogous spaced-word matches. Therefore, they can be applied to whole proteomes or whole genomes without being too much confused by paralogs or by non-related parts of the sequences. Here, the benefits of using larger input sequence sets seem to outweigh the disadvantage of including possible non-related sequences, paralogs, or sequences with recombinations. Previously introduced word-frequency or substring-length methods, by contrast, do not distinguish between homologous and non-homologous parts of the sequences. Therefore, these approaches tend to be confused by input sequences that contain paralogs or are only locally related to each other.

Table 3 shows that the run time of Prot-SpaM is superior to that of CVTree and ACS on protein sequences. By far, the fastest alignment-free method on whole proteomes was FFP, the slowest one was kmacs. On the plant proteomes, Prot-SpaM was three orders of magnitude faster than FSWM on the genome sequences of the same species. This is not surprising given the fact that in eukaryotes only a small part of the genome is protein-coding sequence. The total size of the 11 plant genomes was 3.8 GB compared to 245 MB for the corresponding proteome sequences (note that for the genome sequences, both strands are considered and the number of background spaced-word matches scales quadratically with the sequence length).

Prot-SpaM has four major parameters that can be adjusted by the user: the weight *w* and the length ℓ of the patterns and spaced words, respectively, the cutoff value *T* to distinguish homologous from random spaced-word matches, and the number *m* of different patterns used to generate spaced-word matches. We provide default values for these parameters, and Tables 4 and 5 show that reasonable results can be obtained with a rather broad range of parameter values. These tables also show that the quality of the produced trees, as measured by the RF distances to the reference trees, could not be improved by using *m* = 5 patterns compared to the single-pattern option, i.e., *m* = 1. The statistical stability of our distance estimates, however, is increased if multiple patterns are used; therefore, we are using *m* = 5 patterns by default. Since runtime and memory usage of our program increase with the number *m* of pattern, it may be advisable to use the single-pattern option if very large datasets are to be analyzed.

It should be mentioned that traditional approaches to phylogeny reconstruction that are based on multiple sequence alignment are still more accurate than alignment-free approaches that have been proposed in recent years. The main advantage of these novel approaches is their high speed, which makes it possible to apply them to the large sequence datasets that are now available. A program run of Prot-SpaM on whole-proteome sequences of the set *Wolbachia II* that consists of 47 taxa took only 19 seconds. Another advantage of our approach is that it can reliably distinguish between local homologies and random background similarities. It can, thus, be applied to complete or incomplete proteomes, and it is not necessary to select orthologous genes or proteins in a first step. Therefore, we think that Prot-SpaM should be a useful addition to existing approaches to phylogeny reconstruction.

## Availability of source code and requirements

- Project name: Prot-SpaM
- Project home page: https://github.com/jschellh/ProtSpaM
- Operating system(s): linux
- Programming language: C++
- Other requirements: none
- License: GNU GPL
- Any restrictions to use by non-academics: none

## Availability of supporting data

The sequence datasets and trees used for the program evaluation can be downloaded from http://projects.gobics.de/data/protspam/paperData.tgz. Additional supporting data and snapshots of the code are available in the *GigaScience* repository, GigaDB [76]

## Abbreviations

ACS: Average Common Substring Approach; BLOSUM: Blocks Substitution Matrix; CVTree: Composition Vector Tree; FFP: Feature Frequency Profile; FSWM: filtered spaced word matches; indel: insertions and deletions ; kmacs: k-Mismatch Average Common Substring Approach; PHYLIP: PHYLogeny Inference Package; Prot-SpaM: proteome-based spaced-word matches; rasbhari: Rapid Approach for Seed optimization Based on a Hill-climbing Algorithm that is Repeated Iteratively; RF: Robinson-Foulds; spamogram: Spaced-Word-Match Histogram.

## Competing interests

The authors declare that they have no competing interests

## Funding

The project was partially funded by the Volkswagen Foundation, project VWZN3157. We acknowledge support by the Open Access Publication Funds of the Göttingen University.

## Author contributions

C.A.L. designed the study, co-supervised the project, and drafted parts of the manuscript. J.S. implemented the software and did most of the program evaluation. S.D. contributed to the program evaluation. M.G. and C.B. analyzed and discussed the results on *Wolbachia* and wrote parts of the manuscript. B.M. co-supervised the project and wrote most of the manuscript.

## Supplementary Material

GIGA-D-18-00169.pdf

GIGA-D-18-00169_R1.pdf

GIGA-D-18-00169_R2.pdf

Response_to_Reviewer_Comments_Original_Submission.pdf

Response_to_Reviewer_Comments_Revision_1.pdf

Response_to_Reviewer_Comments_Revision_2.pdf

Reviewer_1_Report_(Original_Submission) -- Sharma Thankachan 5/31/2018 Reviewed

Reviewer_3_Report_(Original_Submission) -- Se-Ran Jun 6/18/2018 Reviewed

Reviewer_3_Report_revision_1 -- Se-Ran Jun 9/17/2018 Reviewed

Reviewer_3_Report_Revision_2 -- Se-Ran Jun 9/29/2018 Reviewed

Reviewer_4_Report_(Original_Submission) -- Sriram Chockalingam 6/20/2018 Reviewed

Reviewer_4_Report_Revision_1 -- Sriram Chockalingam 9/18/2018 Reviewed

Reviewer_5_Report_(Original_Submission) -- Alexey Kozlov 6/21/2018 Reviewed

Reviewer_5_Report_Revision_1 -- Alexey Kozlov 9/18/2018 Reviewed

Supplemental File
