# Supplementary material for: Prot-SpaM: fast alignment-free phylogeny reconstruction based on whole-proteome sequences
Source: Gigascience. 2018 Dec 7;8(3):giy148. doi: 10.1093/gigascience/giy148 (PMC6436989; doi:10.1093/gigascience/giy148)
Supplement: GIGA-D-18-00169_R1.pdf [file giy148_giga-d-18-00169_r1.pdf]

GigaScience

Prot-SpaM: Fast alignment-free phylogeny reconstruction based on whole-proteome sequences

--Manuscript Draft--

**Manuscript Number:**	GIGA-D-18-00169R1
**Full Title:**	Prot-SpaM: Fast alignment-free phylogeny reconstruction based on whole-proteome sequences
**Article Type:**	Technical Note
**Funding Information:**	
**Abstract:**	Word-based or ‘alignment-free’ sequence comparison has become an active research area in bioinformatics. While previous word-frequency approaches calculated rough measures of sequence similarity or dis-similarity, some new alignment-free methods are able to accurately estimate phylogenetic distances between genomic sequences. One of these approaches is Filtered Spaced Word Matches. Herein, we ex-tend this approach to estimate evolutionary distances between com-plete or incomplete proteomes; our implementation of this approach is called Prot-SpaM. We compare the performance of Prot-SpaM to other alignment-free methods on simulated sequences and on various groups of eukaryotic and prokaryotic taxa. Prot-SpaM can be used to calculate high-quality phylogenetic trees for dozens of whole-proteome sequences in a matter of seconds or minutes and often outperforms other alignment-free approaches. The source code of our software is available through Github: https://github.com/jschellh/ProtSpaM
**Corresponding Author:**	Chris-Andre Leimeister<br><br>GERMANY
**Corresponding Author Secondary Information:**	
**Corresponding Author's Institution:**	
**Corresponding Author's Secondary Institution:**	
**First Author:**	Chris-Andre Leimeister
**First Author Secondary Information:**	
**Order of Authors:**	Chris-Andre Leimeister
	Jendrik Schellhorn
	Svenja Schöbel
	Michael Gerth
	Christoph Bleidorn
	Burkhard Morgenstern
**Order of Authors Secondary Information:**	
**Response to Reviewers:**	Reviewer #1:<br><br>REVIEWER:<br>All defied terms and parameters should have consistency. There are some terms which appear both in italic font and simple text font, \textit{i.e.} \textit{homologous}, \textit{spaced-word matches} and \textit{don't-care positions}. Also some words in page 3 are underlined.<br><br>REPLY:

Powered by Editorial Manager® and ProduXion Manager® from Aries Systems Corporation

As usual, some words are in italics to emphasize them, so if a word is in italics at some point, it does not need to be in italices in other parts of the manuscript. "i.e." is in italics throughout the paper, as it should be. We partially underlined some words to indicate where the acronym "Prot-SpaM" comes from. We don't insist on this; if that's not in line with the journal style, we will remove the underlining.

REVIEWER:
Cite Kimura model and PAM distance in line 26 of page 5 should be referenced. MegaByte shoulb be written as "MB" not "mb".

REPLY:
Done.

==

Reviewer #3:

Minor Comments:

REVIEWER:
-. References are not ordered.

REPLY:
We used \bibliographystyle{plain}; therefore references are currently in alphabetic order. In case the paper gets accepted, we will of course use the bib style that is used by the publisher.

REVIEWER:
-. On page 4, the cartoon for explaining of concept of spaced-word matches is not correct.

REPLY:
The cartoon is correct. It seems that our previous definitions were not clear enough, and there was a misunderstanding about our concept of "spaced words". We therefore revised the definitions and tried to explain the meaning of "spaced words" and "spaced word matches" more clearly.

REVIEWER:
-. Figure 2 legend says ProtFSWM not Prot-SpaM

REPLY:
Done

REVIEWER:
-. On Page 5, authors checked if spaced-words matches in the two compared sequences are one-to-one mapping. Please describe how one-to-one mapping from one-to-multiple or multiple-to-multiple was defined?

REPLY:
See above. Our definition of "spaced-word matches" was a bit sloppy, this may have led to some misunderstandings. We now give formally more correct definitions of "spaced words" and "spaced-word matches". Also, we revised the paragraph on "one-to-one mapping" and used a more precise definition. We hope, this helps to clarify this point.

REVIEWER:
-. On page 5, authors used Kimura model to approximate PAM distance. Please put a reference for Kimura model. If there are parameters involved in Kimura model, please describe how to estimate those parameters.

REPLY:
Done.

REVIEWER:
-. Authors used BLOSUM62 when to distinguish homologous spaced-word matches with random spaced-word matches. But, authors approximated PAM distance between protein sequences. Please describe rational different substitution matrices used for different purposes?

REPLY:
Thanks for pointing this out. We added a paragraph to clarify this point (end of the "Methods" section).

REVIEWER:
-. For Table 1, Table 2, Table 3, Figure 2, and Figure 5, please describe which length of K-mer was used for FFP method.

REPLY:
We used the default value which is k=4.

REVIEWER:
-. One page 8, "One interpretation is that misleading signal stemming from recombination events between Wolbachia strains is less problematic for alignment-free analysis then a reduction in he dataset size." <= please revise the sentence.

REPLY:
We removed this sentence from the "Results" section and added two new paragraphs to the "Discussion" section, where this point is discussed in detail. We hope that this clarifies the issue.

REVIEWER:
-. On page 9, "we applied Prot-SpaM to all available protein sequences from these 813 taxa. In addition, we ran Prot-SpaM on the protein sequences encoded by the 24 marker genes from Lang et al." <= For application of Prot-SpaM to two different type of datasets, were same selected spaced-word matches and same patterns used?

REPLY:
We used the same set of parameter values in all the test runs, i.e. a pattern length of 46, a weight of 6, and 5 different patterns. However, as mentioned in the manuscript, we are using probabilistic algorithm to calculated sets of patterns. Therefore, we generally have not the same pattern sets in different program runs. We made the pattern sets that we used in the test runs available, so the results can be reproduced (see our reply to the last question of Reviewer #4)

REVIEWER:
-. For Table 3, what is the unit of computational time (seconds/minutes/hours)?

REPLY:
This is mentioned in the legend: "runtime in seconds".

REVIEWER:
Major Comments:
-. Since authors describes a new alignment-free method for whole-proteome phylogeny, please include the existing alignment-free method developed specifically for whole proteome phylogeny ("Whole-proteome phylogeny of prokaryotes by feature frequency profiles: An alignment-free method with optimal feature resolution" by Jun et al, PANS 2010; 107:133-138") for the comparison.

REPLY:
We evaluate FFP in our study (e.g. Table 1 and 2) cite a paper on FFP. We now added a reference to Jun et al, 2010.

REVIEWER:
-. No discussion found about Table 3 which summarized computational time for Prot-SpaM, and other alignment-free methods.

REPLY:

Powered by Editorial Manager® and ProduXion Manager® from Aries Systems Corporation

We now discuss the run time in detail in the "Discussion" section.

REVIEWER:
-. Authors claims that Prot-SpaM generates more statistically meaningful trees than other alignment-free methods. But, I don't see description of statistical confidence on internal nodes. Please describe how to impose statistical confidence on internal nodes of trees generated by Prot-SpaM.

REPLY:
This may be a misunderstanding. We emphasize that our approach estimates the *number of substitutions* per sequence position based on a stochastic model of molecular evolution. So this is different from previous alignment-free approaches that output rather rough measures of sequence similarity/dissimilarity. But statistical confidence for specific nodes in a tree is a completely different thing. We are planning to work on this topic in the context of our approach, but this will be an entirely new project. It is not possible to do this within the current project. Therefore, we currently do not have confidence values for internal nodes.

REVIEWER:
-. On page 9, "There are some differences within the clades, though, that should be further investigated." <= please at least provide information of RF and Branch score distance between four trees in Figure 4.

REPLY:
The RF distances are given in Table 1. As explained below, we preferred to omit the "branch scores" altogether, since they only caused confusion (and branch scores are not commonly used to evaluate alignment-free methods, for good reasons).

REVIEWER:
-. In Figure 3, Did Prot-SpaM segregate E.coli from Shigella? The paper "Insights from 20 years of bacteria genome sequencing" published in Funct Integr Genomics, 2015; 15:141-161 (https://www.ncbi.nlm.nih.gov/pmc/articles/PMC4361730/) showed a tree which segregated E.coli from Shigella clearly using the method described in "Whole-proteome phylogeny of prokaryotes by feature frequency profiles: An alignment-free method with optimal feature resolution" by Jun et al, PANS 2010; 107:133-138. Please discuss about which alignment-free methods including Jun et al's method have a capability of segregating E.coli from Shigella.

Insights from 20 years of bacterial genome sequencing
www.ncbi.nlm.nih.gov
Since the first two complete bacterial genome sequences were published in 1995, the science of bacteria has dramatically changed. Using third-generation DNA sequencing, it is possible to completely sequence a bacterial genome in a few hours and identify ...

REPLY:
We used a reference tree from Zhou (2010). In this tree, Shigella is not clearly separated from E.coli. In fact, many authors argue that Shigella is not monophyletic, so it should not be separated in the tree. But since our paper is not on the evolution of Shigella, we do not want to comment on this, so we just compare our trees to a trusted reference tree (as is common practice in alignment-free papers).

REVIEWER:
-. On page 8, authors described analysis results of Wolbachia strains without presenting a tree. Please provide the tree with annotations according to discussion to support the analysis results. In the subsection of Wolbachia, authors described how to generate an alignment-based tree with Wolbachia II dataset. But, there is no discussion found about comparison results with other alignment-free methods for Wolbachia II dataset.

REPLY:
We now included a figure with the Wolbachia tree produced by our approach plus the reference tree. The results are now discussed.

REVIEWER:

Powered by Editorial Manager® and ProduXion Manager® from Aries Systems Corporation

-. Selected spaced-word matches and patterns are the most important factors for the method, Prot-SpaM. It seems that a length of l=46, a weight of w = 6, 40 don't-care positions and five patterns were used through the study. I don't see how authors reached these parameter values. Please describe the optimization procedure for these parameters, l, w, five patterns. For these optimized values, does selected spaced-word matches mean spaced-word matches with scores >0? Please describe meaning of 'selected' in "selected spaced-word matches".

REPLY:
Yes, this was missing in the initial submission. We used those parameter values since they worked well on a broad set of test data. To clarify this, we now added two tables with runtime and RF distances for different parameter values. Also, we clarified what "selected spaced-word matches" means. (It means score > 0 and one-to-one mapping of spaced-word occurrences).

REVIEWER:
Second, please clarify whether selected spaced-word matches with a length of l=46, a weight of w = 6, 40 don't-care positions, and five patterns fixed were for any dataset (simulated, prokaryotic, eukaryotic proteomes, protein sequences)? Please provide sets of selected string-word matches and patterns in Supplementary.

REPLY:
Yes, we used these parameter values in all test runs. It's not clear to us, however, what the reviewer means with "provide sets of selected string-word matches and patterns". We now published all pattern sets that we used, so the results can be reproduced.

REVIEWER:
Third, please describe computational time for defining selected spaced-word matches and patterns.

REPLY:
Patterns are calculated with rasbhari, the runtime of this program is described in detail in the original publication (Hahn et al., 2016). The runtime for "defining selected spaced-word matches" is difficult to specify, since selecting ("defining") spaeced-word matches and calculating the number of mismatches per position from the selected spaced-word matches is done in one single step. It's difficult to separate this in the runtime analysis. We think, what really matters is the overall runtime of the program, not so much the runtime for the single steps in of the program.

REVIEWER:
Fourth, Prot-SpaM uses only selected spaced-word matches which indicates fraction of proteomes are compared instead of whole proteomes when being built phylogenies. Please describe what fraction of proteomes on average by Prot-SpaM were being used for datasets discussed in the manuscript.

REPLY:
It is not entirely clear to us what "fraction of proteomes .. used for datasets" means. We used the full proteomes in our analyses. Does the reviewer want to know which fraction of the proteomes is covered by SW matches? We now added some data on the number of spaced-word matches that our program uses, from two extreme cases: one where a large number of sw-matches is selected (closely related sequences) and one with a small number of sw-matches (distantly related sequences).

REVIEWER:
-. In comparing Table 1 (RF distance) with Table 2 (Branch score distance), since Prot-SpaM captures evolutionary distance between two sequences unlike other alignment-free methods, Prot-SpaM should perform better than other alignment-free methods against alignment-based reference trees by Brach score distance. But, for example, trees by CVTree method were closer to alignment-based reference trees than trees by Prot-SpaM with branch-score distance for some datasets even though CVtree doesn't capture evolutionary distance at all. Please discuss about this issue.

REPLY:

Powered by Editorial Manager® and ProduXion Manager® from Aries Systems Corporation

For various reasons, the "branch score" distances caused confusion in our study, see other reviewer comments and our reply to them. Therefore, we prefer to omit the branch scores and to restrict ourselves to the RF distances which are commonly used in the literature (all other alignment-free papers use RF distances, we don't know any paper where branch scores are used to evaluate alignment-free approaches). In most examples, Prot-SpaM performed better than other alignment-free methods, and we now discuss this point in the "Discussion".

REVIEWER:
-. In the manuscript, validation step was solely dependent on alignment-based reference trees which sounds like that authors tried to develop an alignment-free method which produces trees most resemblance to alignment-based tree. For example, a reference tree of 813 prokaryotes was based on 24 marker genes and was found to be very similar to be 16S rRNA-based tree. Then, according to the validation procedure, Prot-SpaM tried to prove that the method produces a tree most resemblance to a 16S rRNA-based tree which does not require orthologous analysis and might not be computationally inferior to Prot-SpaM. Furthermore, even though Prot-SpaM pairwise distance captures evolutionary distance, the distances on the Prot-SpaM tree cannot be interpreted with substitution rates since distance-based methods are only applicable. To investigate other advantages of Prot-SpaM, the method needs to be examined over taxonomic classification in comparison with other alignment-free methods
since taxonomic classification captures evolutionary information. Please examine a capability of taxonomic classification for the datasets discussed in the manuscript at least at the species level in comparison with other alignment-free methods.

REPLY:
Alignment-free approaches are certainly computationally more efficient than 16S rRNA-based approaches where one first has to identify these sequences and then construct an alignment. And it's not quite clear to us what the reviewer means with "taxonomic classification". We do this by comparing phylogenetic trees to reference trees, and this is what all papers on alignment-free methods are doing to evaluate new tools.

REVIEWER:
-. The following error messages occurred when compiling the code downloaded from "https://github.com/jschellh/ProtSpaM" by 'make'. It seems that sysinfo.h is not provided.

```
mkdir -p obj
g++ -fopenmp -c -Wall -std=c++11 -I ./include main.cpp -o obj/main.o
In file included from ./include/speedsens.hpp:6:0,
                 from ./include/rasbcomp.hpp:9,
                 from ./include/rasbhari.hpp:8,
                 from ./include/rasbimp.hpp:4,
                 from main.cpp:26:
./include/sensmem.hpp:7:25: fatal error: sys/sysinfo.h: No such file or directory
 #include "sys/sysinfo.h"
                         ^
compilation terminated.
make: *** [obj/main.o] Error 1
```

REPLY:
The error message seems to be produced by the program rasbhari, which our software calls. It seems that this error occurs if one tries to run the program on Windows. The current version of the program, however, is designed to run on Linux. We now made clear that our program should be used under Unix/Linux.

==

Reviewer #4:

REVIEWER:
The paper presents Prot-SpaM, a method to construct phylogenetic trees using

proteome data based on the alignment-free spaced-word approach. The proposed method estimates pairwise distances for the set of sequences by computing the mapping of spaced words. The authors evaluate their approach using simulated data and also demonstrate the approach using for different real datasets, ranging from microbial data to plant proteomes. The authors also provide a rigorous method to select the threshold parameter using the spaced-word match histogram. Using the experiments using simulated data, they show Prot-SpaM proves a better approximation w.r.t to the expected distance computed by the Kimura model. The authors have made the software freely available at github, and is easy to build and run on a typical linux machine.

REPLY:
Thank you.

REVIEWER:
1. The key difficulty when using spaced-words approach in practice is the selection of the key parameters (a) pattern weight (b) number of don't care positions, (c) number of patterns and (d) minimum threshold. The paper includes a very good discussion on the selection of minimum threshold using spaced-word histogram. However, there is no guidance on how the other parameters can be selected. Even though all the experiments use the default parameters, a brief discussion on the effect of these parameters can be useful for practitioners.

REPLY:
We agree, this was missing in the previous version of the manuscript. We now added two tables with different parameter values to clarify why we are using our default values (see also reply to Reviewer 3)

REVIEWER:
2. The paper mentions that program uses 5 patterns, by default. Is there any reason for this default ?

REPLY:
With the multiple-pattern option, the quality of the results on our benchmark data was the same as with the single-pattern approach, in view of the RF distance. However, the estimated distances are more stable with the multiple-pattern approach. We added a figure to show this. We clarified this point in the manuscript; for large data sets users can reduce the number of patterns to speed-up the program.

REVIEWER:
3. The paper mentions that the pattern set is identified using a probabilistic algorithm, rasbhari, and hence, for two different runs with the same parameters can result in two different pattern sets. How does this affect the output trees ? In the experiments with real datasets, the authors provide a single RF distance in the Table 1. How does the RF distance varies w.r.t the probabilistic pattern set selection ?

REPLY:
This is a good point. For the previous manuscript, we did a *single* program run on each data set. Now, we did 100 program runs for each data set and report the average RF distances and standard deviations (except for the large bacterial data set. Here, we did only one program run because of the long run time for this data set).

REVIEWER:
4. In the descriptions of the algorithm (specifically in the third and sixth paragraphs of the introduction), the authors use the word 'alignment' to specify the spaced word pattern matches. I think this use of alignment description here is a bit confusing because pattern matching with spaced words is scored only in terms of the hamming distance.

REPLY:
We think, aligning two equal-length substrings without gaps is still an "alignment", even if this is a trivial sort of alignment (The term "gap-free alignment" is often used in this context in the literature). Therefore we'd prefer to leave the term "alignment" as it is. (BTW we are not using the Hamming distances, but the sum of scores of "aligned"

amino-acid residues).

REVIEWER:
5. For experiments with simulated data (Section 3.1), the authors mention that they evaluated 1000 sequence pairs for each distance data. They also mention that they ran with both default parameters and with only one pattern. Does the error bars (standard deviations) plotted in figure 2 include results from both runs or only the default runs ?

REPLY:
The figure shows the results for the default parameter values, as is mentioned in the legend. We now added a figure with results where we used only one pattern and compared this to the default (5 patterns). This shows, that the results are more stable with multiple patterns.

REVIEWER:
6. For the microbial phylogeny, the authors mention that the tree obtained with Prot-SpaM contains essentially the same clades. It is very difficult to see from Figure 3, in terms of the clades, how different the tree constructed by kmacs or ACS from reference tree, which has lower RF distances compared to Prot-SpaM ?

REPLY:
Does the reviewer mean Figure 4 of the bioRxiv preprint (now Figure 5 of the revised manuscript)? Because of the large number of taxa, it is indeed difficult to compare these trees by looking at them. We added larger figures of the trees calculated by our and competing methods to the supplementary material.

REVIEWER:
7. While reporting the runtime, it is instructive to provide the type of machine the software were run. Also, it seems the implementation available at https://github.com/jschellh/ProtSpaM has multi-threaded capability, but the table 3 doesn't mention if it was run using single thread or multiple threads.

REPLY:
Done (added to first paragraph of "Resuts" section).

REVIEWER:
8. While the paper says that the use 5 patterns with w = 6 as default, the software in github appears to use only one pattern with w = 8 for default. Is there a reason for this discrepancy ?

REPLY:
Thank you, this was an error, we corrected this on github.

REVIEWER:
I also reviewed the supplementary data and the documentation seems sufficient to reproduce the results. Since there is probabilistic algorithm to select of the pattern sets, it would be useful to have a few seed patterns for the simulated data, if possible. Also, it can be useful for future research if the authors can provide all the reference trees along with the supplementary data.

REPLY:
We made the pattern sets available that were used in the test runs. Also, we added the following options to our software: (a) output the pattern set used in a program run to a separate file. (b) Use a user-defined pattern set instead of the pattern set generated by rasbhari. This way, it is possible too reproduce program runs with exactly the same pattern sets (which was indeed somewhat difficult with the previous version of the program where in each program run a new pattern set was used that was generated probabilistically)

==

Reviewer #5: Genenal comments:

REVIEWER:
In this manuscript, Leimeister et al. present a novel tool for alignment-free phylogeny reconstruction from protein sequences.
Overall, the manuscript is well structured, written in clear language and is easy to follow. Tables and plots are used adequately to represent experimental results (see some specific suggestions below).

REPLY:
Thank you.

REVIEWER:
The presented method appears to be a rather minor modification of the existing FSWM tool (Leimeister et al. 2017, Bioinformatics). In particular, the only differnces seem to be: (1) using a different input alphabet (aminoacids) and thus a different scoring matrix (BLOSUM62), (2) using multiple patterns, and (3) using Kimura distance correction. Given that optimizations (2) and (3) can also be applied for genomic/DNA sequences, is it really justified to implement these minor modifications as a separate software tool? Arguably, having a single tool which can handle both DNA and protein sequences would be more convenient for the users while also simplifying software maintenance.

REPLY:
Due to technical issues, it is not trivial to have both programs in one program code, just with different options for DNA and proteins. One thing is, for example, the different alphabet size for DNA and proteins. But we fully agree that it would be preferable to have one single piece of software, and we will see if we can achieve this in the future.

REVIEWER:
Despite limited methodological novelty, ProtSPAM could be still a valuable addition to the field if it can achieve significant improvements over existing methods in terms of the tree reconstruction accuracy and/or speed. Unfortunately, presented experimental results cannot convincingly demonstrate this. When compared to FSWM, ProtSPAM is significantly more accurate only on 1 dataset out of 6 (Tables 1 and 2). And even on this dataset (813 prokaryotes), ProtSPAM showed higher topological error (RF distance) than 3 competing methods (CVTree, kmacs and ACS). However, even the best method (kmacs) inferred a tree which is very dissimilar to the reference (relative RF distance rRF=0.54). Therefore, differences between e.g., kmacs (rRF=0.54), ProtSPAM (rRF=0.63) and FSWM (rRF=0.83) are remarkable but less important, since all methods can be considered very inaccurate. Moreover, these results can be confounded by the instability of the reference tree itself, which is quite common for large trees inferred from a limited set of genes (see Figure 6 in Lang et al. 2013). Additionaly, it is not clear whether we can expect high level of similarity between trees built from whole proteomes and ribosome proteins, since the latter one is in turn quite different from the 16S tree (rRF=~0.5, same figure).

Given the above inherent problems with empirical datasets, I suggest to complement ProtSPAM evaluation with benchmarks on simulated data. In addition to the known reference tree, simulation allows to freely variate number of taxa, genome/proteome size and substitution rates, and thereby to compare performance of alternative methods more systematically. I can also recommend (Zhou 2017, https://academic.oup.com/mbe/article/35/2/486/4644721#113627412) for a decent set of thoroughly analyzed empirical phylogenomic datasets.

REPLY:
(a) If simulated data are used, the produced distances can be evaluated *directly*, this is what we have done. The disadvantage of simulated data is that they depend on the parameters used in the simulation. Therefore, we also added real data, as is common practice. On the real data, distances cannot be evaluated directly, so the evaluation is done *indirectly* by tree inference. We don't see why we should evaluate our program *indirectly*, considering the produced trees, on simulated data, where a direct evaluation of the distances is possible.
(b) Thanks for making us aware of the Zhou paper, this looks very useful. We now added one challenging benchmark set from this paper, namely the metazoan data set from Borowiec et al.

Powered by Editorial Manager® and ProduXion Manager® from Aries Systems Corporation

(c) We made some mistakes in the previous evaluation which are corrected now. The table with the RF distances shows that our method is superior to other alignment-free methods on whole proteomes. It is only slightly outperformed by other methods on selected proteins in the Wolbachia I data sets, we now address this point in the "Discussion".

REVIEWER:
In terms of computational speed, ProtSPAM seems to drastically outperform FSWM and has runtimes comparable to other methods which operate on protein sequences. Although >100x runtime difference between two highly similar methods looks somewhat surprising (see below), if this speedup is real and consistent, it could be a major argument in favor of ProtSPAM.

REPLY:
Thanks. Please note that FSWM runs on whole genomes while Prot-SpaM runs on proteomes. Since for eukaryotes, proteomes are much smaller, this is the reason why Prot-SpaM is faster. We discuss this now in the "Discussion".

REVIEWER:
In summary, the authors should try to make it clear under which conditions ProtSPAM outperforms other methods (high sequence divergence? large trees? large genomes?), and support this claim by convincing experimental results.

REPLY:
The focus of this paper is on the new algorithm to estimate distances from whole proteome sequences. We used various benchmark sets to evaluate our approach, as is common in the field, and we discuss runtime and accuracy in different situations. We now also discuss in more detail where our approach is superior to other methods and why we think this is the case. In short: it is not necessary to select orthologous genes/proteins first. However, we did not perform a comprehensive study to see under which conditions which method may perform better or worse. This would be beyond the scope of the present study.

REVIEWER:
- Figure 1: In my opinion, plots for empirical datasets (C and D) do not quite support authors' choise of the alignment score cutoff (T=0). Furthermore, the optimal cutoff value will probably depend on k-mer length/weight, as well as on the substitution matrix selected. It appears more reasonable to use an adaptive cutoff T, derived from the spamogram for the particular dataset under analysis and specific substitution matrix, seed length etc.

REPLY:
We now added a table where different values for T are used, to see how this parameter affects the resulting trees. For some of the data shown in the manuscript, a different cut-off value might be indeed a small advantage. But in some cases, the program would work better with a larger cut-off value T, while in other cases, a lower value of T would improve the results. So it is not at all obvious, how optimal threshold values can be found automatically. A systematic study of this issue is beyond the scope of the present paper. And we think that for our benchmark program runs, our threshold of T=0 works well enough in terms of the resulting trees. The figure shows that, by and large, we can well distinguish between random and homologous spaced-word matches, even with our threshold T=0.

REVIEWER:
- P4L12: can we set a fixed random seed to ensure reproducibility?

REPLY:
Yes, see our reply to the last comment from Reviewer #4. We added all pattern sets that we used in the test runs to the "supplementary material". Also, we now have an option to use a user-defined pattern set, instead of generating pattern sets probabilistically with rasbhari. This way, one can exactly reproduce the results on our benchmark data, using the same pattern sets.

REVIEWER:

- experimental setup: please add test system configuration, program versions and command lines used

REPLY:
See above, we added the system configuration (first paragraph of "Results"). We added program versions where available, but not all programs that we tested have version numbers.

REVIEWER:
- it would be helpful to have a table summarizing dataset characteristrics (#taxa, genome/proteome size, reference tree used)

REPLY:
Done

REVIEWER:
- Figure 2: could it be that extremely poor distance estimates given by competing methods are - at least partially - due to a normalization artifact? The respective curves suggest that the distances reported by other methods might be on the logarithm scale. This would also explain why their branch length estimates in the empirical phylogentic trees are only moderately worse than those of ProtSPAM (Table 2).

REPLY:
This is a very interesting question. The question is if there is a possible "transformation" that would turn the usual word-frequency distances into estimates of "real" distances, i.e. substitutions per position. More generally, it would be interesting to know why word-frequency methods lead to reasonable tree topologies, even if these distances are far from the "real" distances. But this point is clearly not within the scope of our study, this would be a completely new project, not directly related to our present paper. (Similar questions are discussed in Morgenstern et al., 2015).

REVIEWER:
- Table 1: please (additionally) report realtive Robinson-Foulds (RF) distances since those are much easier to interpret

REPLY:
Done

REVIEWER:
- Table 2: why are both "E. coli" and "Brassicea" datasets missing from this table?

REPLY:
Table 2 in the previous version of the manuscript reported the "branch score" distances to reference trees. This assumes that the reference trees have branch lengths. For E.coli and for the plant data sets, however, we had only topologies as reference, no branch lengths. Since the "branch scores" caused also other confusion, we decided to omit them completely (see our reply to the next reviewer comment).

REVIEWER:
- Table 2: could authors please describe how they normalized the branch lengths? even for the methods that use expected number of substituions per site as their branch length unit, using different data types (DNA/AA) and genomic regions (conserved genes vs. non-coding regions) will yield different estimates.

REPLY:
We did not normalize the branch lengths in the previously included table. Generally, the branch-score distances that we reported (Table 2 of the previous manuscript) caused some confusion: (a) since the other alignment-free methods that we evaluated (except for FSWM) do not attempt to estimate phylogenetic distances, it seems unfair to evaluate their distances under the "branch-score" metric, even if they are normalized. (b) For some of the reference trees, we only have their topologies not the branch lengths, so the branch-score distance is not applicable. For these reasons, all other papers in the field only use the RF distances. We think it is best to do the same and to omit the branch scores altogether.

Powered by Editorial Manager® and ProduXion Manager® from Aries Systems Corporation

REVIEWER:
- Table 3: are these single-core runtimes, or was multi-threading used for some/all of the programs?

REPLY:
We used multi-threading (40 threads) for those programs that were able to use multiple threads. This is now clarified in the main text and in the table legend.

REVIEWER:
- Table 3: on "Brassicea" and "813 projaryotes" datasets, ProtSPAM runs ~10-300x faster than FSWM. This is a bit surprising, given that two methods are highly similar. Could authors please provide an explaination for this remarkable difference?
Is it due to much smaller proteome vs. genome size? Due to fewer word matches? Or inefficient implementation of FSWM? Please clarify.

REPLY:
The main reason for the difference in speed is the difference between genome and proteome size for eukaryotes (see above). This is now discussed in the "Discussion".

REVIEWER:
- Figures 3 and 5: whenever possible, please use identical branch length scaling factors for easier interpretation

REPLY:
Some trees were taken from the literature, so here it was difficult to scale them exactly.

REVIEWER:
- P9L51, Figure 4 and 800+ prokaryotes tree: Color-coded clades correspond to bacterial phyla, that is, major groups that have split very early and have high sequence divergence. It is therefore not surprising that all methods were successfull in recovering these well-established clades. The difficult parts of this phylogeny are the relationships within the clades as well as the branching order of the phyla (deep splits).

REPLY
We agree. But given the size of the data set, this is not easy to visualize. We add a figure to the supplementary material.

REVIEWER:
- one interesting question that remains unanswered by this study is which input data type is better suited for alignment-free phylogenetic inference: whole-proteome, whole-genomes or whole-exome/-transcriptome? This appears to be relevant given that proteomes might be less readily available compared to genomes, as exemplified by the fact that the authors had to reduce some of the empirical datasets due to missing proteomes. On the other hand, direct proteome vs. exome comparison could deconvolve the effects of more conserved characters (AA vs. DNA) and more conserved regions (coding vs. non-coding).

REPLY:
In general, protein sequences are used for distantly related species, while DNA sequences are more suitable for closely related sequences. This was the whole motivation for our approach, as we mention in the "Introduction". It would be indeed very interesting to study this point in more detail, i.e. to find out more precisely in which situations whole-proteome sequence comparison is superior to whole-genome comparison with our spaced-words approach. But a systematic study of this question is beyond the scope of the present paper.

REVIEWER:
- would the authors please consider depositing all relevant supplementary files (empirical and simulated sequences, trees, scripts/command lines, results obtained by different programs etc.) in a public repository?

REPLY:

Powered by Editorial Manager® and ProduXion Manager® from Aries Systems Corporation

We uploaded this to http://projects.gobics.de/data/protspam/paperData.tgz We will add a public link to github.

REVIEWER:
- supplementary information provided for the review seems to be incomplete: e.g., I cannot find reference trees for most datasets, as well as simulated sequences for substitution rates > 1.0

REPLY
We added this.

==
==

ANONYMOUS REVIEWER WITH USEFUL COMMENTS (Reviewer #2):

REVIEWER:
1) It will be helpful if you provide a user friendly .exe of the program for biologists with no knowledge in programming.

REPLY:
We think that a user-friendly web-interface would be most useful for wet-lab biologists, so this has priority for us. Most experienced computational biologists are working under Linux/Unix anyway. But we will consider providing an .exe in the future.

REVIEWER:
2) Abstract: " Prot-SpaM can be used to calculate
high-quality phylogenetic trees from whole-proteome sequences in
a matter of seconds or minutes and often outperforms other alignmentfree
approaches." you should provide the size of the trees that are computed in seconds or minutes.

REPLY:
Done. It's now "dozens of whole proteins" in the abstract. More details can be found in in the main text.

REVIEWER:
3) I suggest to mention in the intro and discussion the fact that in bacteria there are frequent HGT events and thus the accurate evolutionary model is network and not a tree.

REPLY:
This is certainly true and could be mentioned. However, since our goal is to estimate distances without full sequence alignments, we prefer not to address this topic as it could distract from the main content the paper.

REVIEWER:
4) "Thus, for distal species, it may be hard to detect similarities
between genes at the DNA-sequence level, while homologies may be
still detectable among protein sequences." It is important to discuss that fact that whole genomes include additional information (e.g. regulatory signals) that does not exist in the proteome.

REPLY:
We fully agree. But here, we discuss the advantage/disadvantage of DNA/proteins depending on the evolutionary distances. So we want to focus on this point.

REVIEWER:
5) The algorithm sound similar to previous papers including previous papers of the authors (e.g. refs 38,23, 65). You need to clearly explain the improvement/changes in the methodology. Specifically, what is the different between your " previous version of our program for nucleic-acid sequences" and the current one.

REPLY:

Powered by Editorial Manager® and ProduXion Manager® from Aries Systems Corporation

	It's a completely new implementation for whole-proteome sequences. This involves technical differences due to the different alphabet size but, above all, because of different substitution matrices, parameter settings, program evaluation etc. So that's the improvement/changes compared to our previous paper. We think that this point is already addressed in the manuscript.<br><br>REVIEWER:<br>6) " To achieve this, we use the same greedy algorithm that we described in our previous paper [38]. " .." rasbhari uses a hill climbing<br>algorithm to reduce the overlap complexity ". For a better readability, please provide more details related to the different part of the method (even if they are published in a different paper).<br><br>REPLY:<br>Done. We shortly outline the greedy algorithm.<br><br>REVIEWER:<br>7) "By default, our program uses a set of 5 patterns" why 5 ?<br><br>REPLY:<br>See our comments above. This was indeed missing in the previous manuscript; we discuss this point now in more detail and added test results with single patterns in addition to the 5-pattern sets.<br><br>REVIEWER:<br>8) Table 1: other methods are better in most cases. Is this true that methods such as ACS tend to outperform your method for very large datasets?<br><br>REPLY:<br>We don't think that other methods are "better in most cases". As mentioned above, there were mistakes in the previous manuscript for the RF distance on the "Wolbachia II" data set (should be between 18 and 20, but was 28 in the previous table). ACS, CVtree and kmacs still outperforms Prot-SpaM on the large prokaryotic data set, but not on the other full-proteome data sets. The selected proteins from Wolbachia I are a special case which we now discuss in more detail in the "Discussion".<br><br>REVIEWER:<br>9) Table 3: can you discuss the features that effect the running time of your method and other methods. There is no datasets where your method is faster than all other methods.<br><br>REPLY:<br>We now compiled the program with a different option and used multiple cores. Therefore, the runtime is now much better than what we reported in the previous version of the paper. The runtime can be further improved, if the "single-pattern" option is used instead of the 5 patterns that we use by default. This does not affect the quality much. We now discuss this point in the "Discussion".<br><br>REVIEWER:<br>10) Maybe you can improve the biological discussion related to the results phylogenic trees.<br><br>REPLY:<br>We have to say, this comment is a bit vague. We discuss the results on Wolbachia in some detail, and we added two paragraphs on the results on whole proteomes vs. selected proteins in the "Discussion". But the focus of this paper is a new software tool, not on phylogeny as such.<br><br>REVIEWER:<br>11) It is very hard to see anything in figures 3-5.<br><br>REPLY:<br>We agree. Therefore, we added a larger figure to the supplementary material.
**Additional Information:**	

Question	Response
Are you submitting this manuscript to a special series or article collection?	No
**Experimental design and statistics**<br><br>Full details of the experimental design and statistical methods used should be given in the Methods section, as detailed in our Minimum Standards Reporting Checklist. Information essential to interpreting the data presented should be made available in the figure legends.<br><br>Have you included all the information requested in your manuscript?	Yes
**Resources**<br><br>A description of all resources used, including antibodies, cell lines, animals and software tools, with enough information to allow them to be uniquely identified, should be included in the Methods section. Authors are strongly encouraged to cite Research Resource Identifiers (RRIDs) for antibodies, model organisms and tools, where possible.<br><br>Have you included the information requested as detailed in our Minimum Standards Reporting Checklist?	Yes
**Availability of data and materials**<br><br>All datasets and code on which the conclusions of the paper rely must be either included in your submission or deposited in publicly available repositories (where available and ethically appropriate), referencing such data using a unique identifier in the references and in the "Availability of Data and Materials" section of your manuscript.	Yes

Powered by Editorial Manager® and ProduXion Manager® from Aries Systems Corporation

Have you have met the above requirement as detailed in our Minimum Standards Reporting Checklist?	

Powered by Editorial Manager® and ProduXion Manager® from Aries Systems Corporation

Manuscript
Click here to access/download;Manuscript;protspam.pdf
Click here to view linked References

Prot-SpaM: Fast alignment-free phylogeny reconstruction based on whole-proteome sequences

Chris-Andre Leimeister[1,*], Jendrik Schellhorn[1,*], Svenja Schöbel[1], Michael Gerth[2], Christoph Bleidorn[3,4], and Burkhard Morgenstern[1,5]

[1]University of Göttingen, Department of Bioinformatics, Goldschmidtstr. 1, 37077 Göttingen, Germany
[2]Institute for Integrative Biology, University of Liverpool, Biosciences Building, Crown Street, L69 7ZB Liverpool, UK
[3]University of Göttingen, Department of Animal Evolution and Biodiversity, Untere Karspüle 2, 37073 Göttingen, Germany
[4]Museo Nacional de Ciencias Naturales, Spanish National Research Council (CSIC), 28006 Madrid, Spain
[5]Göttingen Center of Molecular Biosciences (GZMB), Justus-von-Liebig-Weg 11, 37077 Göttingen
[*]Joint first authors

September 6, 2018

Abstract

Word-based or 'alignment-free' sequence comparison has become an active research area in bioinformatics. While previous word-frequency approaches calculated rough measures of sequence similarity or dissimilarity, some new alignment-free methods are able to accurately estimate phylogenetic distances between genomic sequences. One of these approaches is *Filtered Spaced Word Matches*. Herein, we extend this approach to estimate evolutionary distances between complete or incomplete proteomes; our implementation of this approach is called *Prot-SpaM*. We compare the performance of *Prot-SpaM* to other alignment-free methods on simulated sequences and on various groups of eukaryotic and prokaryotic taxa. *Prot-SpaM* can be used to calculate high-quality phylogenetic trees for dozens of whole-proteome sequences in a matter of seconds or minutes and often outperforms other alignment-free approaches. The source code of our software is available through *Github*: `https://github.com/jschellh/ProtSpaM`

1 Introduction

Evolutionary relationships between species are usually inferred by comparing homologous gene or protein sequences to each other. Here, groups of orthologous sequences have to be identified first, for which then multiple alignments are to be calculated. There are generally two different strategies of resolving phylogenies based on multiple alignments. In the so-called *supermatrix* approach, multiple sequence alignments of single genes or proteins are concatenated. A phylogenetic tree is inferred from the resulting matrix, *e.g.*, using *Maximum Likelihood* [63] or *Bayesian inference* [57]. Alternatively, gene or protein trees are inferred for every single multiple sequence alignment and the resulting phylogeny is inferred using *coalescent* models [46] or supertree [4] approaches.

All these steps are time consuming, and often manual intervention is required. Therefore, *word-based* or *alignment-free* alternatives have been proposed recently, which are much faster and which require much less data preparation. Most alignment-free methods compare the *word composition* of sequences [11, 20, 31, 59, 65, 69], with some approaches also considering background word frequencies [53, 54, 60, 70], see [55] for a review of these latter approaches. More recently, the *spaced-word* composition of sequences has been used for sequence comparison [32, 42, 48, 50]. Other alignment-free methods are based on the so-called *matching statistics*, that is they use the length of maximal common subwords [12, 68]. This has been extended to maximal common subwords with a certain number of mismatches [43, 52, 66, 67]. Alignment free approaches have been recently reviewed in detail [2, 27, 75].

Accurate alignment-free tools are urgently needed because of the huge volume of data generated by new sequencing techniques. Another advantage of alignment-free methods, compared to alignment-based approaches, is the fact that they can be applied to incomplete data, for example to unassembled sequencing reads or to partially sequenced genomes [18]. Note that some of the so-called 'alignment-free' approaches are based on comparing words of the input sequences to each other. So, strictly spoken, they are not 'alignment-free' since they align these words to each other. The term *alignment-free* is used nevertheless by most researchers, since these word-based approaches circumvent the need to calculate full pairwise or multiple alignments of the sequences under study.

The above mentioned approaches to alignment-free sequence comparison calculate ad-hoc measures of sequence similarity or dissimilarity. They are not based on stochastic models of molecular evolution, and they do not try

to estimate distances between sequences in a statistically rigorous way. More recently, some alignment-free approaches have been proposed that are based on explicit models of DNA evolution. These methods are able to estimate the number of substitutions per site that have happened since two nucleic-acid sequences have evolved from their last common ancestor [14,28,29,44,47,72].

A main application of alignment-free approaches is comparison of whole *genomes*. Consequently, most alignment-free methods have been designed to work on DNA sequences. If distantly related species are studied, though, phylogenetic trees are usually inferred from protein sequences rather than from DNA sequences. The reason for this is that protein sequences are more conserved than DNA sequences, as synonymous substitutions are not visible in proteins. Thus, for distal species, it may be hard to detect similarities between genes at the DNA-sequence level, while homologies may be still detectable among protein sequences. It is therefore highly desirable to have accurate alignment-free software tools that work on protein sequences, in addition to the available tools for DNA sequence comparison. Generic word-frequency methods can be applied to both DNA and protein sequences; the program *FFP*, for example, has been used to whole-proteome comparison [35]. As mentioned above, however, these methods do not estimate phylogenetic distances in a rigorous way. So far, there are no alignment-free approaches available that can accurately estimate evolutionary distances between protein sequences.

In this paper, we propose an alignment-free method which estimates the number of substitutions protein sequences since they evolved from their last common ancestor. Our approach is based on *Filtered Spaced Word Matches (FSWM)*, a concept we introduced recently for whole-genome sequence comparison [44], see [28,72] for related approaches. We call the implementation of this new approach *Proteome-based Spaced-Word Matches (Prot-SpaM)*. The basic idea is to use gap-free pairwise alignments of fixed-length words with matching amino-acid residues at certain pre-defined positions. Such *spaced-word matches* can be rapidly identified and, after discarding random background matches, the remaining ‘homologous’ spaced-word matches can be used to estimate the phylogenetic distance between two taxa. To our knowledge, this is the first approach that accurately estimates evolutionary distances between protein sequences without the need to calculate full sequence alignments.

To evaluate our approach, we used simulated protein sequences and real-world whole proteomes. Test runs on the simulated sequences show that our distance estimates are very close to the true distances, for distance values of up to around 2.0 substitutions per sequence position. On the

real-world sequences, we evaluated our approach indirectly, by phylogenetic analysis, as is common practice in the field. We used *Prot-SpaM* to estimate pairwise distances for various sets of taxa, and we applied the *Neighbor-Joining* algorithm [58] to calculate phylogenetic trees from the resulting distance matrices. These trees were finally evaluated by comparing them to reference trees that were determined by standard methods and can be considered to be reliable. We show that the trees obtained with our approach are often of high quality, and they are generally ~~very~~ more similar to the respective reference trees than trees generated with other alignment-free approaches.

2 Method

We consider sequences over an alphabet $\mathcal{A}$. In this paper, $\mathcal{A}$ consists of 20 characters representing the 20 different amino acids. *Prot-SpaM* is based on so-called *spaced-word matches* between sequences. For a *wildcard character* '$*$' with $* \notin \mathcal{A}$ and a binary pattern P of length ℓ – *i.e.* for a length-ℓ word P over $\{0,1\}$ –, a *spaced-word* with respect to P is a length-ℓ word W over the alphabet $\mathcal{A} \cup \{*\}$ such that $W(i) = *$ if and only if $P(i) = 0$. An index $i \in \{1,\ldots,\ell\}$ is called a *match position* of P or W, respectively, if $P(i) = 1$, and a *don't care position* otherwise. The number of *match* positions in a pattern or spaced-word is called its *weight* w. We say that a spaced word W with respect to P occurs in a sequence S at some position i if one has $W(k) = S(i+k-1)$ for all $k \in \{1,\ldots,\ell\}$ with $P(k) = 1$ – *i.e.* for all *match positions* of P.

Moreover, we say that there is a *spaced-word match* w.r.t. P between two sequences S_1 and S_2 at (i_1, i_2) if the same spaced word w.r.t P occurs at position i_1 in S_1 and at position i_2 at S_2. In other words, there is a spaced-word match between S_1 and S_2 at (i_1, i_2), if and only if one has $S_1(i_1 + k - 1) = S_2(i_2 + k - 1)$ for all match positions k of P. Below is an example for a spaced-word match between two sequences S_1 and S_2 at $(2,3)$ with respect to the pattern $P = 1100101$; the spaced word $TN**D*P$ occurs at position 2 in S_1 and at position 3 in S_2:

S_1:		T	T	N	Q	I	D	L	P	P	C	Y	N
S_2:	A	C	T	N	L	I	D	I	P	Q	N		
P:			1	1	0	0	1	0	1				

Similar to our original *FSWM* approach, we estimate distances between protein sequences based on selected spaced-word matches between them,

with respect to one or several pre-defined patterns. Distance values are obtained by comparing the amino-acid residues that are aligned to each other at the *don't-care* positions of the selected spaced-word matches. This is similar to estimating distances in standard alignment-based approaches – the only difference to those standard approaches is that we are using *don't-care positions* of spaced-word matches instead of full sequence alignments.

To estimate distances in this way, one has to make sure that only those spaced-word matches are selected that represent *homologies*, *i.e.* that the involved spaced-word occurrences go back to the same origin in the last common ancestor of the two proteins that are compared. To distinguish such 'homologous' spaced-word matches from random background matches, we calculate a *score* for each spaced-word match using the BLOSUM62 substitution matrix [30]. Similar to the previous version of our program for nucleic-acid sequences, we define the *score* of a spaced-word match as the sum of substitution scores of the aligned amino acids at the *don't-care* positions. Based on this score, our algorithm decides if a spaced-word match is homologous or not: if its score is below a certain threshold T, then a spaced-word match is considered a random match and is not further considered. As default we use a threshold value of $T = 0$. To see that this threshold accurately separates homologous from background spaced-word-matches, one can plot the *number* of spaced-word matches with a score s against s, see Figure 1; we call such a plot a *Spaced-word-Match histogram* or *spamogram*, for short. In these plots, two peaks are typically visible, a peak on the right-hand side representing *homologous* spaced-word matches and a peak on the left-hand side representing *background* matches. By default, we are using patterns with a weight of $w = 6$ and with 40 *don't-care* positions, *i.e.* with a length of $\ell = 46$.

Moreover, we use a one-to-one mapping of spaced-word occurrences. Note that, if sequences S_1 and S_2 are compared and a spaced word W occurs n time in S_1 and n' times in S_2, than this gives rise to a total of $n \times n'$ spaced-word matches. Taking all these spaced-word matches into account for phylogeny reconstruction, would over-emphasize repeated regions where the same spaced words occur multiple times. Instead of using all possible spaced-word matches, we therefore use a one-to-one mapping of spaced-word occurrences in the compared sequences. That is, we ensure, that each spaced word occurrence is involved in most one of the selected spaced-word matches. Formally, if there are two spaced word matches, at (i_1, i_2) and at (j_1, j_2), respectively, then we can include both of them simultaneously in our list of selected spaced-word matches, only if $i_1 \neq j_1$ and $i_2 \neq j_2$ hold. To achieve this, we use the same *greedy* algorithm that we described in our

previous paper [44]: for a given spaced word W, we calculate the scores of all spaced-word matches involving W. We then select them one-by-one in descending order of their scores – always ensuring that each *occurrence* of W is used in at most one of the selected spaced-word matches.

Finally, in order to estimate pairwise distances between two input sequences, we consider the pairs of amino acids aligned to each other at the don't-care positions of the selected spaced-word matches. Here, we are using the *Kimura* model [38] that approximates the *PAM* distance [13] between sequences based on the number of mismatches per position. We are using these two different models since the *Kimura* model is commonly used to infer distances from the number of mismatches per position in alignments. The *BLOSUM* matrices, on the other hand, are standard in homology searching. Generally, our procedure to filter out background spaced-word matches is rather robust since the homologous and background regions in our *spamograms* can be easily distinguished as can be seen, for example, in Figure 1. So the choice of the substitution matrix to distinguish homologous from background spaced-word matches does not affect the results of our approach too much.

The accuracy and statistical stability of the described approach depends on the number of selected spaced-word matches: the more matches we obtain, the more accurate and stable the results of our method will be. To increase the number of spaced-word matches, the default version of our program uses *multiple* patterns, instead of one single pattern P. More precisely, we are using a set $\mathcal{P} = \{P_1, \ldots, P_m\}$ of m binary patterns, such that all patterns in $\mathcal{P}$ have the same length ℓ and the same weight w, but have their *match* and *don't-care* positions arranged differently; we then use spaced-word matches with respect to *all* patterns $P_i \in \mathcal{P}$. By default, our program uses sets of $m = 5$ patterns. To find suitable patterns sets, we integrated the tool *rasbhari* [25] into our implementation. *rasbhari* uses a *hill climbing* algorithm to optimize pattern sets according to a user-defined criterion. In our program, we use *rasbhari* to minimize the *overlap complexity* [33] of pattern sets. Note that *rasbhari* uses a probabilistic algorithm. It is therefore possible that different program runs of *rasbhari* return different pattern sets, even if the same parameter values are used. Consequently, different runs of *Prot-SpaM* on the same sequences and with the same parameter setting can produce slightly different distance estimates.

3 Results

To assess the quality of our new approach and to compare it to other alignment-free methods, we used artificially generated as well as real-world protein sequences. For the test runs we used the default parameters of our program, namely 6 match positions and 40 don't care positions – *i.e.* a total pattern length of 46 –, a threshold of $T = 0$ to discard background spaced-word matches, and sets of $m = 5$ patterns. We compared our program to four other alignment-free methods that can be run on protein sequences, namely *ACS* [68], *FFP* [35, 59], *kmacs* [43] and *CVTree* [53]. Here, we used version 3.19 of *FFP*, the other programs that we evaluated did not have version numbers at the time of writing. Since the original implementation of *ACS* is not publicly available, we used our own implementation of this approach by running *kmacs* with $k = 0$. The competing tools, too, were used with their default parameters. In addition to evaluating these tools on protein sequences, we ran *Filtered Spaced Word Matches* on the complete *genome* sequences of the same taxa. All test runs were done on a 10 x *Intel(R) Xeon(R)* CPU E7-4850 with 2.00*GHz* with 4 cores each summing up to 40 cores and 1000*GB RAM*.

3.1 Distance Estimation on Simulated Sequences

To evaluate the distances estimated by our program, we simulated sequences with the tool *pyvolve* [61]. *Pyvolve* simulates sequences along an evolutionary tree using continuous-time Markov models. It can use various substitution models such as *JTT* [34] and other models. Since there are no reliable stochastic models for insertions and deletions in protein sequences, the program produces indel-free sequences. We simulated pairs of sequences of length 100,000 with distances between 0 and 2 substitutions per position, in steps of 0.05, using the *JTT* model. To evaluate the estimated distance values, we generated 1,000 sequence pairs for each distance value and plotted the average of the estimated distances against the real *Kimura* distance of the respective sequence pairs, calculated with the program *protdist* from the *phylip* package [19]. To study the robustness of the estimated distances, we added error bars representing standard deviations to the plot. In addition to running *Prot-SpaM* with default parameters – *i.e.* with sets $\mathcal{P}$ of $m = 5$ patterns –, we did a second series of test runs with $m = 1$, *i.e.* with single patterns. Figure 2 shows the results of these test runs.

3.2 Phylogenetic tree reconstruction

Next, we applied the above alignment-free methods to calculate phylogenetic trees from real-world protein sequences. For four different groups of species, we downloaded all available protein sequences from *GenBank* [1]; within each group, we calculated all pairwise distances between the species. We used the distance matrices obtained in this way as input for *Neighbor-Joining* [58] and compared the resulting trees to reference trees which we assume to reflect the respective correct phylogeny for each group. The *Robinson-Foulds (RF)* distances [56] between the reconstructed trees and the respective reference trees are shown in Table 2.

As mentioned above, *Prot-SpaM* uses a probabilistic algorithm to generate pattern sets, so the results of different program runs on a sequence set can slightly differ, even if the same parameter values are used. We therefore performed 100 program runs on each data set, and Table 2 reports the *average RF* distances for these 100 program runs. An exception was the large prokaryotic data set where we only performed one single program run. Since *absolute RF* distances are not easy to interpret, Table 2 also reports the *relative RF* distances, which are obtained from the *absolute RF* distances by dividing by the maximum possible *RF* distance for a given data set. The maximal possible *RF* distances for a set of n taxa is $2 \cdot n - 6$ [9]. ~~the corresponding *branch score distances* are in Table ??~~. Program run times for the different approaches are shown in Table 3. Trees were visualized with *iTOL* [45]. *Neighbor-Joining* trees and *Robinson-Foulds* distances ~~and *branch score* distances~~ were calculated with the *phylip* package [19].

E. coli / Shigella

Our first data set consists of 29 strains of *Escherichia coli* and *Shigella*. For each strain, we were able to download about 4,000-5,000 protein sequences; the total size of this data set is around 41 *MB*. Figure 5 shows the reference tree that we used and the tree obtained with the algorithm described in this paper. The reference tree was published by Zhou *et al.* [74] and is based on a multiple sequence alignment of 2,034 core genes and a *Maximum Likelihood* method. As can be seen in Table 2, our approach produced a tree with a topology almost identical to the reference tree. All of the 100 program runs that we performed with *Prot-SpaM* produced the same tree topology; the *RF* distance between these trees and the reference tree was 4. The other alignment-free methods led to phylogenies with *RF* distances to the reference tree between 22 and 44; these trees are shown in the supplementary material.

Wolbachia

As a second test case for benchmarking, we analysed the phylogeny of *Wolbachia* strains, a group of Alphaproteobacteria which are intracellular endosymbionts of arthropods and nematodes [71]. Within *Wolbachia*, 16 distinct genetic lineages (supergroups) are currently distinguished (named by capital letters A-F and H-Q), which may differ in host specificity and type of symbiosis [24]. We re-analyzed a phylogenomic data set by [22], thereby focusing on relationships of strains within supergroups (*Wolbachia I*). A tree generated with *Prot-SpaM* from this data set is shown Figure 4.

For a second *Wolbachia* benchmarking data set, we analysed relationships between supergroups based on available (draft) genomes, see below (*Wolbachia II*). For within supergroup relationships (*Wolbachia I*), a program run of *Prot-SpaM* on the whole proteome recovered a tree which is largely congruent in topology and branch lengths in comparison to a phylogenomic supermatrix analysis of 252 single-copy orthologs which excluded genes which showed signs of recombination. A comparison based on *RF* distances showed that our new method out-competes other available alignment-free programs (Table 2). Interestingly, when only analysing the 252 ortholog data set of [22] instead of whole proteomes, *RF* distances become bigger, and other alignment-free method perform better (Table 2). ~~One interpretation is that misleading signal stemming from recombination events between *Wolbachia* strains is less problematic for alignment-free analysis then a reduction in he data set size~~

Analysing relationships between supergroups has been historically regarded as difficult phylogenetic problem [5, 23]. Analysing all annotated proteins from available genomes with *Prot-SpaM* supported the monophyly of all supergroups. Moreover, this analysis found the same *Wolbachia* strains basally branching as recent analyses suggested. Surprisingly, the phylogenomic supermatrix analysis analysis of 252 single-copy orthologs which excluded genes which showed signs of recombination of this data set recovered a topology which differs to previous study in not supporting the sister group relationship of supergroups A and B. In contrast, as found in previous analyses, the sister group relationship of supergroups A and B is supported by the *Prot-SpaM* analysis. The *Prot-SpaM* analysis also recovered some relationships between supergroups which differ from the topologies of our phylogenomic analysis or expectations from a recently published phylogenomic study [7]. However, it is known that supergroups differ in their base (and amino acid) composition, and it is currently unknown how this may impact alignment free methods. More sophisticated evolutionary models

could alleviate these differences in future studies. Nevertheless, in this test case *Prot-SpaM* also outperforms other alignment free methods when comparing the resulting phylogenetic tree with a phylogenomic analyses based on a concatenated supermatrix (Table 2).

For the *Wolbachia II* data set, we downloaded (if available) proteomes for all available *Wolbachia* draft and fully assembled genomes (47 in total, see supplementary material for details). Proteins for *Wolbachia* strains which were lacking this information on *NCBI GenBank* were derived from translations using *GeneMark* version 2.5 [3]. We predicted groups of orthologous genes between these proteomes using *Orthofinder* version 2.1.2 [17] running under default parameters. Single copy genes present in all analysed strains (83 in total) were aligned using *MAFFT* version 7.271 with the L-INS-i algorithm [37], and tested for evidence of recombination using the pairwise homoplasy index (*PHI*) [8] with window sizes of 10, 20, 30, and 50. Recombining loci were subsequently removed from the data set and the remaining loci concatenated using *FasConCat* version 1.0 [39]. The resulting supermatrix (68 loci, 20,787 amino acid positions) was subject to partitioned *Maximum Likelihood* analysis following best model and partition scheme selection in *IQ-TREE* version 1.6.2 [10, 36, 49];

For the whole-proteome sequences of the data set *Wolbachia I*, the *RF* distance to the reference tree was 6 for each of the 100 program runs. By contrast, for the 100 runs on the selected protein sequences of the same set of taxa, the average *RF* distance was 7.68, the standard deviation was 0.736. For *Wolbachia II*, the average *RF* distance was 19.62; the standard deviation was 0.89.

Large-scale microbial phylogeny reconstruction

In 2013, J. Eisen's group published a paper on the phylogeny of the microbial genomes that were available at the time [40]. As a basis of their study, they selected 24 single-copy marker genes and a non-redundant subset of taxa. To obtain such a subset, they used a greedy algorithm by M. Steel [64], making sure that marker genes from different taxa in the resulting subset had a distance to each other of at least 2 substitutions per 100 positions. This way, they obtained a non-redundant subset of 841 bacterial and archeal genomes from the more than 3,000 microbial genomes that were publicly available. Multiple sequence alignments of the marker genes were calculated with *hmmalign* [16] and were concatenated to a *supermatrix* which was used as input for the phylogeny programs *RAxML* [62] and *MrBayes* [57]. In addition, the authors used the Bayesian tree-reconciliation program *BUCKy*

[41] to the same set of marker genes. The trees they obtained with these different methods were found to be similar to trees obtained based on *16S RNA* genes.

To evaluate *Prot-SpaM*, we used the 841 microbial genomes from Lang *et al.* [40]. and downloaded all protein sequences from these taxa that were available through *GenBank*. For 28 out of the 841 taxa, we were unable to obtain protein sequences, so we obtained a slightly reduced subset of 813 taxa, compared to the taxa used by Lang *et al.* First, we applied *Prot-SpaM* to all available protein sequences from these 813 taxa. In addition, we ran *Prot-SpaM* on the protein sequences encoded by the 24 marker genes from Lang *et al.* and, finally, we applied our previous approach *Filtered Spaced Word Matches* [44] to the 841 genome sequences. The trees that we obtained with our different alignment-free approaches are shown in Figure 6, together with the *Maximum Likelihood* tree from [40] which we considered as a reliable reference. Clades from this reference tree are color-coded in Figure 6. As can be see from the color coding, the tree obtained with *Prot-SpaM* from the available protein sequences contains essentially the same clades as the reference tree. There are some differences within the clades, though, that should be further investigated (J. Eisen, personal communication). The *RF* distance between the tree obtained with *Prot-SpaM* and the reference tree was 1,020.

Plants

Next, we used a set of plant taxa [26] that has been previously studied by Hatje and Kollmar [26] and that we had already used in previous studies to evaluate alignment-free approaches to genome sequence comparison [42–44]. The data set that we used in these previous papers consisted of 14 brassicales species. In *GenBank*, however, the proteomes could be downloaded only for 11 of the 14 species, so we had to limit our test runs to these 11 species. Figure 7 shows the reference tree of the 14 original species, together with trees of the 11 species with available proteomes, calculated with the alignment-free methods that we evaluated in this paper. For the 100 program runs with *Prot-SpaM*, the average *RF* distance between the resulting trees and the reference tree from [26] was 0.82; the standard deviation was 0.9.

	# taxa	total size [MB]	Source
E. coli / Shigella	29	56.41	Zhou *et al.* [74]
Wolbachia I, 252 proteins	19	1.15	Gerth *et al.* [23]
Wolbachia I, whole proteomes	19	7.96	Gerth *et al.* [23]
Wolbachia II	47	14.78	See supplementary material
Plants	11	245.05	Hatje and Kollmar [26]
Prokaryotes	813	784.86	Lang *et al.* [40]
Metazoa	36	585.0	Borowiec *et al.* [6]

Table 1: Data sets used in this study to evaluate alignment-free methods, with number of taxa, total size and source of the reference tree.

Metazoa

Finally, we used a set of 36 proteomes from 34 *metazoan* and two choanoflagellate taxa. These taxa have been previously used by Borowiec *et al.* [6] to study the position of the Ctenophora within the phylogenetic tree of the metazoan kingdom. The same set of taxa has also been used in a study by Zhou *et al* [73] to evaluate *Maximum-Likelihood* programs for phylogeny reconstruction. As a reference tree, we used the tree published in [6]. The average *RF* distance of the *Prot-SpaM* trees to this reference tree was 27.1, with a standard deviation of 1.51.

Parameter values and number of selected spaced-word matches

Prot-SpaM has four major parameters which can be adjusted by the user: the *weight* w (=number of *match positions*) of the binary patterns and spaced words, their *length* ℓ, the number m of different binary patterns used by the program and the cut-off value T to separate homologous from background spaced-word matches. To see how these parameters influence the results of our software, and to find suitable default values, we ran *Prot-SpaM* with varying values of these four parameters. Here, we modified one parameter at

	Prot-SpaM	*FSWM*	*CVTree*	*FFP*	*kmacs*, $k = 10$	*ACS*
			RF distances			
E. coli / Shigella	4.00	6	24	40	42	38
Wolbachia I, 252 proteins	7.68	8	6	4	8	4
Wolbachia I, whole proteomes	6.00	6	8	16	8	12
Wolbachia II	19.62	20	44	54	26	16
Plants	0.82	0	6	8	2	6
Prokaryotes	1,020	1,348	886	1,452	880	960
Metazoa	27.1	-	40	62	30	36
			Relative *RF* distances			
E. coli / Shigella	0.08	0.12	0.46	0.77	0.81	0.73
Wolbachia I, 252 proteins	0.24	0.25	0.19	0.13	0.25	0.13
Wolbachia I, whole proteomes	0.24	0.19	0.25	0.50	0.25	0.38
Wolbachia II	0.23	0.23	0.50	0.61	0.30	0.18
Plants	0.05	0.00	0.37	0.50	0.12	0.37
Prokaryotes	0.63	0.85	0.55	0.90	0.54	0.59
Metazoa	0.41	-	0.61	0.94	0.45	0.55

Table 2: *Robinson-Foulds (RF)* distances and *relative RF* distances between trees generated with alignment-free methods and the respective reference trees for various sets of taxa, see the main text for details. Since *Prot-SpaM* uses a probabilistic algorithm, different program runs may produce slightly different results. Therefore, we performed 100 program runs on each data set and report the *average RF* distances, except for the large prokaryote data set where we did only one single program run. All programs were run on *protein* sequences or *whole proteomes*, respectively, except for *Filtered Spaced Word Matches (FSWM)*, which was run on *whole-genome sequences* of the same species (or on the gene sequences coding for the 252 selected proteins from *Wolbachia I*). We were unable to run *FSWM* on the whole genomes of the 31 *metazoan* species, since this data set was too large. Since the original implementation of *ACS* is not publicly available, we ran our own implementation, *kmacs*, with $k = 0$ instead.

	Prot-SpaM	*FSWM*	*CVTree*	*FFP*	*kmacs*, $k = 10$	*ACS*
E. coli / Shigella	55	110	125	10	2,518	193
Wolbachia II	19	68	46	9	5,302	135
Wolbachia I, 252 proteins	3	5	2	1	36	3
Wolbachia I, whole proteomes	11	22	21	2	178	26
Plants	464	1,107,720	365	17	17,693	850
Prokaryotes	5,502	244,139	5,492	1,929	915,635	123,520
Metazoa	1,719	-	1,973	43	151,612	9,512

Table 3: Program run time in seconds for different alignment-free approaches on our benchmark data sets. For the program runs we used multi-threading with 40 threads, see main text for more details.

a time, using the respective default values of the remaining three parameters. The results are summarized in Tables 4 and 5. As can be seen from these tables, there are no values for w, ℓ an T that work best for all data sets, but our default values seem to be a reasonable compromise. Using sets of $m = 5$ binary patterns does not improve the quality of the produced trees in terms of their *RF* distances to the reference trees, compared to program runs with single patterns. Table 3 shows, however, that the distance values estimated by *Prot-SpaM* become statistically more stable if multiple patterns are used.

The number of spaced-word matches in a pairwise sequence comparison depends on how similar the two sequences are to each other, see [48] for details. Consequently, the number of spaced-word matches that are selected by our program to estimate phylogenetic distances also depends on the degree of similarity between the compared sequences. We found two extreme cases with our test data, one in the *E. coli/Shigella* data set where most taxa are closely related to each other, and another one in the *Metazoan* data set that contains taxa with very large evolutionary distances. In the pairwise comparison of *E. coli O157:H7 strain EDL933* with *E. coli O157:H7 Sakai (EHEC)*, *Prot-SpaM* selected more than 6,000,000 spaced-word matches.

These two proteomes have less than 1,600,000 amino acids each, so in this case > 3.75 spaced-word matches per sequence position were selected. By contrast, less than 13.000 spaced-word matches were selected in the comparison of *Brugia malayi* and *Homo sapiens*. The latter proteome has a length of more than 75.000.000 amino acids, so here less than 0.00017 spaced-word matches per sequence position were selected.

4 Discussion

A number of so-called 'alignment-free' approaches have been proposed in recent years to rapidly calculate phylogenetic distances between genomic sequences. Earlier approaches are based on k-mer frequencies or on the length of common substrings; these approaches have been applied not only to DNA, but also to protein sequences. A draw-back of these methods is that they can only calculate rough measures of sequence similarity or dissimilarity, they do not estimate phylogenetic distances in a rigorous way. More recently, word-based methods have been developed that can accurately estimate phylogenetic distances between genomic sequences based on stochastic models of DNA evolution. One of these approaches is *Filtered-Spaced Word Matches (FSWM)*.

In this study, we introduced *Prot-SpaM*, a new implementation of *FSWM* to compare complete or incomplete *proteome sequences* to each other. To our knowledge, *Prot-SpaM* is the first tool that can accurately estimate phylogenetic distances between protein sequences without the need to calculate full sequence alignments. Our benchmark results show that distance estimates obtained with our approach are accurate for a large range of phylogenetic distances. Distances calculated with *CVTree, ACS, FFP* and *kmacs*, by comparison, are monotonously increasing with the number of substitutions between the compared sequences. The obtained distance values are far from proportional to the real distances, though, and they flatten out somewhere between 0.5 and 1.5 substitutions per position, see Figure 2. By contrast, *Prot-SpaM* estimates distances with high accuracy for up to around 2.0 substitutions per position. For higher distance values, the calculated distances become less stable, as can be seen from the error bars in Figure 2. Moreover, for large distances, our program tends to slightly overestimate distances.

In our program evaluation, *Prot-SpaM* produced high-quality trees and was superior to other alignment-free methods for the *E.coli/Shigella* and the plant data sets, as shown in Table 2. On the *Wolbachia* data sets, it still performed reasonably well and was again superior to competing approaches on

			E. coli/Shigella					
Weight w		**6**	8	10				
Runtime [s]		**55.4**	47.4	46.9				
RF distance		**4**	6.02	6.76				
Length ℓ	36	**46**	56	66				
Runtime [s]	47.5	**55.4**	60.3	66.9				
RF distance	4	**4**	4.02	4.84				
# patterns m	1	3	**5**	7				
Runtime [s]	13.5	34.4	**55.4**	75.94				
RF distance	4.12	4.02	**4**	4				
Threshold T	-50	-25	**0**	25	50	75	100	125
Runtime [s]	55.2	55.4	**55.4**	55.1	55.3	55.3	55.1	55.2
RF-Distance	11.92	12	**4**	12	12	12	12	12
			Wolbachia II					
Weight w	4	**6**	8	10				
Runtime [s]	112.4	**19.4**	18	17.8				
RF distance	20.38	**19.68**	22	22				
Length ℓ	36	**46**	56	66				
Runtime [s]	17	**19.4**	21.5	23.9				
RF distance	19.78	**19.68**	19.7	19.78				
# patterns m	1	3	**5**	7				
Runtime [s]	5.4	12.5	**19.4**	26.5				
RF distance	19.06	19.12	**19.68**	19.82				
Threshold T	-50	-25	**0**	25	50	75	100	125
Runtime [s]	19.6	19.6	**19.4**	19.4	19.4	19.4	19.4	19.4
RF-Distance	22.18	18	**19.68**	19.86	20.16	20.7	21.04	22

Table 4: Program runtime and *Robinson-Foulds* distances to reference trees for different parameter values with *Prot-SpaM* for the *E. coli/Shigella* and *Wolbachia* proteomes. We ran our program with different values for the *weight* w and length ℓ of the spaced-words, for different numbers of patterns and for different values of the threshold T. Here, we modified the value of one of these parameters at a time and used the default values for the remaining three parameters. Default values of the modified parameters and the resulting runtimes and *RF* distances are shown in bold font. Since *Prot-SpaM* uses a probabilistic algorithm to generate pattern sets, we performed 100 program runs for each set of parameters; the table reports the average *RF* distances of these 100 runs.

Plants

Weight w	4	**6**	8	10				
Runtime [s]	57,578	**464**	320	325				
RF distance	2	**0**	4	6				
Length ℓ	36	**46**	56	66				
Runtime [s]	383	**464**	441	494				
RF distance	2	**0**	0	0				
# patterns m	1	3	**5**	7				
Runtime [s]	91	255	**464**	572				
RF distance	0	0	**0**	2				
Threshold T	-50	-25	**0**	25	50	75	100	125
Runtime [s]	383	402	**464**	409	391	459	439	430
RF-Distance	2	2	**2**	0	0	0	2	4

Metazoa

Weight w		**6**	8	10				
Runtime [s]		**1719**	1584	1518				
RF distance		**30**	26	30				
Length ℓ	36	**46**	56	66				
Runtime [s]	1351	**1719**	1584	2089				
RF distance	26	**30**	24	26				
# patterns	1	3	**5**	7				
Runtime [s]	427	890	**1719**	2078				
RF distance	24	28	**30**	26				
Threshold T	-50	-25	**0**	25	50	75	100	125
Runtime [s]	2539	2337	**1719**	2269	2150	1906	1783	1797
RF-Distance	30	24	**30**	26	28	28	30	34

Table 5: Program runtime and *Robinson-Foulds* distances to reference trees for different parameter values with *Prot-SpaM* for the *plant* and *metazoan* proteomes; parameter values as in Table 4. Because of the size of these data sets, we performed only one program run per parameter set.

the whole-proteome sequences, but it was outperformed by word-frequency methods on the 252 selected orthologous proteins. A possible explanation of this result is discussed below. On the large prokaryote data set and for the metazoan set, by contrast, none of the compared programs could reproduce the reference trees that we used in our evaluation. These are difficult data sets since they span very large evolutionary distances; also it should be mentioned that there are no absolutely reliable reference trees available for these data sets. For the metazoan data set, for example, the positions of the ctenophores is still a matter of debate [15, 21, 51]. On the metazoans, *Prot-SpaM* performed better than other alignment-free approaches, while on the large prokaryote data set, *CVTree, ACS* and *kmacs* were superior.

~~Phylogenetic trees generated from these distance values are generally of high quality. Table 2 shows that, for various sets of taxa, trees based on *Prot-SpaM* distances, calculated from whole proteomes, are more similar to the respective reference trees than the trees that we obtained with alternative alignment-free methods. Interestingly, this result was reversed for the taxa set *Wolbachia* I, when we used only proteins from 252 selected genes instead of all available protein sequences.~~ An~~other~~ interesting result is the performance of *Prot-SpaM*, compared to our previous approach *FSWM* that takes genomic sequences as input. For most groups of taxa in our study, the results of *Prot-SpaM* and *FSWM* were of similar quality, in the sense that the *RF* distances to the reference trees were comparable for both approaches. However, for the set of 813 prokaryote taxa, our new spaced-words approach performed better on whole-proteomes than our previous approach on whole genomes, as is shown in Figure 6 and Table 2. This discrepancy is most likely due to the large phylogenetic distances in this data set; for such distantly related sequences, homologies are generally better detectable at the protein level than at the DNA level.

'Alignment-free' methods to phylogeny reconstruction can be directly applied to whole-genome or whole-proteome sequences, without the need to select orthologous genes or proteins in a first step. This is generally seen as an advantage over more traditional, alignment-based approaches, since the task of finding orthologs is time-consuming and often involves manual intervention. On our data set *Wolbachia I*, we actually obtained better *RF* distances with *Prot-SpaM* and *FSWM* when we applied these programs to the whole-protein or whole-genome sequences, respectively, than when we applied them to the 252 selected orthologous proteins or to the genes coding for those proteins, see Table 2. These results are in contrast to the more traditional alignment-free methods *FFP*, *CVtree* and *ACS* that are based on word-frequencies or on the length of common substrings. The

latter programs performed better on the selected orthologous proteins of the *Wolbachia I* data set than on the corresponding whole-proteome sequences.

A possible explanation of this phenomenon is that *Prot-SpaM* and *FSWM* can reliably distinguish between homologous and background spaced-word matches and use only homologous matches for phylogenetic inference. With the *one-to-one* spaced-word matching, they can also reduce the number of *paralogous* spaced-word matches. Therefore, they can be applied to whole proteomes or whole genomes without being too much confused by paralogs or by non-related parts of the sequences. Here, the benefits of using larger input sequence sets seem to outweigh the disadvantage of including possible non-related sequences, paralogs or sequences with recombinations. Previously introduced word-frequency or substring-length methods, by contrast, do not distinguish between homologous and non-homologous parts of the sequences. Therefore, these approaches tend to be confused by input sequences that contain paralogs or are only locally related to each other.

Table 3 shows that the run time of Prot-SpaM is superior to *CVtree* and *ACS* on protein sequences. By far the fastest alignment-free method on whole proteomes was *FFP*, the slowest one was *kmacs*. On the plant proteomes, *Prot-Spam* was three orders of magnitude faster than *FSWM* on the genome sequences of the same species. This is not surprising, given the fact that in eukaryotes only a small part of the genome is protein-coding sequence, so the proteomes are substantially smaller than the corresponding genomes.

Prot-SpaM has four major parameters that can be adjusted by the user: the weight w and the length ℓ of the patterns and spaced-words, respectively, the cut-off value T to distinguish homologous from random spaced-word matches and the number m of different patterns used to generate spaced-word matches. We provide default values for these parameters, but Tables 4 and 5 show that reasonable results can be obtained with a rather broad range of parameter values. These tables also show that the quality of the produced trees, as measured by the *RF* distances to the reference trees, could not be improved by using $m = 5$ patterns, compared to the single-pattern option, *i.e.* $m = 1$. The statistical stability of our distance estimates, however, is increased if multiple patterns are used; therefore, we are using $m = 5$ patterns by default. But since runtime and memory usage of our program increase with the number m of pattern, it may be advisable to use the single-pattern option if very large data sets are to be analyzed.

It should be mentioned that traditional approaches to phylogeny reconstruction that are based on multiple sequence alignment are still more

accurate than alignment-free approaches that have been proposed in recent years. The main advantage of these novel approaches is their high speed, with makes it possible to apply them to the large sequence data sets that are now available; a program run of *Prot-SpaM* on whole-proteome sequences of the set *Wolbachia II* that consists of 47 taxa, took only 19 seconds. Another advantage of our approach is that it can reliably distinguish between local homologies and random background similarities. It can, thus, be applied to complete or incomplete proteomes, and it is not necessary to select orthologous genes or proteins in a first step. ~~For the taxa set *Wolbachia* I, we obtained better results when we used all available protein sequences from GenBank, than with the proteins corresponding to 252 carefully selected genes.~~ Therefore, we think that *Prot-SpaM* should be a useful addition to existing approaches to phylogeny reconstruction.

Acknowledgements

We thank Jonathan Eisen for his comments on the phylogeny of the 841 microbial taxa that were studied in Lang *et al.* Five reviewers made very helpful comments on an earlier version of this manuscript. We acknowledge support by the *Open Access Publication Funds* of the *Göttingen University*.

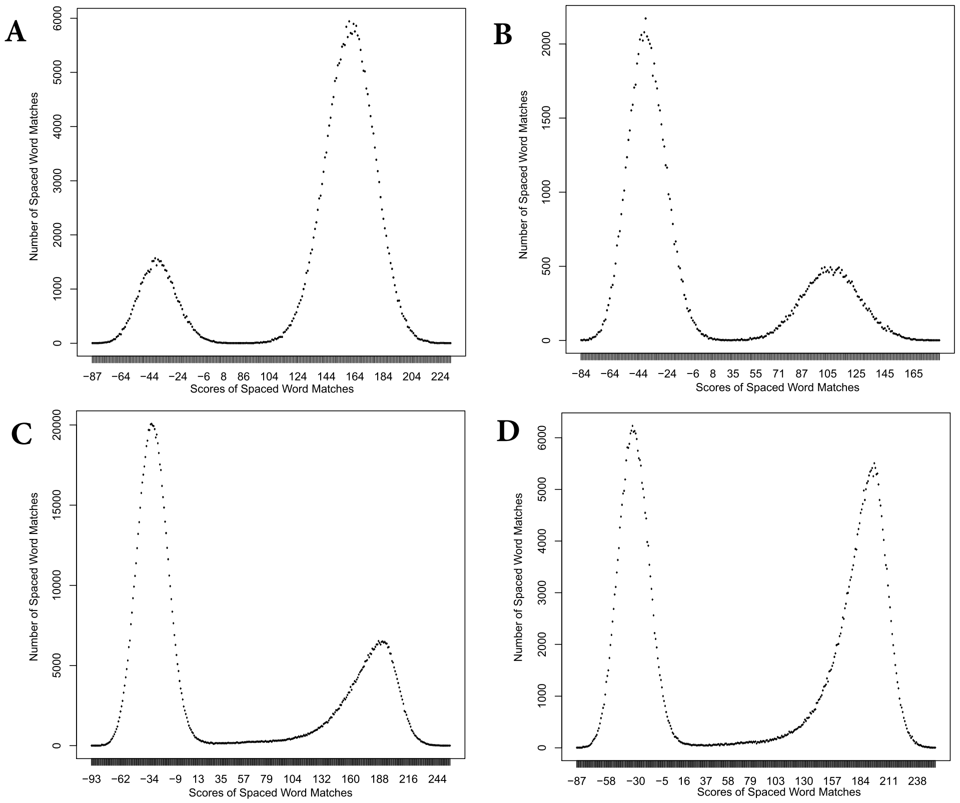

Figure 1: *Spaced-word histograms ('spamograms')* for different data sets. (A) and (B) are based on simulated indel-free protein sequences with a total length of of 1.6×10^6 amino-acid residues each, and with 0.3 (A) and 0.75 (B) substitutions per position, respectively. (C) and (D) are from a whole-proteome comparisons of plants, (C) comparing *Eucalyptus grandis* with *Capsella rubella* and (D) comparing *Gossypium raimondii* with *Carica papaya*.

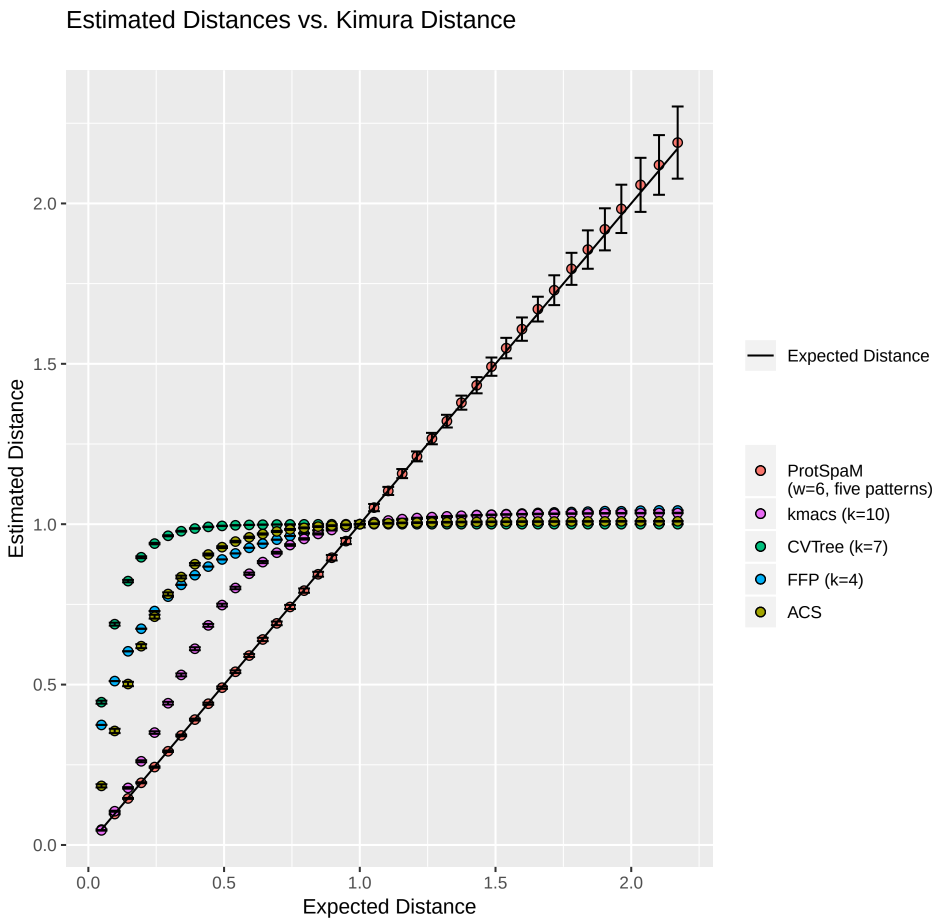

Figure 2: Distances calculated by *Prot-SpaM* and four other alignment-free methods calculated for pairs of simulated protein sequences, plotted against their distances calculated with the *Kimura* model. Error bars denote standard deviations. Note that *Prot-SpaM* estimates phylogenetic distances in terms of substitutions that have happened since two sequences evolved from their lasts common ancestor. The programs *kmacs, CVTree, FFP* and *ACS*, by contrast, do not estimate distances in a rigorous way, but rather use ad-hoc measures of sequence dissimilarity that are not linear functions of the real distances. Also, the absolute values of these distance measures are rather arbitrary for these four other programs. We therefore normalized the distances calculated by *kmacs, CVTree, FFP* and *ACS* such that they have a value of one for sequence pairs with a *Kimura* distance of one.

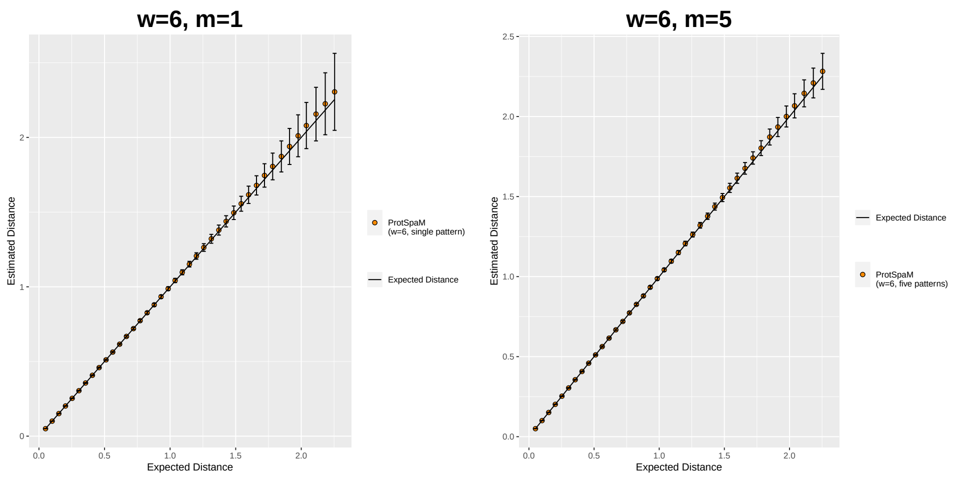

Figure 3: Distances calculated by *Prot-SpaM* for pairs of simulated protein sequences with a single binary pattern ($m = 1$, left) and with the default multiple-pattern option ($m = 5$, right). We performed 1000 program runs for each value of m. The plot shows the *average* of the calculated distances; *standard deviations* are shown as error bars.

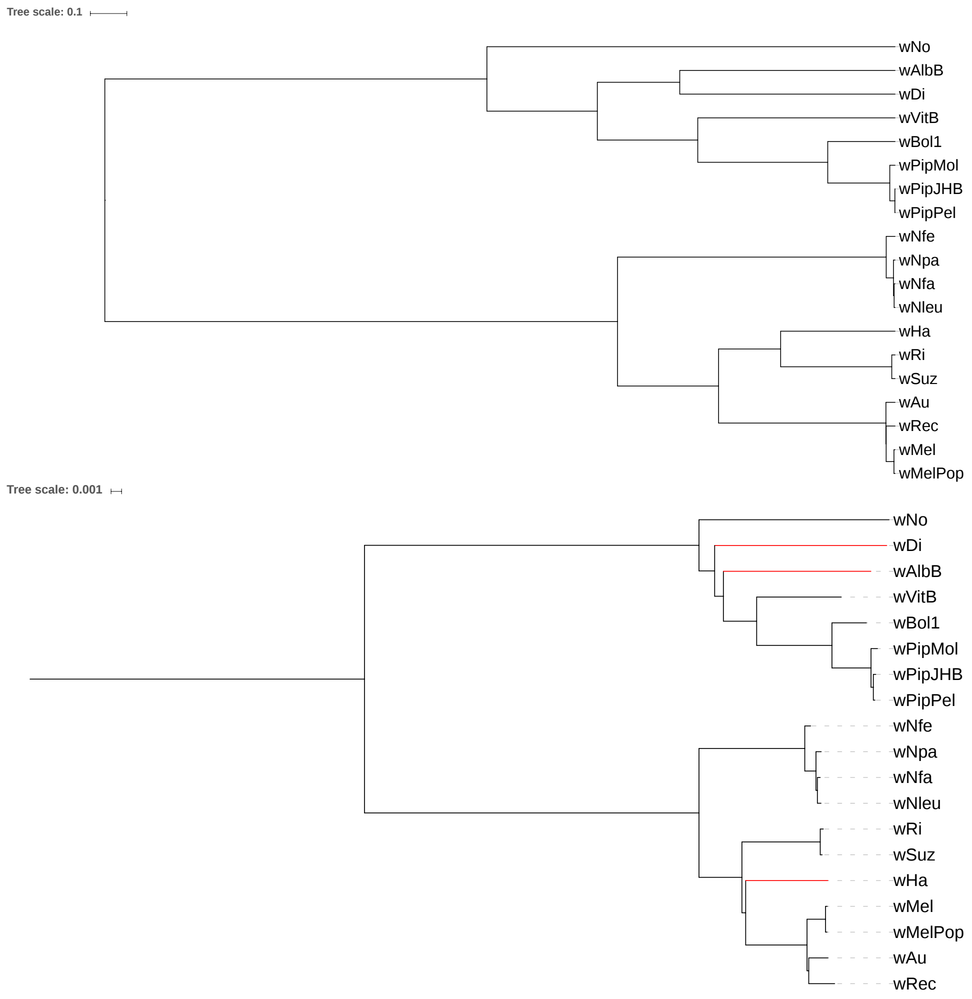

Figure 4: Reference tree for our data set *Wolbachia I* (above) and tree calculated with *Prot-SpaM* using whole-proteome sequences of the same taxa (below), see main text for details. Topological differences between the two trees are shown in red in the *Prot-SpaM* tree.

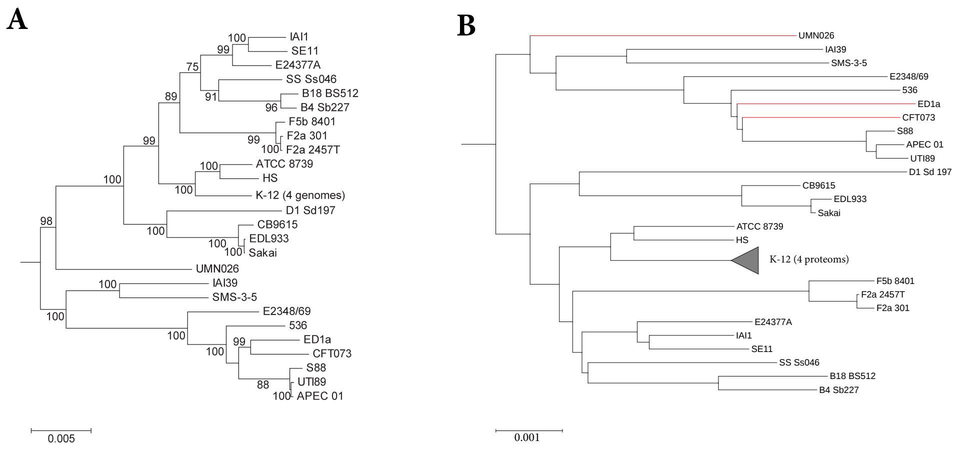

Figure 5: Reference tree (A) from [74] and tree calculated with *Prot-SpaM* with default parameters (B) for a set of 29 *Escherichia coli* and *Shigella* strains. Differences in the topologies between the two trees are marked in red.

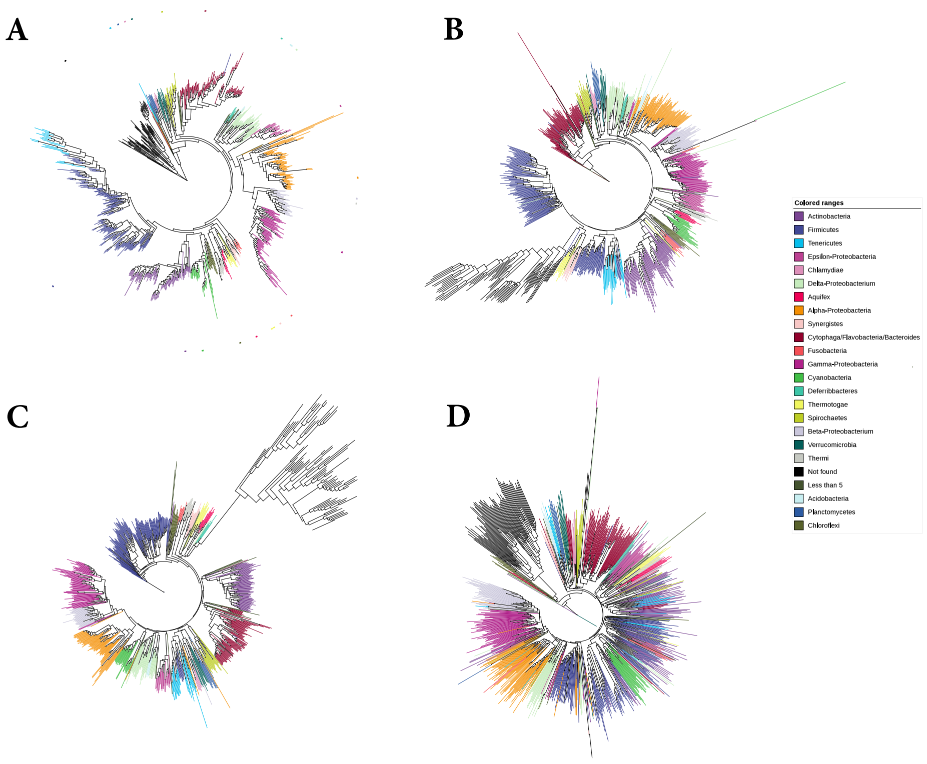

Figure 6: Phylogenetic trees for a large set of microbial taxa studied by Lang *et al.* [40]. (A) Maximum-Likelihood tree constructed by Lang *et al.* based on a super alignment of 24 selected genes, (B) tree constructed with our approach, as described in this paper, for 813 taxa for which the proteomes are available in *GenBank*, (C) tree constructed with our approach based on the proteins corresponding to the 24 genes selected by Lang *et al.* and (D) tree reconstructed using our program *FSWM* [44] on the 841 whole-genome sequences.

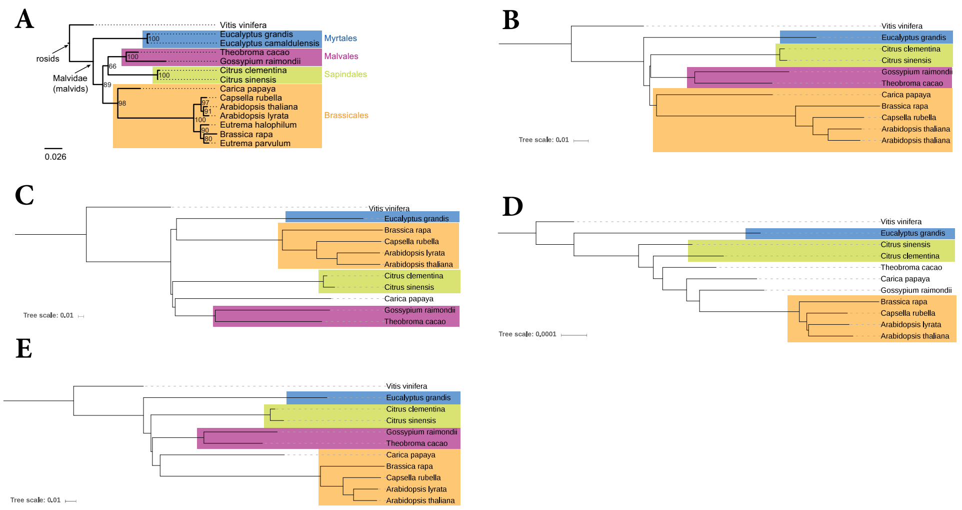

Figure 7: Phylogenetic trees of plant taxa. (A) reference tree from [26], and trees constructed with (B) the approach described in this paper, (C) *ACS* [68], (D) *FFP* [59], and (E) *kmacs* [43]. The original data set contained 14 taxa, but only for 11 taxa, the proteomes could be downloaded through *GenBank*. For completeness, we show the reference for all 14 taxa.

References

[1] Dennis A Benson, Mark Cavanaugh, Karen Clark, Ilene Karsch-Mizrachi, James Ostell, Kim D Pruitt, and Eric W Sayers. Genbank. *Nucleic Acids Research*, 46(D1):D41–D47, 2018.

[2] Guillaume Bernard, Cheong Xin Chan, Yao-ban Chan, Xin-Yi Chua, Yingnan Cong, James M. Hogan, Stefan R. Maetschke, and Mark A. Ragan. Alignment-free inference of hierarchical and reticulate phylogenomic relationships. *Briefings in Bioinformatics*, in press:bbx067, 2017.

[3] John Besemer and Mark Borodovsky. GeneMark: web software for gene finding in prokaryotes, eukaryotes and viruses. *Nucleic Acids Research*, 33:W451–W454, 2005.

[4] Olaf R.P. Bininda-Emonds. The evolution of supertrees. *Trends in Ecology and Evolution*, 19:315 – 322, 2004.

[5] Seth R. Bordenstein, Charalampos Paraskevopoulos, Julie C. Dunning Hotopp, Panagiotis Sapountzis, Nathan Lo, Claudio Bandi, Herv Tettelin, John H. Werren, and Kostas Bourtzis. Parasitism and mutualism in Wolbachia: What the phylogenomic trees can and cannot say. *Molecular Biology and Evolution*, 26:231–241, 2009.

[6] Marek L. Borowiec, Ernest K. Lee, Joanna C. Chiu, and David C. Plachetzki. Extracting phylogenetic signal and accounting for bias in whole-genome data sets supports the Ctenophora as sister to remaining Metazoa. *BMC Genomics*, 16:987, 2015.

[7] Amanda M. V. Brown, Sulochana K. Wasala, Dana K. Howe, Amy B. Peetz, Inga A. Zasada, and Dee R. Denver. Genomic evidence for plant-parasitic nematodes as the earliest Wolbachia hosts. *Scientific Reports*, 6:34955, 2016.

[8] Trevor C. Bruen, Hervé Philippe, and David Bryant. A simple and robust statistical test for detecting the presence of recombination. *Genetics*, 172:2665–2681, 2006.

[9] David Bryant and Mike Steel. Computing the distribution of a tree metric. *IEEE/ACM Trans. Comput. Biol. Bioinformatics*, 6:420–426, 2009.

[10] Olga Chernomor, Arndt von Haeseler, and Bui Quang Minh. Terrace aware data structure for phylogenomic inference from supermatrices. *Systematic Biology*, 65:997–1008, 2016.

[11] Benny Chor, David Horn, Yaron Levy, Nick Goldman, and Tim Massingham. Genomic DNA k-mer spectra: models and modalities. *Genome Biology*, 10:R108, 2009.

[12] Matteo Comin and Davide Verzotto. Alignment-free phylogeny of whole genomes using underlying subwords. *Algorithms for Molecular Biology*, 7:34, 2012.

[13] Margaret O. Dayhoff, Robert M. Schwartz, and Bruce C. Orcutt. A model of evolutionary change in proteins. *Atlas of Protein Sequence and Structure*, 6:345–362, 1978.

[14] Thomas Dencker, Chris-André Leimeister, and Burkhard Morgenstern. *Multi-SpaM*: a maximum-likelihood approach to phylogeny reconstruction based on multiple spaced-word matches. *arXiv*, 1803.09222[q-bio.PE], 2018.

[15] Casey W. Dunn, Gonzalo Giribet, Gregory D. Edgecombe, and Andreas Hejnol. Animal phylogeny and its evolutionary implications. *Annual Review of Ecology, Evolution, and Systematics*, 45:371–395, 2014.

[16] Sean R. Eddy. A new generation of homology search tools based on probabilistic inference. In *Genome Informatics 2009 - Proceedings of the 20th International Conference*, pages 205–211. Imperial College Press, 2009.

[17] David M. Emms and Steven Kelly. OrthoFinder: solving fundamental biases in whole genome comparisons dramatically improves orthogroup inference accuracy. *Genome Biology*, 16:157, 2015.

[18] Huan Fan, Anthony R. Ives, Yann Surget-Groba, and Charles H. Cannon. An assembly and alignment-free method of phylogeny reconstruction from next-generation sequencing data. *BMC Genomics*, 16:522, 2015.

[19] Joseph Felsenstein. PHYLIP - Phylogeny Inference Package (Version 3.2). *Cladistics*, 5:164–166, 1989.

[20] Umberto Ferraro-Petrillo, Gianluca Roscigno, Giuseppe Cattaneo, and Raffaele Giancarlo. Informational and linguistic analysis of large genomic sequence collections via efficient hadoop cluster algorithms. *Bioinformatics*, 34:18261833, 2018.

[21] Roberto Feuda, Martin Dohrmann, Walker Pett, Herv Philippe, Omar Rota-Stabelli, Nicolas Lartillot, Gert Wrheide, and Davide Pisani. Improved modeling of compositional heterogeneity supports sponges as sister to all other animals. *Current Biology*, 27:3864 – 3870.e4, 2017.

[22] Michael Gerth and Christoph Bleidorn. Comparative genomics provides a timeframe for *Wolbachia* evolution and exposes a recent biotin synthesis operon transfer. *Nature Microbiology*, 2:16241, 2016.

[23] Michael Gerth, Marie-Theres Gansauge, Anne Weigert, and Christoph Bleidorn. Phylogenomic analyses uncover origin and spread of the Wolbachia pandemic. *Nature Communications*, 5:5117, 2014.

[24] Eliza Glowska, Anna Dragun-Damian, Miroslawa Dabert, and Michael Gerth. New Wolbachia supergroups detected in quill mites (Acari: Syringophilidae). *Infection, Genetics and Evolution*, 30:140–146, 2015.

[25] Lars Hahn, Chris-André Leimeister, Rachid Ounit, Stefano Lonardi, and Burkhard Morgenstern. *rasbhari*: optimizing spaced seeds for database searching, read mapping and alignment-free sequence comparison. *PLOS Computational Biology*, 12(10):e1005107, 2016.

[26] Klas Hatje and Martin Kollmar. A phylogenetic analysis of the brassicales clade based on an alignment-free sequence comparison method. *Frontiers in Plant Science*, 3:192, 2012.

[27] Bernhard Haubold. Alignment-free phylogenetics and population genetics. *Briefings in Bioinformatics*, 15:407–418, 2014.

[28] Bernhard Haubold, Fabian Klötzl, and Peter Pfaffelhuber. andi: Fast and accurate estimation of evolutionary distances between closely related genomes. *Bioinformatics*, 31:1169–1175, 2015.

[29] Bernhard Haubold, Peter Pfaffelhuber, Mirjana Domazet-Loso, and Thomas Wiehe. Estimating mutation distances from unaligned genomes. *Journal of Computational Biology*, 16:1487–1500, 2009.

[30] Steven Henikoff and Jora G. Henikoff. Amino acid substitution matrices from protein blocks. *Proc. Natl. Acad. Sci. USA*, 89:10915–10919, 1992.

[31] Michael Höhl, Isidore Rigoutsos, and Mark A. Ragan. Pattern-based phylogenetic distance estimation and tree reconstruction. *Evolutionary Bioinformatics Online*, 2:359–375, 2006.

[32] Sebastian Horwege, Sebastian Lindner, Marcus Boden, Klaus Hatje, Martin Kollmar, Chris-André Leimeister, and Burkhard Morgenstern. *Spaced words* and *kmacs*: fast alignment-free sequence comparison based on inexact word matches. *Nucleic Acids Research*, 42:W7–W11, 2014.

[33] Lucian Ilie, Silvana Ilie, and Anahita M. Bigvand. SpEED: fast computation of sensitive spaced seeds. *Bioinformatics*, 27:2433–2434, 2011.

[34] David T. Jones, William R. Taylor, and Janet M. Thornton. The rapid generation of mutation data matrices from protein sequences. *Bioinformatics*, 8(3):275–282, 1992.

[35] Se-Ran Jun, Gregory E. Sims, Guohong A. Wu, and Sung-Hou Kim. Whole-proteome phylogeny of prokaryotes by feature frequency profiles: An alignment-free method with optimal feature resolution. *Proceedings of the National Academy of Sciences*, 107:133–138, 2010.

[36] Subha Kalyaanamoorthy, Minh Q. Bui, Thomas K F Wong, Arndt von Haeseler, and Lars S. Jermiin. ModelFinder: fast model selection for accurate phylogenetic estimates. *Nature Methods*, 14:587–589, 2017.

[37] Kazutaka Katoh and Daron M. Standley. MAFFT multiple sequence alignment software version 7: Improvements in performance and usability. *Molecular Biology and Evolution*, 30:772–780, 2013.

[38] Motoo Kimura. *The Neutral Theory of Molecular Evolution.* Cambridge University Press, 1983.

[39] Patrick Kück and Gary C. Longo. FASconCAT-G: extensive functions for multiple sequence alignment preparations concerning phylogenetic studies. *Frontiers in Zoology*, 11:81, 2014.

[40] Jenna Morgan Lang, Aaron E. Darling, and Jonathan A. Eisen. Phylogeny of bacterial and archaeal genomes using conserved genes: Supertrees and supermatrices. *PLOS ONE*, 8:e62510, 2013.

[41] Bret R. Larget, Satish K. Kotha, Colin N. Dewey, and Cécile Ané. BUCKy: Gene tree/species tree reconciliation with Bayesian concordance analysis. *Bioinformatics*, 26:2910–2911, 2010.

[42] Chris-André Leimeister, Marcus Boden, Sebastian Horwege, Sebastian Lindner, and Burkhard Morgenstern. Fast alignment-free sequence comparison using spaced-word frequencies. *Bioinformatics*, 30:1991–1999, 2014.

[43] Chris-André Leimeister and Burkhard Morgenstern. *kmacs*: the k-mismatch average common substring approach to alignment-free sequence comparison. *Bioinformatics*, 30:2000–2008, 2014.

[44] Chris-André Leimeister, Salma Sohrabi-Jahromi, and Burkhard Morgenstern. Fast and accurate phylogeny reconstruction using filtered spaced-word matches. *Bioinformatics*, 33:971–979, 2017.

[45] Ivica Letunic and Peer Bork. Interactive tree of life (iTOL) v3: an online tool for the display and annotation of phylogenetic and other trees. *Nucleic Acids Research*, 44:W242–W245, 2016.

[46] Liang Liu, Zhenxiang Xi, Shaoyuan Wu, Charles C. Davis, and Scott V. Edwards. Estimating phylogenetic trees from genome-scale data. *Annals of the New York Academy of Sciences*, 1360:36–53, 2015.

[47] Burkhard Morgenstern, Svenja Schöbel, and Chris-André Leimeister. Phylogeny reconstruction based on the length distribution of k-mismatch common substrings. *Algorithms for Molecular Biology*, 12:27, 2017.

[48] Burkhard Morgenstern, Bingyao Zhu, Sebastian Horwege, and Chris-André Leimeister. Estimating evolutionary distances between genomic sequences from spaced-word matches. *Algorithms for Molecular Biology*, 10:5, 2015.

[49] Lam-Tung Nguyen, Heiko A. Schmidt, Arndt von Haeseler, and Bui Quang Minh. IQ-TREE: A fast and effective stochastic algorithm for estimating maximum-likelihood phylogenies. *Molecular Biology and Evolution*, 32:268–274, 2015.

[50] Laurent Noé. Best hits of 11110110111: model-free selection and parameter-free sensitivity calculation of spaced seeds. *Algorithms for Molecular Biology*, 12:1, 2017.

[51] Hervé Philippe, R Derelle, P Lopez, Kerstin Pick, C Borchiellini, N Boury-Esnault, J Vacelet, E Renard, E. Houliston, E. Quéinnec,

C. Da Silva, P. Wincker, H. Le Guyader, S. Leys, Daniel Jackson, Fabian Schreiber, Dirk Erpenbeck, Burkhard Morgenstern, Gert Wörheide, and M Manuel. Phylogenomics restores traditional views on deep animal relationships. *Current Biology*, 19:706–712, 2009.

[52] Cinzia Pizzi. MissMax: alignment-free sequence comparison with mismatches through filtering and heuristics. *Algorithms for Molecular Biology*, 11:6, 2016.

[53] Ji Qi, Hong Luo, and Bailin Hao. CVTree: a phylogenetic tree reconstruction tool based on whole genomes. *Nucleic Acids Research*, 32(suppl 2):W45–W47, 2004.

[54] Gesine Reinert, David Chew, Fengzhu Sun, and Michael S. Waterman. Alignment-free sequence comparison (I): Statistics and power. *Journal of Computational Biology*, 16:1615–1634, 2009.

[55] Jie Ren, Xin Bai, Yang Young Lu, Kujin Tang, Ying Wang, Gesine Reinert, and Fengzhu Sun. Alignment-free sequence analysis and applications. *Annual Review of Biomedical Data Science*, 1:93–114, 2018.

[56] David F Robinson and Les Foulds. Comparison of phylogenetic trees. *Mathematical Biosciences*, 53:131–147, 1981.

[57] Fredrik Ronquist and John P. Huelsenbeck. MrBayes 3: Bayesian phylogenetic inference under mixed models. *Bioinformatics*, 19:1572–1574, 2003.

[58] Naruya Saitou and Masatoshi Nei. The neighbor-joining method: a new method for reconstructing phylogenetic trees. *Molecular Biology and Evolution*, 4:406–425, 1987.

[59] Gregory E. Sims, Se-Ran Jun, Guohong A. Wu, and Sung-Hou Kim. Alignment-free genome comparison with feature frequency profiles (FFP) and optimal resolutions. *Proceedings of the National Academy of Sciences*, 106:2677–2682, 2009.

[60] Kai Song, Jie Ren, Zhiyuan Zhai, Xuemei Liu, Minghua Deng, and Fengzhu Sun. Alignment-free sequence comparison based on next-generation sequencing reads. *Journal of Computational Biology*, 20:64–79, 2013.

[61] Stephanie J. Spielman and Claus O. Wilke. Pyvolve: A flexible python module for simulating sequences along phylogenies. *PLOS ONE*, 10(9):e0139047, 2015.

[62] Alexandros Stamatakis. RAxML-VI-HPC: maximum likelihood-based phylogenetic analyses with thousands of taxa and mixed models. *Bioinformatics*, 22:2688–2690, 2006.

[63] Alexandros Stamatakis. RAxML version 8: a tool for phylogenetic analysis and post-analysis of large phylogenies. *Bioinformatics*, 30:1312–1313, 2014.

[64] Mike Steel. Phylogenetic diversity and the greedy algorithm. *Systematic Biology*, 54:527–529, 2005.

[65] Hanno Teeling, Jost Waldmann, Thierry Lombardot, Margarete Bauer, and Frank Oliver Glöckner. Tetra: a web-service and a stand-alone program for the analysis and comparison of tetranucleotide usage patterns in dna sequences. *BMC Bioinformatics*, 5:163, 2004.

[66] Sharma V. Thankachan, Sriram P. Chockalingam, Yongchao Liu, and Ambujam Krishnan Srinivas Aluru. A greedy alignment-free distance estimator for phylogenetic inference. *BMC Bioinformatics*, 18:238, 2017.

[67] Sharma V. Thankachan, Sriram P. Chockalingam, Yongchao Liu, Alberto Apostolico, and Srinivas Aluru. ALFRED: a practical method for alignment-free distance computation. *Journal of Computational Biology*, 23:452–460, 2016.

[68] Igor Ulitsky, David Burstein, Tamir Tuller, and Benny Chor. The average common substring approach to phylogenomic reconstruction. *Journal of Computational Biology*, 13:336–350, 2006.

[69] Susana Vinga, Alexandra M. Carvalho, Alexandre P. Francisco, Luís M. S. Russo, and Jonas S. Almeida. Pattern matching through Chaos Game Representation: bridging numerical and discrete data structures for biological sequence analysis. *Algorithms for Molecular Biology*, 7:10, 2012.

[70] Lin Wan, Gesine Reinert, Fengzhu Sun, and Michael S Waterman. Alignment-free sequence comparison (II): theoretical power of comparison statistics. *Journal of Computational Biology*, 17:1467–1490, 2010.

[71] John H. Werren, Laura Baldo, and Michael E. Clark. Wolbachia: master manipulators of invertebrate biology. *Nature Reviews Microbiology*, 6:741 – 751, 2008.

[72] Huiguang Yi and Li Jin. Co-phylog: an assembly-free phylogenomic approach for closely related organisms. *Nucleic Acids Research*, 41:e75, 2013.

[73] Xiaofan Zhou, Xing-Xing Shen, Chris Todd Hittinger, and Antonis Rokas. Evaluating fast maximum likelihood-based phylogenetic programs using empirical phylogenomic data sets. *Molecular Biology and Evolution*, 35:486–503, 2018.

[74] Zhemin Zhou, Xiaomin Li, Bin Liu, Lothar Beutin, Jianguo Xu, Yan Ren, Lu Feng, Ruiting Lan, Peter R. Reeves, and Lei Wang. Derivation of *Escherichia coli* O157:H7 from Its O55:H7 Precursor. *PLOS ONE*, 5:e8700, 2010.

[75] Andrzej Zielezinski, Susana Vinga, Jonas Almeida, and Wojciech M. Karlowski. Alignment-free sequence comparison: benefits, applications, and tools. *Genome Biology*, 18:186, 2017.

Click here to access/download
Supplementary Material
protspam_supp_mat.pdf
