# Supplementary material for: Prot-SpaM: fast alignment-free phylogeny reconstruction based on whole-proteome sequences
Source: Gigascience. 2018 Dec 7;8(3):giy148. doi: 10.1093/gigascience/giy148 (PMC6436989; doi:10.1093/gigascience/giy148)
Supplement: GIGA-D-18-00169_R2.pdf [file giy148_giga-d-18-00169_r2.pdf]

GigaScience

Prot-SpaM: Fast alignment-free phylogeny reconstruction based on whole-proteome sequences

--Manuscript Draft--

**Manuscript Number:**	GIGA-D-18-00169R2
**Full Title:**	Prot-SpaM: Fast alignment-free phylogeny reconstruction based on whole-proteome sequences
**Article Type:**	Research
**Funding Information:**	
**Abstract:**	Word-based or ‘alignment-free’ sequence comparison has become an active research area in bioinformatics. While previous word-frequency approaches calculated rough measures of sequence similarity or dis-similarity, some new alignment-free methods are able to accurately estimate phylogenetic distances between genomic sequences. One of these approaches is Filtered Spaced Word Matches. Herein, we ex-tend this approach to estimate evolutionary distances between com-plete or incomplete proteomes; our implementation of this approach is called Prot-SpaM. We compare the performance of Prot-SpaM to other alignment-free methods on simulated sequences and on various groups of eukaryotic and prokaryotic taxa. Prot-SpaM can be used to calculate high-quality phylogenetic trees for dozens of whole-proteome sequences in a matter of seconds or minutes and often outperforms other alignment-free approaches. The source code of our software is available through Github: https://github.com/jschellh/ProtSpaM
**Corresponding Author:**	Chris-Andre Leimeister<br><br>GERMANY
**Corresponding Author Secondary Information:**	
**Corresponding Author's Institution:**	
**Corresponding Author's Secondary Institution:**	
**First Author:**	Chris-Andre Leimeister
**First Author Secondary Information:**	
**Order of Authors:**	Chris-Andre Leimeister
	Jendrik Schellhorn
	Svenja Schöbel
	Michael Gerth
	Christoph Bleidorn
	Burkhard Morgenstern
**Order of Authors Secondary Information:**	
**Response to Reviewers:**	Reviewer #3:<br><br>REVIEWER:<br>-. In the manuscript, "In addition, we ran Prot-SpaM on the protein sequences encoded by the 24 marker genes from Lang et al". However, no discussion was found.<br><br>REPLY:<br>This paper is on a new alignment-free approach, not on the phylogeny of prokaryotes. Therefore, we restricted ourselves in this example to compare the phylogenies

Powered by Editorial Manager® and ProduXion Manager® from Aries Systems Corporation

produced by various alignment-free programs to a published reference phylogeny.

REVIEWER:
-. In discussion section, authors claim that "Another advantage of our approach is that it can reliably distinguish between local homologies and random background similarities. It can, thus, be applied to complete or incomplete proteomes" in comparison with alignment-based methods. I don't understand this sentence and how alignment-based approaches are lacking this advantage.

REPLY:
As we said: for alignment-based approach, orthologous genes/proteins must be selected first. With our "filtering" approach, we automatically and rather reliably discard non-homologous spaced-word matches, so it is not necessary to select orthologous genes/proteins in a first step. We think that this is an advantage. But this is the "Discussion" section and, of course, one can have different opinions on this point.

REVIEWER:
-. In response to the comment, Authors noted that K=4 was used for FFP method for proteome datasets. First of all, authors should include this information like authors noted K=10 for Kmacs whenever FFP was compared with Prot-SpaM because K-mer length is a key to FFP method. However, this information is missing in tables and figures except for Figure 2. Second, Jun et al's paper (PANS, 2010; 107:133-138) has discussed about the optimal K-mer length for FFP method for whole proteome dataset which is something near K=10~K=13, which is very different from the one used in the manuscript for comparison. Basically, authors picked up K for FFP far from optimal and compared it with Prot-SpaM. I guess that this might result in large RF scores for FFP method.

REPLY:
In the manuscript, we clarify that all competing programs were run with their default parameters. This is common practice if software programs are evaluated. In addition, we explicitly mention that, for FFP, this means k=4. We now added this information to Tables 2 and 3 for clarity.

REVIEWER:
-. For plants, authors noted that a set of plant taxa and a reference tree from the reference [26] were used. Since there are many trees shown in the reference [26], authors should describe which tree they used as the reference tree. Also, authors described they used only partial set of plant taxa because some plants don't have proteomes deposited into GenBank. So, authors should describe how they rebuilt a reference tree for the partial set to calculate RF scores since they cannot use the reference tree directly from the reference tree [26].

REPLY:
We clarified this in the manuscript (we used the tree B from Figure 3 from [26] and removed the three species for which we could not obtain the proteomes from GenBank).

REVIEWER:
-. In response to the comment, Authors noted that "Alignment-free approaches are certainly computationally more efficient than 16S rRNA-based approaches where one first has to identify these sequences and then construct an alignment". This is not correct. Unlike Prot-SpaM which uses only space-word matches to calculate (dis)similarity between whole proteomes, other alignment free methods mentioned in the manuscript concern all information encoded in whole proteomes instead of trying to look at matched proteins/regions so that they keep all features present in whole proteomes to measure (dis)similarity between whole proteomes which is time consuming.

REPLY:
Yes, other alignment-free methods use information from the entire sequences (e.g. all k-mers or common substrings). Prot-SpaM, by contrast, restricts itself to spaced-word matches with positive score that can assumed to represent homologies. We think that

Powered by Editorial Manager® and ProduXion Manager® from Aries Systems Corporation

this is an advantage compared to other approaches. We discuss this point in the "Discussion" but, again, one may have different views on this point. Apart from this point, we do not understand how this is related with 16S rRNA. And we still think, that alignment-free approaches are faster than alignment-based approaches with 16S RNA.

REVIEWER:
-. In response to the comment, Authors said that "We do this by comparing phylogenetic trees to reference trees, and this is what all papers on alignment-free methods are doing to evaluate new tools.". Yes, people compare the outputs (here trees) to validate new tools. But, authors compared methods indirectly through the reference trees to validate their method which means that reference trees used are one of major factors in validation steps. However, authors used different genomic features for different datasets for reference trees. For example, Ecoli/Shigella dataset used 2,034 core genes, Wolbachia dataset used 252 core genes, whole proteome dataset used 24 core genes, Metazoa 1080 core genes for reference trees. Prot-SpaM uses only space-word matches which could be similar to core genes so that I speculate could make core genes-based reference trees more similar to the ones generated by Prot-SpaM. Anyway, I believe that people validate new tools for phylogeny construction in the following way: use 16S rRNA a universal marker for Prokaryotes, and compare trees built by new tools to trees of 16S rRNA for datasets of interest (which are reference trees) in terms of RF score as authors already did. I believe that alignment free methods considered in the manuscript all compared their trees to 16S rRNA trees for prokaryotes.

REPLY:
We agree with the reviewer: evaluating phylogeny methods by comparing their results to reference trees can be problematic, since the reference trees may not be the "correct" trees. This has to be taken into account if such benchmarking results are analyzed. As reference trees, we used trees published by experts in the literature, based on carefully selected marker genes. While we cannot be 100% sure that these trees are correct, we think that these published trees are certainly of higher quality than trees based on 16S RNA only. So it is quite natural that different marker genes were used, this has been done by experts on the respective taxa.

Reviewer #4: The authors have addressed all my concerns and I find the updates to the paper satisfactory.

Reviewer #5: In the revised version of the manuscript, the authors addressed most of my criticisms, either by undertaking the respective changes or by providing a justification for not doing so.

I have only a few minor suggestions/remarks:

- P7L29: "All test runs were done on a 10 x Intel(R) Xeon(R) CPU E7-4850 with 2.00GHz with 4 cores each summing up
to 40 cores and 1000GB RAM." Does it mean that inter-node parallelization was used for some programs? How this was achieved (MPI?). Please clarify.

REPLY:
This is not a cluster, it is one single server, so no internode communication was necessary. We used OpenMP for parallelization of Prot-SpaM and FSWM.

REVIEWER:
- P8L53: "The other alignment-free methods led to phylogenies with RF distances to the reference
tree between 22 and 44" - those numbers differ from Table 2, please double-check.

REPLY:
We corrected this.

REVIEWER:
- Table 2: please also double-check numbers in this table, e.g RF and rRF values for

	"Wolbachia I, whole proteomes"/FSWM appear to be inconsistent<br><br>REPLY:<br>We corrected this.<br><br>REVIEWER:<br>- Table 3: please indicate which programs where run in multi-threading mode (40 threads vs. 1 threads would make quite a difference!)<br><br>REPLY:<br>done<br><br>REVIEWER:<br>- P19L33: "This is not surprising, given the fact that in eukaryotes only a small part of the genome is protein-coding<br>sequence, so the proteomes are substantially smaller than the corresponding genomes." - could you please give proteome vs. genome size for this dataset to support this statement?<br><br>REPLY:<br>done<br><br>- Table 4 and 5: it seems that careful choice of parameter values can lead to substantial accuracy gains (which might be even higher if "favorable" values for several parameters are combined). Thus I still think that automatic parameter tuning based on dataset properties (spamograms, evolutionary distances) could be a promising future work direction.<br><br>REPLY:<br>Yes, we fully agree. But as the reviewer says, this will be "future work".
**Additional Information:**	
**Question**	**Response**
Are you submitting this manuscript to a special series or article collection?	No
**Experimental design and statistics**<br><br>Full details of the experimental design and statistical methods used should be given in the Methods section, as detailed in our Minimum Standards Reporting Checklist. Information essential to interpreting the data presented should be made available in the figure legends.<br><br>Have you included all the information requested in your manuscript?	Yes
**Resources**<br><br>A description of all resources used, including antibodies, cell lines, animals and software tools, with enough information to allow them to be uniquely	Yes

identified, should be included in the Methods section. Authors are strongly encouraged to cite Research Resource Identifiers (RRIDs) for antibodies, model organisms and tools, where possible.<br><br>Have you included the information requested as detailed in our Minimum Standards Reporting Checklist?	
**Availability of data and materials**<br><br>All datasets and code on which the conclusions of the paper rely must be either included in your submission or deposited in publicly available repositories (where available and ethically appropriate), referencing such data using a unique identifier in the references and in the “Availability of Data and Materials” section of your manuscript.<br><br>Have you have met the above requirement as detailed in our Minimum Standards Reporting Checklist?	Yes

Manuscript
Click here to access/download;Manuscript;protspam_rev2.pdf
Click here to view linked References

Prot-SpaM: Fast alignment-free phylogeny reconstruction based on whole-proteome sequences

Chris-Andre Leimeister[1,*], Jendrik Schellhorn[1,*], Svenja Schöbel[1], Michael Gerth[2], Christoph Bleidorn[3,4], and Burkhard Morgenstern[1,5]

[1]University of Göttingen, Department of Bioinformatics, Goldschmidtstr. 1, 37077 Göttingen, Germany
[2]Institute for Integrative Biology, University of Liverpool, Biosciences Building, Crown Street, L69 7ZB Liverpool, UK
[3]University of Göttingen, Department of Animal Evolution and Biodiversity, Untere Karspüle 2, 37073 Göttingen, Germany
[4]Museo Nacional de Ciencias Naturales, Spanish National Research Council (CSIC), 28006 Madrid, Spain
[5]Göttingen Center of Molecular Biosciences (GZMB), Justus-von-Liebig-Weg 11, 37077 Göttingen
*Joint first authors

September 21, 2018

Abstract

Word-based or 'alignment-free' sequence comparison has become an active research area in bioinformatics. While previous word-frequency approaches calculated rough measures of sequence similarity or dissimilarity, some new alignment-free methods are able to accurately estimate phylogenetic distances between genomic sequences. One of these approaches is *Filtered Spaced Word Matches*. Herein, we extend this approach to estimate evolutionary distances between complete or incomplete proteomes; our implementation of this approach is called *Prot-SpaM*. We compare the performance of *Prot-SpaM* to other alignment-free methods on simulated sequences and on various groups of eukaryotic and prokaryotic taxa. *Prot-SpaM* can be used to calculate high-quality phylogenetic trees for dozens of whole-proteome sequences in a matter of seconds or minutes and often outperforms other alignment-free approaches. The source code of our software is available through *Github*: `https://github.com/jschellh/ProtSpaM`

1 Introduction

Evolutionary relationships between species are usually inferred by comparing homologous gene or protein sequences to each other. Here, groups of orthologous sequences have to be identified first, for which then multiple alignments are to be calculated. There are generally two different strategies of resolving phylogenies based on multiple alignments. In the so-called *supermatrix* approach, multiple sequence alignments of single genes or proteins are concatenated. A phylogenetic tree is inferred from the resulting matrix, *e.g.*, using *Maximum Likelihood* [63] or *Bayesian inference* [57]. Alternatively, gene or protein trees are inferred for every single multiple sequence alignment and the resulting phylogeny is inferred using *coalescent* models [46] or supertree [4] approaches.

All these steps are time consuming, and often manual intervention is required. Therefore, *word-based* or *alignment-free* alternatives have been proposed recently, which are much faster and which require much less data preparation. Most alignment-free methods compare the *word composition* of sequences [11, 20, 31, 59, 65, 69], with some approaches also considering background word frequencies [53, 54, 60, 70], see [55] for a review of these latter approaches. More recently, the *spaced-word* composition of sequences has been used for sequence comparison [32,42,48,50]. Other alignment-free methods are based on the so-called *matching statistics*, that is they use the length of maximal common subwords [12, 68]. This has been extended to maximal common subwords with a certain number of mismatches [43, 52, 66, 67]. Alignment free approaches have been recently reviewed in detail [2,27,75].

Accurate alignment-free tools are urgently needed because of the huge volume of data generated by new sequencing techniques. Another advantage of alignment-free methods, compared to alignment-based approaches, is the fact that they can be applied to incomplete data, for example to unassembled sequencing reads or to partially sequenced genomes [18]. Note that some of the so-called 'alignment-free' approaches are based on comparing words of the input sequences to each other. So, strictly spoken, they are not 'alignment-free' since they align these words to each other. The term *alignment-free* is used nevertheless by most researchers, since these word-based approaches circumvent the need to calculate full pairwise or multiple alignments of the sequences under study.

The above mentioned approaches to alignment-free sequence comparison calculate ad-hoc measures of sequence similarity or dissimilarity. They are not based on stochastic models of molecular evolution, and they do not try

to estimate distances between sequences in a statistically rigorous way. More recently, some alignment-free approaches have been proposed that are based on explicit models of DNA evolution. These methods are able to estimate the number of substitutions per site that have happened since two nucleic-acid sequences have evolved from their last common ancestor [14,28,29,44,47,72].

A main application of alignment-free approaches is comparison of whole *genomes*. Consequently, most alignment-free methods have been designed to work on DNA sequences. If distantly related species are studied, though, phylogenetic trees are usually inferred from protein sequences rather than from DNA sequences. The reason for this is that protein sequences are more conserved than DNA sequences, as synonymous substitutions are not visible in proteins. Thus, for distal species, it may be hard to detect similarities between genes at the DNA-sequence level, while homologies may be still detectable among protein sequences. It is therefore highly desirable to have accurate alignment-free software tools that work on protein sequences, in addition to the available tools for DNA sequence comparison. Generic word-frequency methods can be applied to both DNA and protein sequences; the program *FFP*, for example, has been used to whole-proteome comparison [35]. As mentioned above, however, these methods do not estimate phylogenetic distances in a rigorous way. So far, there are no alignment-free approaches available that can accurately estimate evolutionary distances between protein sequences.

In this paper, we propose an alignment-free method which estimates the number of substitutions protein sequences since they evolved from their last common ancestor. Our approach is based on *Filtered Spaced Word Matches (FSWM)*, a concept we introduced recently for whole-genome sequence comparison [44], see [28, 72] for related approaches. We call the implementation of this new approach *Proteome-based Spaced-Word Matches (Prot-SpaM)*. The basic idea is to use gap-free pairwise alignments of fixed-length words with matching amino-acid residues at certain pre-defined positions. Such *spaced-word matches* can be rapidly identified and, after discarding random background matches, the remaining 'homologous' spaced-word matches can be used to estimate the phylogenetic distance between two taxa. To our knowledge, this is the first approach that accurately estimates evolutionary distances between protein sequences without the need to calculate full sequence alignments.

To evaluate our approach, we used simulated protein sequences and real-world whole proteomes. Test runs on the simulated sequences show that our distance estimates are very close to the true distances, for distance values of up to around 2.0 substitutions per sequence position. On the

real-world sequences, we evaluated our approach indirectly, by phylogenetic analysis, as is common practice in the field. We used *Prot-SpaM* to estimate pairwise distances for various sets of taxa, and we applied the *Neighbor-Joining* algorithm [58] to calculate phylogenetic trees from the resulting distance matrices. These trees were finally evaluated by comparing them to reference trees that were determined by standard methods and can be considered to be reliable. We show that the trees obtained with our approach are often of high quality, and they are generally more similar to the respective reference trees than trees generated with other alignment-free approaches.

2 Method

We consider sequences over an alphabet $\mathcal{A}$. In this paper, $\mathcal{A}$ consists of 20 characters representing the 20 different amino acids. *Prot-SpaM* is based on so-called *spaced-word matches* between sequences. For a *wildcard character* '$*$' with $* \notin \mathcal{A}$ and a binary pattern P of length ℓ – *i.e.* for a length-ℓ word P over $\{0, 1\}$ –, a *spaced-word* with respect to P is a length-ℓ word W over the alphabet $\mathcal{A} \cup \{*\}$ such that $W(i) = *$ if and only if $P(i) = 0$. An index $i \in \{1, \ldots, \ell\}$ is called a *match position* of P or W, respectively, if $P(i) = 1$, and a *don't care position* otherwise. The number of *match* positions in a pattern or spaced-word is called its *weight* w. We say that a spaced word W with respect to P occurs in a sequence S at some position i if one has $W(k) = S(i + k - 1)$ for all $k \in \{1, \ldots, \ell\}$ with $P(k) = 1$ – *i.e.* for all *match positions* of P.

Moreover, we say that there is a *spaced-word match* w.r.t. P between two sequences S_1 and S_2 at (i_1, i_2) if the same spaced word w.r.t P occurs at position i_1 in S_1 and at position i_2 at S_2. In other words, there is a spaced-word match between S_1 and S_2 at (i_1, i_2), if and only if one has $S_1(i_1 + k - 1) = S_2(i_2 + k - 1)$ for all match positions k of P. Below is an example for a spaced-word match between two sequences S_1 and S_2 at $(2, 3)$ with respect to the pattern $P = 1100101$; the spaced word $TN**D*P$ occurs at position 2 in S_1 and at position 3 in S_2:

S_1 :		T	T	N	Q	I	D	L	P	P	C	Y	N
S_2 :	A	C	T	N	L	I	D	I	P	Q	N		
P :			1	1	0	0	1	0	1				

Similar to our original *FSWM* approach, we estimate distances between protein sequences based on selected spaced-word matches between them,

with respect to one or several pre-defined patterns. Distance values are obtained by comparing the amino-acid residues that are aligned to each other at the *don't-care* positions of the selected spaced-word matches. This is similar to estimating distances in standard alignment-based approaches – the only difference to those standard approaches is that we are using *don't-care positions* of spaced-word matches instead of full sequence alignments.

To estimate distances in this way, one has to make sure that only those spaced-word matches are selected that represent *homologies*, *i.e.* that the involved spaced-word occurrences go back to the same origin in the last common ancestor of the two proteins that are compared. To distinguish such 'homologous' spaced-word matches from random background matches, we calculate a *score* for each spaced-word match using the BLOSUM62 substitution matrix [30]. Similar to the previous version of our program for nucleic-acid sequences, we define the *score* of a spaced-word match as the sum of substitution scores of the aligned amino acids at the *don't-care* positions. Based on this score, our algorithm decides if a spaced-word match is homologous or not: if its score is below a certain threshold T, then a spaced-word match is considered a random match and is not further considered. As default we use a threshold value of $T = 0$. To see that this threshold accurately separates homologous from background spaced-word-matches, one can plot the *number* of spaced-word matches with a score s against s, see Figure 1; we call such a plot a *Spaced-word-Match histogram* or *spamogram*, for short. In these plots, two peaks are typically visible, a peak on the right-hand side representing *homologous* spaced-word matches and a peak on the left-hand side representing *background* matches. By default, we are using patterns with a weight of $w = 6$ and with 40 *don't-care* positions, *i.e.* with a length of $\ell = 46$.

Moreover, we use a one-to-one mapping of spaced-word occurrences. Note that, if sequences S_1 and S_2 are compared and a spaced word W occurs n time in S_1 and n' times in S_2, than this gives rise to a total of $n \times n'$ spaced-word matches. Taking all these spaced-word matches into account for phylogeny reconstruction, would over-emphasize repeated regions where the same spaced words occur multiple times. Instead of using all possible spaced-word matches, we therefore use a one-to-one mapping of spaced-word occurrences in the compared sequences. That is, we ensure, that each spaced word occurrence is involved in most one of the selected spaced-word matches. Formally, if there are two spaced word matches, at (i_1, i_2) and at (j_1, j_2), respectively, then we can include both of them simultaneously in our list of selected spaced-word matches, only if $i_1 \neq j_1$ and $i_2 \neq j_2$ hold. To achieve this, we use the same *greedy* algorithm that we described in our

previous paper [44]: for a given spaced word W, we calculate the scores of all spaced-word matches involving W. We then select them one-by-one in descending order of their scores – always ensuring that each *occurrence* of W is used in at most one of the selected spaced-word matches.

Finally, in order to estimate pairwise distances between two input sequences, we consider the pairs of amino acids aligned to each other at the don't-care positions of the selected spaced-word matches. Here, we are using the *Kimura* model [38] that approximates the *PAM* distance [13] between sequences based on the number of mismatches per position. We are using these two different models since the *Kimura* model is commonly used to infer distances from the number of mismatches per position in alignments. The *BLOSUM* matrices, on the other hand, are standard in homology searching. Generally, our procedure to filter out background spaced-word matches is rather robust since the homologous and background regions in our *spamograms* can be easily distinguished as can be seen, for example, in Figure 1. So the choice of the substitution matrix to distinguish homologous from background spaced-word matches does not affect the results of our approach too much.

The accuracy and statistical stability of the described approach depends on the number of selected spaced-word matches: the more matches we obtain, the more accurate and stable the results of our method will be. To increase the number of spaced-word matches, the default version of our program uses *multiple* patterns, instead of one single pattern P. More precisely, we are using a set $\mathcal{P} = \{P_1, \ldots, P_m\}$ of m binary patterns, such that all patterns in $\mathcal{P}$ have the same length ℓ and the same weight w, but have their *match* and *don't-care* positions arranged differently; we then use spaced-word matches with respect to *all* patterns $P_i \in \mathcal{P}$. By default, our program uses sets of $m = 5$ patterns. To find suitable patterns sets, we integrated the tool *rasbhari* [25] into our implementation. *rasbhari* uses a *hill climbing* algorithm to optimize pattern sets according to a user-defined criterion. In our program, we use *rasbhari* to minimize the *overlap complexity* [33] of pattern sets. Note that *rasbhari* uses a probabilistic algorithm. It is therefore possible that different program runs of *rasbhari* return different pattern sets, even if the same parameter values are used. Consequently, different runs of *Prot-SpaM* on the same sequences and with the same parameter setting can produce slightly different distance estimates.

3 Results

To assess the quality of our new approach and to compare it to other alignment-free methods, we used artificially generated as well as real-world protein sequences. For the test runs we used the default parameters of our program, namely 6 match positions and 40 don't care positions – *i.e.* a total pattern length of 46 –, a threshold of $T = 0$ to discard background spaced-word matches, and sets of $m = 5$ patterns. We compared our program to four other alignment-free methods that can be run on protein sequences, namely *ACS* [68], *FFP* [35, 59], *kmacs* [43] and *CVTree* [53]. Here, we used version 3.19 of *FFP*, the other programs that we evaluated did not have version numbers at the time of writing. Since the original implementation of *ACS* is not publicly available, we used our own implementation of this approach by running *kmacs* with $k = 0$. The competing tools, too, were used with their default parameters. In addition to evaluating these tools on protein sequences, we ran *Filtered Spaced Word Matches* on the complete *genome* sequences of the same taxa. All test runs were done on a 10 x *Intel(R) Xeon(R)* CPU E7-4850 with 2.00*GHz* with 4 cores each summing up to 40 cores and 1000*GB RAM*.

3.1 Distance Estimation on Simulated Sequences

To evaluate the distances estimated by our program, we simulated sequences with the tool *pyvolve* [61]. *Pyvolve* simulates sequences along an evolutionary tree using continuous-time Markov models. It can use various substitution models such as *JTT* [34] and other models. Since there are no reliable stochastic models for insertions and deletions in protein sequences, the program produces indel-free sequences. We simulated pairs of sequences of length 100,000 with distances between 0 and 2 substitutions per position, in steps of 0.05, using the *JTT* model. To evaluate the estimated distance values, we generated 1,000 sequence pairs for each distance value and plotted the average of the estimated distances against the real *Kimura* distance of the respective sequence pairs, calculated with the program *protdist* from the *phylip* package [19]. To study the robustness of the estimated distances, we added error bars representing standard deviations to the plot. In addition to running *Prot-SpaM* with default parameters – *i.e.* with sets $\mathcal{P}$ of $m = 5$ patterns –, we did a second series of test runs with $m = 1$, *i.e.* with single patterns. Figure 2 shows the results of these test runs.

3.2 Phylogenetic tree reconstruction

Next, we applied the above alignment-free methods to calculate phylogenetic trees from real-world protein sequences. For four different groups of species, we downloaded all available protein sequences from *GenBank* [1]; within each group, we calculated all pairwise distances between the species. We used the distance matrices obtained in this way as input for *Neighbor-Joining* [58] and compared the resulting trees to reference trees which we assume to reflect the respective correct phylogeny for each group. The *Robinson-Foulds (RF)* distances [56] between the reconstructed trees and the respective reference trees are shown in Table 2.

As mentioned above, *Prot-SpaM* uses a probabilistic algorithm to generate pattern sets, so the results of different program runs on a sequence set can slightly differ, even if the same parameter values are used. We therefore performed 100 program runs on each data set, and Table 2 reports the *average RF* distances for these 100 program runs. An exception was the large prokaryotic data set where we only performed one single program run. Since *absolute RF* distances are not easy to interpret, Table 2 also reports the *relative RF* distances, which are obtained from the *absolute RF* distances by dividing by the maximum possible *RF* distance for a given data set. The maximal possible *RF* distances for a set of n taxa is $2 \cdot n - 6$ [9]. Program run times for the different approaches are shown in Table 3. Trees were visualized with *iTOL* [45]. *Neighbor-Joining* trees and *Robinson-Foulds* distances were calculated with the *phylip* package [19].

E. coli / Shigella

Our first data set consists of 29 strains of *Escherichia coli* and *Shigella*. For each strain, we were able to download about 4,000-5,000 protein sequences; the total size of this data set is around 41 *MB*. Figure 5 shows the reference tree that we used and the tree obtained with the algorithm described in this paper. The reference tree was published by Zhou *et al.* [74] and is based on a multiple sequence alignment of 2,034 core genes and a *Maximum Likelihood* method. As can be seen in Table 2, our approach produced a tree with a topology almost identical to the reference tree. All of the 100 program runs that we performed with *Prot-SpaM* produced the same tree topology; the *RF* distance between these trees and the reference tree was 4. The other protein-based alignment-free methods led to phylogenies with *RF* distances to the reference tree between 24 and 42, while the genome-based tree obtained with *FSWM* had a *RF* distance of 6 to the reference tree.

These trees are shown in the supplementary material.

Wolbachia

As a second test case for benchmarking, we analysed the phylogeny of *Wolbachia* strains, a group of Alphaproteobacteria which are intracellular endosymbionts of arthropods and nematodes [71]. Within *Wolbachia*, 16 distinct genetic lineages (supergroups) are currently distinguished (named by capital letters A-F and H-Q), which may differ in host specificity and type of symbiosis [24]. We re-analyzed a phylogenomic data set by [22], thereby focusing on relationships of strains within supergroups (*Wolbachia I*). A tree generated with *Prot-SpaM* from this data set is shown Figure 4.

For a second *Wolbachia* benchmarking data set, we analysed relationships between supergroups based on available (draft) genomes, see below (*Wolbachia II*). For within supergroup relationships (*Wolbachia I*), a program run of *Prot-SpaM* on the whole proteome recovered a tree which is largely congruent in topology and branch lengths in comparison to a phylogenomic supermatrix analysis of 252 single-copy orthologs which excluded genes which showed signs of recombination. A comparison based on *RF* distances showed that our new method out-competes other available alignment-free programs (Table 2). Interestingly, when only analysing the 252 ortholog data set of [22] instead of whole proteomes, *RF* distances become bigger, and other alignment-free method perform better (Table 2).

Analysing relationships between supergroups has been historically regarded as difficult phylogenetic problem [5, 23]. Analysing all annotated proteins from available genomes with *Prot-SpaM* supported the monophyly of all supergroups. Moreover, this analysis found the same *Wolbachia* strains basally branching as recent analyses suggested. Surprisingly, the phylogenomic supermatrix analysis analysis of 252 single-copy orthologs which excluded genes which showed signs of recombination of this data set recovered a topology which differs to previous study in not supporting the sister group relationship of supergroups A and B. In contrast, as found in previous analyses, the sister group relationship of supergroups A and B is supported by the *Prot-SpaM* analysis. The *Prot-SpaM* analysis also recovered some relationships between supergroups which differ from the topologies of our phylogenomic analysis or expectations from a recently published phylogenomic study [7]. However, it is known that supergroups differ in their base (and amino acid) composition, and it is currently unknown how this may impact alignment free methods. More sophisticated evolutionary models could alleviate these differences in future studies. Nevertheless, in this test

case *Prot-SpaM* also outperforms other alignment free methods when comparing the resulting phylogenetic tree with a phylogenomic analyses based on a concatenated supermatrix (Table 2).

For the *Wolbachia II* data set, we downloaded (if available) proteomes for all available *Wolbachia* draft and fully assembled genomes (47 in total, see supplementary material for details). Proteins for *Wolbachia* strains which were lacking this information on *NCBI GenBank* were derived from translations using *GeneMark* version 2.5 [3]. We predicted groups of orthologous genes between these proteomes using *Orthofinder* version 2.1.2 [17] running under default parameters. Single copy genes present in all analysed strains (83 in total) were aligned using *MAFFT* version 7.271 with the L-INS-i algorithm [37], and tested for evidence of recombination using the pairwise homoplasy index (*PHI*) [8] with window sizes of 10, 20, 30, and 50. Recombining loci were subsequently removed from the data set and the remaining loci concatenated using *FasConCat* version 1.0 [39]. The resulting supermatrix (68 loci, 20,787 amino acid positions) was subject to partitioned *Maximum Likelihood* analysis following best model and partition scheme selection in *IQ-TREE* version 1.6.2 [10, 36, 49];

For the whole-proteome sequences of the data set *Wolbachia I*, the *RF* distance to the reference tree was 6 for each of the 100 program runs. By contrast, for the 100 runs on the selected protein sequences of the same set of taxa, the average *RF* distance was 7.68, the standard deviation was 0.736. For *Wolbachia II*, the average *RF* distance was 19.62; the standard deviation was 0.89.

Large-scale microbial phylogeny reconstruction

In 2013, J. Eisen's group published a paper on the phylogeny of the microbial genomes that were available at the time [40]. As a basis of their study, they selected 24 single-copy marker genes and a non-redundant subset of taxa. To obtain such a subset, they used a greedy algorithm by M. Steel [64], making sure that marker genes from different taxa in the resulting subset had a distance to each other of at least 2 substitutions per 100 positions. This way, they obtained a non-redundant subset of 841 bacterial and archeal genomes from the more than 3,000 microbial genomes that were publicly available. Multiple sequence alignments of the marker genes were calculated with *hmmalign* [16] and were concatenated to a *supermatrix* which was used as input for the phylogeny programs *RAxML* [62] and *MrBayes* [57]. In addition, the authors used the Bayesian tree-reconciliation program *BUCKy* [41] to the same set of marker genes. The trees they obtained with these

different methods were found to be similar to trees obtained based on *16S RNA* genes.

To evaluate *Prot-SpaM*, we used the 841 microbial genomes from Lang *et al.* [40]. and downloaded all protein sequences from these taxa that were available through *GenBank*. For 28 out of the 841 taxa, we were unable to obtain protein sequences, so we obtained a slightly reduced subset of 813 taxa, compared to the taxa used by Lang *et al.* First, we applied *Prot-SpaM* to all available protein sequences from these 813 taxa. In addition, we ran *Prot-SpaM* on the protein sequences encoded by the 24 marker genes from Lang *et al.* and, finally, we applied our previous approach *Filtered Spaced Word Matches* [44] to the 841 genome sequences. The trees that we obtained with our different alignment-free approaches are shown in Figure 6, together with the *Maximum Likelihood* tree from [40] which we considered as a reliable reference. Clades from this reference tree are color-coded in Figure 6. As can be see from the color coding, the tree obtained with *Prot-SpaM* from the available protein sequences contains essentially the same clades as the reference tree. There are some differences within the clades, though, that should be further investigated (J. Eisen, personal communication). The *RF* distance between the tree obtained with *Prot-SpaM* and the reference tree was 1,020.

Plants

Next, we used a set of plant taxa that has been previously studied by Hatje and Kollmar [26] and that we had already used in previous studies to evaluate alignment-free approaches to genome sequence comparison [42–44]. The data set that we used in these previous papers consisted of 14 brassicales species. In *GenBank*, however, the proteomes could be downloaded only for 11 of the 14 species, so we had to limit our test runs to these 11 species. To obtain a reference tree, we used a tree that has been obtained with multiple sequence alignment and maximum likelihood as published by Hatje and Kollmar [26], Figure 3 B. From this tree, we removed the three species for which we could not obtain the proteome sequences in *GenBank*. Figure 7 shows the reference tree of the 14 original species, together with trees of the 11 species with available proteomes, calculated with the alignment-free methods that we evaluated in this paper. For the 100 program runs with *Prot-SpaM*, the average *RF* distance between the resulting trees and the reference tree from [26] was 0.82; the standard deviation was 0.9.

	# taxa	total size [MB]	Source
E. coli / Shigella	29	56.41	Zhou *et al.* [74]
Wolbachia I, 252 proteins	19	1.15	Gerth *et al.* [23]
Wolbachia I, whole proteomes	19	7.96	Gerth *et al.* [23]
Wolbachia II	47	14.78	See supplementary material
Plants	11	245.05	Hatje and Kollmar [26]
Prokaryotes	813	784.86	Lang *et al.* [40]
Metazoa	36	585.0	Borowiec *et al.* [6]

Table 1: Data sets used in this study to evaluate alignment-free methods, with number of taxa, total size and source of the reference tree.

Metazoa

Finally, we used a set of 36 proteomes from 34 *metazoan* and two choanoflagellate taxa. These taxa have been previously used by Borowiec *et al.* [6] to study the position of the Ctenophora within the phylogenetic tree of the metazoan kingdom. The same set of taxa has also been used in a study by Zhou *et al* [73] to evaluate *Maximum-Likelihood* programs for phylogeny reconstruction. As a reference tree, we used the tree published in [6]. The average *RF* distance of the *Prot-SpaM* trees to this reference tree was 27.1, with a standard deviation of 1.51.

Parameter values and number of selected spaced-word matches

Prot-SpaM has four major parameters which can be adjusted by the user: the *weight* w (=number of *match positions*) of the binary patterns and spaced words, their *length* ℓ, the number m of different binary patterns used by the program and the cut-off value T to separate homologous from background spaced-word matches. To see how these parameters influence the results of our software, and to find suitable default values, we ran *Prot-SpaM* with varying values of these four parameters. Here, we modified one parameter at a time, using the respective default values of the remaining three parameters.

	Prot-SpaM	*FSWM*	*CVTree*	*FFP*, $k = 4$	*kmacs*, $k = 10$	*ACS*
			RF distances			
E. coli / Shigella	4.00	6	24	40	42	38
Wolbachia I, 252 proteins	7.68	8	6	4	8	4
Wolbachia I, whole proteomes	6.00	6	8	16	8	12
Wolbachia II	19.62	20	44	54	26	16
Plants	0.82	0	6	8	2	6
Prokaryotes	1,020	1,348	886	1,452	880	960
Metazoa	27.1	-	40	62	30	36
			Relative *RF* distances			
E. coli / Shigella	0.08	0.12	0.46	0.77	0.81	0.73
Wolbachia I, 252 proteins	0.24	0.25	0.19	0.13	0.25	0.13
Wolbachia I, whole proteomes	0.19	0.19	0.25	0.50	0.25	0.38
Wolbachia II	0.23	0.23	0.50	0.61	0.30	0.18
Plants	0.05	0.00	0.37	0.50	0.12	0.37
Prokaryotes	0.63	0.83	0.55	0.90	0.54	0.59
Metazoa	0.41	-	0.61	0.94	0.45	0.55

Table 2: *Robinson-Foulds (RF)* distances and *relative RF* distances between trees generated with alignment-free methods and the respective reference trees for various sets of taxa, see the main text for details. Since *Prot-SpaM* uses a probabilistic algorithm, different program runs may produce slightly different results. Therefore, we performed 100 program runs on each data set and report the *average RF* distances, except for the large prokaryote data set where we did only one single program run. All programs were run on *protein* sequences or *whole proteomes*, respectively, except for *Filtered Spaced Word Matches (FSWM)*, which was run on *whole-genome sequences* of the same species (or on the gene sequences coding for the 252 selected proteins from *Wolbachia I*). We were unable to run *FSWM* on the whole genomes of the 31 *metazoan* species, since this data set was too large. Since the original implementation of *ACS* is not publicly available, we ran our own implementation, *kmacs*, with $k = 0$ instead.

	Prot-SpaM	*FSWM*	*CVTree*	*FFP*, $k = 4$	*kmacs*, $k = 10$	*ACS*
E. coli / Shigella	55	110	125	10	2,518	193
Wolbachia II	19	68	46	9	5,302	135
Wolbachia I, 252 proteins	3	5	2	1	36	3
Wolbachia I, whole proteomes	11	22	21	2	178	26
Plants	464	1,107,720	365	17	17,693	850
Prokaryotes	5,502	244,139	5,492	1,929	915,635	123,520
Metazoa	1,719	-	1,973	43	151,612	9,512

Table 3: Program run time in seconds for different alignment-free approaches on our benchmark data sets. *Prot-SpaM* and *FSWM* were run on 40 threads. The other tools do not support multi-threading, therefore they were run single threaded.

The results are summarized in Tables 4 and 5. As can be seen from these tables, there are no values for w, ℓ an T that work best for all data sets, but our default values seem to be a reasonable compromise. Using sets of $m = 5$ binary patterns does not improve the quality of the produced trees in terms of their *RF* distances to the reference trees, compared to program runs with single patterns. Table 3 shows, however, that the distance values estimated by *Prot-SpaM* become statistically more stable if multiple patterns are used.

The number of spaced-word matches in a pairwise sequence comparison depends on how similar the two sequences are to each other, see [48] for details. Consequently, the number of spaced-word matches that are selected by our program to estimate phylogenetic distances also depends on the degree of similarity between the compared sequences. We found two extreme cases with our test data, one in the *E. coli/Shigella* data set where most taxa are closely related to each other, and another one in the *Metazoan* data set that contains taxa with very large evolutionary distances. In the pairwise comparison of *E. coli O157:H7 strain EDL933* with *E. coli O157:H7 Sakai (EHEC)*, *Prot-SpaM* selected more than 6,000,000 spaced-word matches. These two proteomes have less than 1,600,000 amino acids each, so in this

case > 3.75 spaced-word matches per sequence position were selected. By contrast, less than 13.000 spaced-word matches were selected in the comparison of *Brugia malayi* and *Homo sapiens*. The latter proteome has a length of more than 75.000.000 amino acids, so here less than 0.00017 spaced-word matches per sequence position were selected.

4 Discussion

A number of so-called 'alignment-free' approaches have been proposed in recent years to rapidly calculate phylogenetic distances between genomic sequences. Earlier approaches are based on k-mer frequencies or on the length of common substrings; these approaches have been applied not only to DNA, but also to protein sequences. A draw-back of these methods is that they can only calculate rough measures of sequence similarity or dissimilarity, they do not estimate phylogenetic distances in a rigorous way. More recently, word-based methods have been developed that can accurately estimate phylogenetic distances between genomic sequences based on stochastic models of DNA evolution. One of these approaches is *Filtered-Spaced Word Matches (FSWM)*.

In this study, we introduced *Prot-SpaM*, a new implementation of *FSWM* to compare complete or incomplete *proteome sequences* to each other. To our knowledge, *Prot-SpaM* is the first tool that can accurately estimate phylogenetic distances between protein sequences without the need to calculate full sequence alignments. Our benchmark results show that distance estimates obtained with our approach are accurate for a large range of phylogenetic distances. Distances calculated with *CVTree, ACS, FFP* and *kmacs*, by comparison, are monotonously increasing with the number of substitutions between the compared sequences. The obtained distance values are far from proportional to the real distances, though, and they flatten out somewhere between 0.5 and 1.5 substitutions per position, see Figure 2. By contrast, *Prot-SpaM* estimates distances with high accuracy for up to around 2.0 substitutions per position. For higher distance values, the calculated distances become less stable, as can be seen from the error bars in Figure 2. Moreover, for large distances, our program tends to slightly overestimate distances.

In our program evaluation, *Prot-SpaM* produced high-quality trees and was superior to other alignment-free methods for the *E.coli/Shigella* and the plant data sets, as shown in Table 2. On the *Wolbachia* data sets, it still performed reasonably well and was again superior to competing approaches on the whole-proteome sequences, but it was outperformed by word-frequency

			E. coli/Shigella					
Weight w		**6**	8	10				
Runtime [s]		**55.4**	47.4	46.9				
RF distance		**4**	6.02	6.76				
Length ℓ	36	**46**	56	66				
Runtime [s]	47.5	**55.4**	60.3	66.9				
RF distance	4	**4**	4.02	4.84				
# patterns m	1	3	**5**	7				
Runtime [s]	13.5	34.4	**55.4**	75.94				
RF distance	4.12	4.02	**4**	4				
Threshold T	-50	-25	**0**	25	50	75	100	125
Runtime [s]	55.2	55.4	**55.4**	55.1	55.3	55.3	55.1	55.2
RF-Distance	11.92	12	**4**	12	12	12	12	12
			Wolbachia II					
Weight w	4	**6**	8	10				
Runtime [s]	112.4	**19.4**	18	17.8				
RF distance	20.38	**19.68**	22	22				
Length ℓ	36	**46**	56	66				
Runtime [s]	17	**19.4**	21.5	23.9				
RF distance	19.78	**19.68**	19.7	19.78				
# patterns m	1	3	**5**	7				
Runtime [s]	5.4	12.5	**19.4**	26.5				
RF distance	19.06	19.12	**19.68**	19.82				
Threshold T	-50	-25	**0**	25	50	75	100	125
Runtime [s]	19.6	19.6	**19.4**	19.4	19.4	19.4	19.4	19.4
RF-Distance	22.18	18	**19.68**	19.86	20.16	20.7	21.04	22

Table 4: Program runtime and *Robinson-Foulds* distances to reference trees for different parameter values with *Prot-SpaM* for the *E. coli/Shigella* and *Wolbachia* proteomes. We ran our program with different values for the *weight* w and length ℓ of the spaced-words, for different numbers of patterns and for different values of the threshold T. Here, we modified the value of one of these parameters at a time and used the default values for the remaining three parameters. Default values of the modified parameters and the resulting runtimes and *RF* distances are shown in bold font. Since *Prot-SpaM* uses a probabilistic algorithm to generate pattern sets, we performed 100 program runs for each set of parameters; the table reports the average *RF* distances of these 100 runs.

Plants

Weight w	4	**6**	8	10				
Runtime [s]	57,578	**464**	320	325				
RF distance	2	**0**	4	6				
Length ℓ	36	**46**	56	66				
Runtime [s]	383	**464**	441	494				
RF distance	2	**0**	0	0				
# patterns m	1	3	**5**	7				
Runtime [s]	91	255	**464**	572				
RF distance	0	0	**0**	2				
Threshold T	-50	-25	**0**	25	50	75	100	125
Runtime [s]	383	402	**464**	409	391	459	439	430
RF-Distance	2	2	**0**	0	0	0	2	4

Metazoa

Weight w		**6**	8	10				
Runtime [s]		**1719**	1584	1518				
RF distance		**30**	26	30				
Length ℓ	36	**46**	56	66				
Runtime [s]	1351	**1719**	1584	2089				
RF distance	26	**30**	24	26				
# patterns	1	3	**5**	7				
Runtime [s]	427	890	**1719**	2078				
RF distance	24	28	**30**	26				
Threshold T	-50	-25	**0**	25	50	75	100	125
Runtime [s]	2539	2337	**1719**	2269	2150	1906	1783	1797
RF-Distance	30	24	**30**	26	28	28	30	34

Table 5: Program runtime and *Robinson-Foulds* distances to reference trees for different parameter values with *Prot-SpaM* for the *plant* and *metazoan* proteomes; parameter values as in Table 4. Because of the size of these data sets, we performed only one program run per parameter set.

methods on the 252 selected orthologous proteins. A possible explanation of this result is discussed below. On the large prokaryote data set and for the metazoan set, by contrast, none of the compared programs could reproduce the reference trees that we used in our evaluation. These are difficult data sets since they span very large evolutionary distances; also it should be mentioned that there are no absolutely reliable reference trees available for these data sets. For the metazoan data set, for example, the positions of the ctenophores is still a matter of debate [15, 21, 51]. On the metazoans, *Prot-SpaM* performed better than other alignment-free approaches, while on the large prokaryote data set, *CVTree, ACS* and *kmacs* were superior.

An interesting result is the performance of *Prot-SpaM*, compared to our previous approach *FSWM* that takes genomic sequences as input. For most groups of taxa in our study, the results of *Prot-SpaM* and *FSWM* were of similar quality, in the sense that the *RF* distances to the reference trees were comparable for both approaches. However, for the set of 813 prokaryote taxa, our new spaced-words approach performed better on whole-proteomes than our previous approach on whole genomes, as is shown in Figure 6 and Table 2. This discrepancy is most likely due to the large phylogenetic distances in this data set; for such distantly related sequences, homologies are generally better detectable at the protein level than at the DNA level.

'Alignment-free' methods to phylogeny reconstruction can be directly applied to whole-genome or whole-proteome sequences, without the need to select orthologous genes or proteins in a first step. This is generally seen as an advantage over more traditional, alignment-based approaches, since the task of finding orthologs is time-consuming and often involves manual intervention. On our data set *Wolbachia I*, we actually obtained better *RF* distances with *Prot-SpaM* and *FSWM* when we applied these programs to the whole-protein or whole-genome sequences, respectively, than when we applied them to the 252 selected orthologous proteins or to the genes coding for those proteins, see Table 2. These results are in contrast to the more traditional alignment-free methods *FFP*, *CVtree* and *ACS* that are based on word-frequencies or on the length of common substrings. The latter programs performed better on the selected orthologous proteins of the *Wolbachia I* data set than on the corresponding whole-proteome sequences.

A possible explanation of this phenomenon is that *Prot-SpaM* and *FSWM* can reliably distinguish between homologous and background spaced-word matches and use only homologous matches for phylogenetic inference. With the *one-to-one* spaced-word matching, they can also reduce the number of *paralogous* spaced-word matches. Therefore, they can be applied to whole proteomes or whole genomes without being too much confused by paralogs or

by non-related parts of the sequences. Here, the benefits of using larger input sequence sets seem to outweigh the disadvantage of including possible non-related sequences, paralogs or sequences with recombinations. Previously introduced word-frequency or substring-length methods, by contrast, do not distinguish between homologous and non-homologous parts of the sequences. Therefore, these approaches tend to be confused by input sequences that contain paralogs or are only locally related to each other.

Table 3 shows that the run time of Prot-SpaM is superior to *CVtree* and *ACS* on protein sequences. By far the fastest alignment-free method on whole proteomes was *FFP*, the slowest one was *kmacs*. On the plant proteomes, *Prot-Spam* was three orders of magnitude faster than *FSWM* on the genome sequences of the same species. This is not surprising, given the fact that in eukaryotes only a small part of the genome is protein-coding sequence. The total size of the 11 plant genomes was 3.8 *GB*, compared to 245 *MB* for the corresponding proteome sequences (note that, for the genome sequences, both strands are considered and the number of background spaced-word matches scales quadratically with the sequence length). ~~the proteomes are substantially smaller than the corresponding genomes~~

Prot-SpaM has four major parameters that can be adjusted by the user: the weight w and the length ℓ of the patterns and spaced-words, respectively, the cut-off value T to distinguish homologous from random spaced-word matches and the number m of different patterns used to generate spaced-word matches. We provide default values for these parameters, but Tables 4 and 5 show that reasonable results can be obtained with a rather broad range of parameter values. These tables also show that the quality of the produced trees, as measured by the *RF* distances to the reference trees, could not be improved by using $m = 5$ patterns, compared to the single-pattern option, *i.e.* $m = 1$. The statistical stability of our distance estimates, however, is increased if multiple patterns are used; therefore, we are using $m = 5$ patterns by default. But since runtime and memory usage of our program increase with the number m of pattern, it may be advisable to use the single-pattern option if very large data sets are to be analyzed.

It should be mentioned that traditional approaches to phylogeny reconstruction that are based on multiple sequence alignment are still more accurate than alignment-free approaches that have been proposed in recent years. The main advantage of these novel approaches is their high speed, with makes it possible to apply them to the large sequence data sets that are now available; a program run of *Prot-SpaM* on whole-proteome sequences of the set *Wolbachia II* that consists of 47 taxa, took only 19 seconds. Another advantage of our approach is that it can reliably distinguish between local

homologies and random background similarities. It can, thus, be applied to complete or incomplete proteomes, and it is not necessary to select orthologous genes or proteins in a first step. Therefore, we think that *Prot-SpaM* should be a useful addition to existing approaches to phylogeny reconstruction.

Acknowledgements

We thank Jonathan Eisen for his comments on the phylogeny of the 841 microbial taxa that were studied in Lang *et al.* Five reviewers made very helpful comments on an earlier version of this manuscript. We acknowledge support by the *Open Access Publication Funds* of the *Göttingen University*.

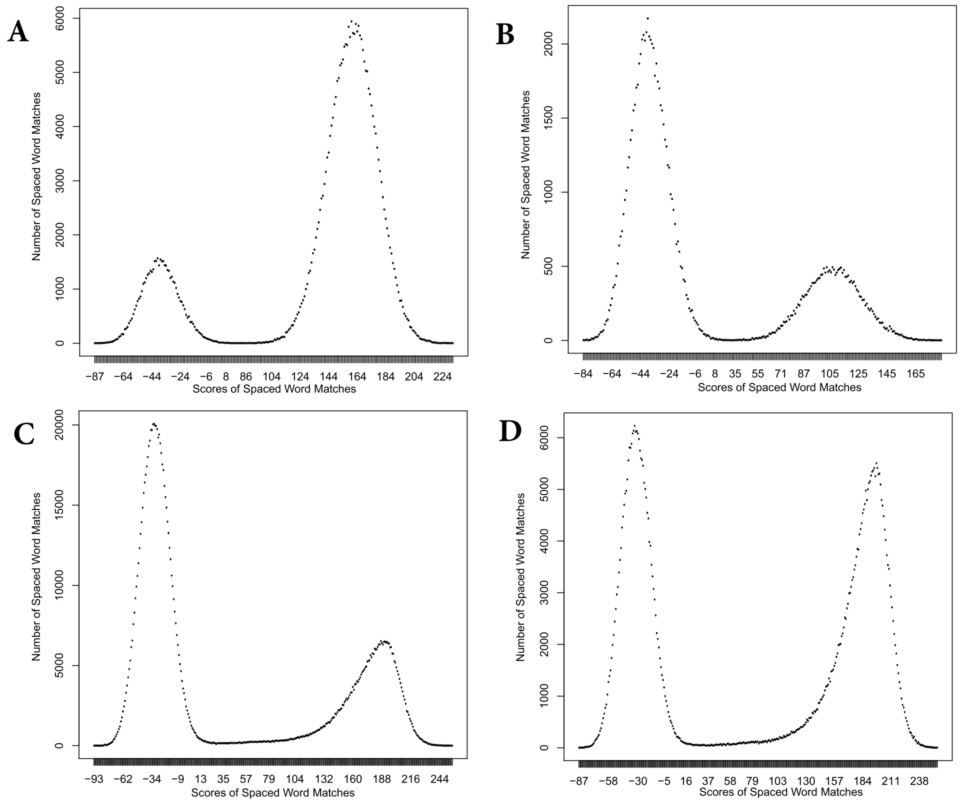

Figure 1: *Spaced-word histograms ('spamograms')* for different data sets. (A) and (B) are based on simulated indel-free protein sequences with a total length of of 1.6×10^6 amino-acid residues each, and with 0.3 (A) and 0.75 (B) substitutions per position, respectively. (C) and (D) are from a whole-proteome comparisons of plants, (C) comparing *Eucalyptus grandis* with *Capsella rubella* and (D) comparing *Gossypium raimondii* with *Carica papaya*.

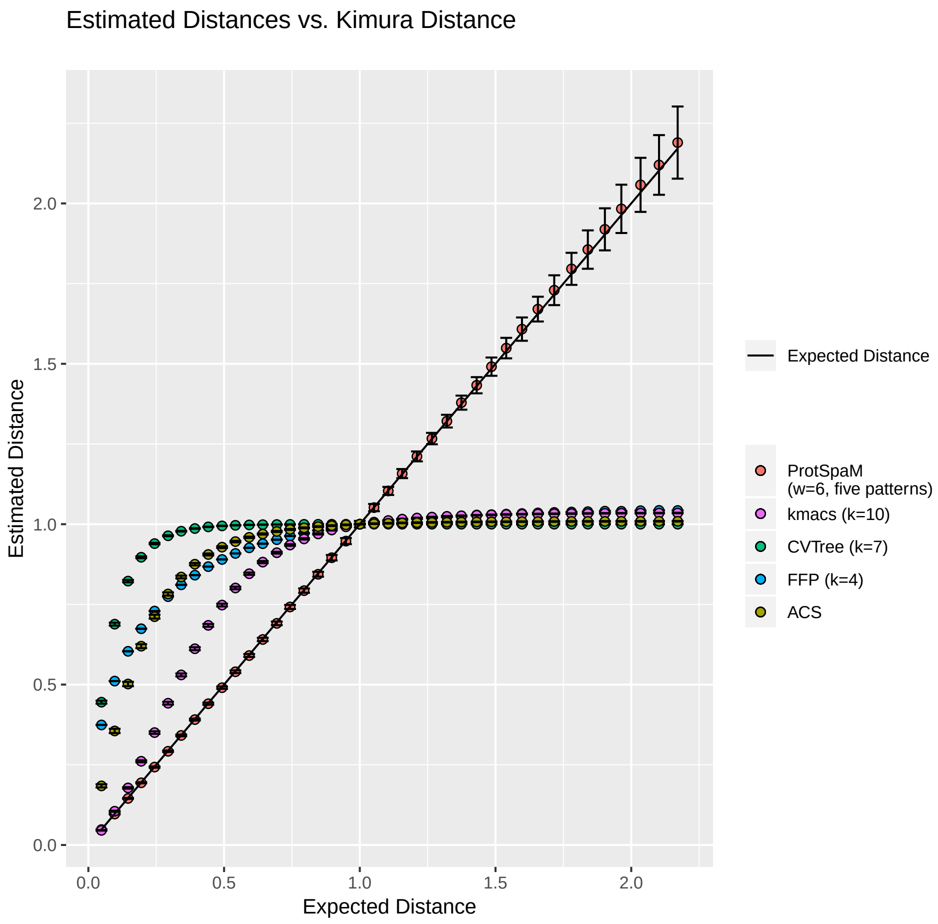

Figure 2: Distances calculated by *Prot-SpaM* and four other alignment-free methods calculated for pairs of simulated protein sequences, plotted against their distances calculated with the *Kimura* model. Error bars denote standard deviations. Note that *Prot-SpaM* estimates phylogenetic distances in terms of substitutions that have happened since two sequences evolved from their lasts common ancestor. The programs *kmacs, CVTree, FFP* and *ACS*, by contrast, do not estimate distances in a rigorous way, but rather use ad-hoc measures of sequence dissimilarity that are not linear functions of the real distances. Also, the absolute values of these distance measures are rather arbitrary for these four other programs. We therefore normalized the distances calculated by *kmacs, CVTree, FFP* and *ACS* such that they have a value of one for sequence pairs with a *Kimura* distance of one.

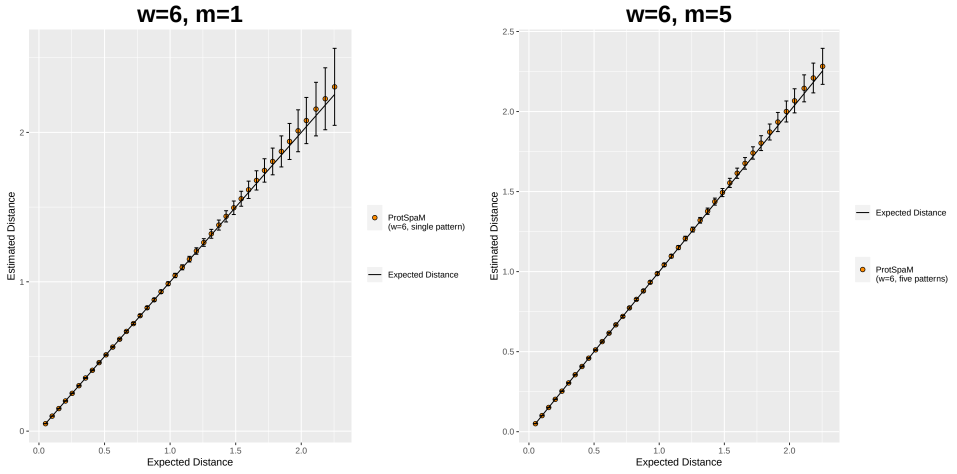

Figure 3: Distances calculated by *Prot-SpaM* for pairs of simulated protein sequences with a single binary pattern ($m = 1$, left) and with the default multiple-pattern option ($m = 5$, right). We performed 1000 program runs for each value of m. The plot shows the *average* of the calculated distances; *standard deviations* are shown as error bars.

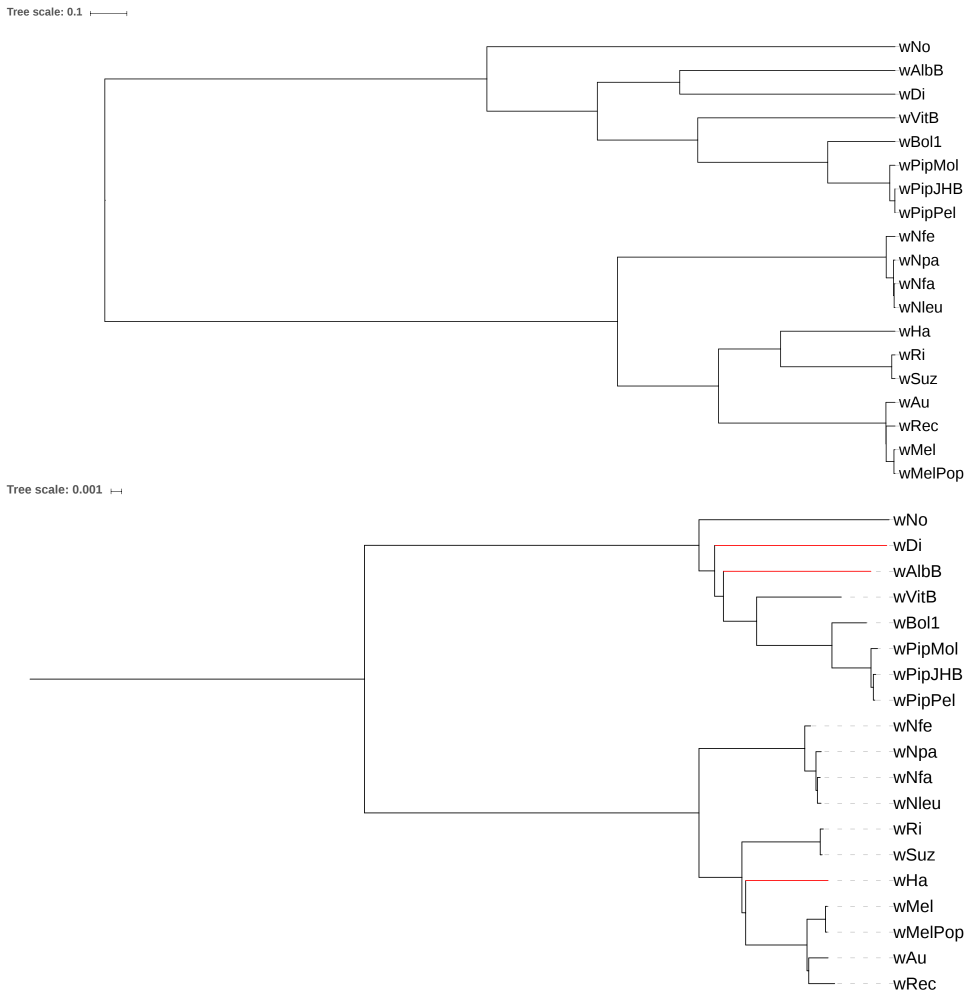

Figure 4: Reference tree for our data set *Wolbachia I* (above) and tree calculated with *Prot-SpaM* using whole-proteome sequences of the same taxa (below), see main text for details. Topological differences between the two trees are shown in red in the *Prot-SpaM* tree.

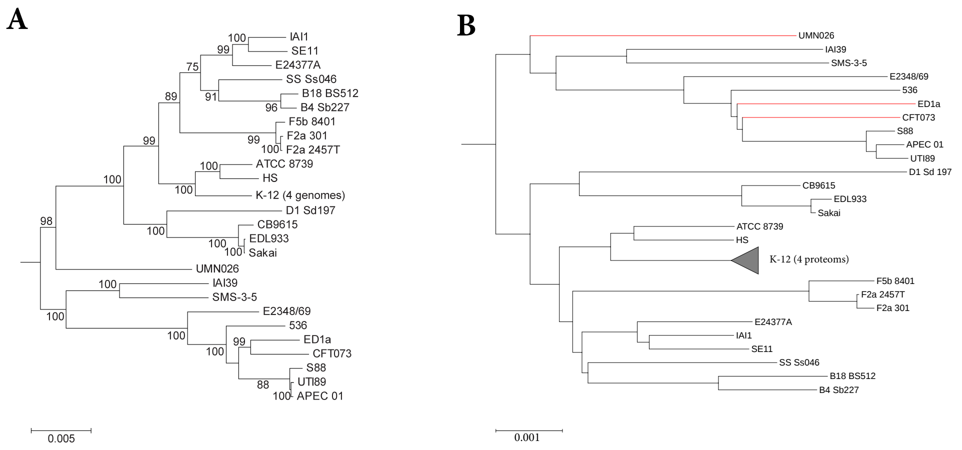

Figure 5: Reference tree (A) from [74] and tree calculated with *Prot-SpaM* with default parameters (B) for a set of 29 *Escherichia coli* and *Shigella* strains. Differences in the topologies between the two trees are marked in red.

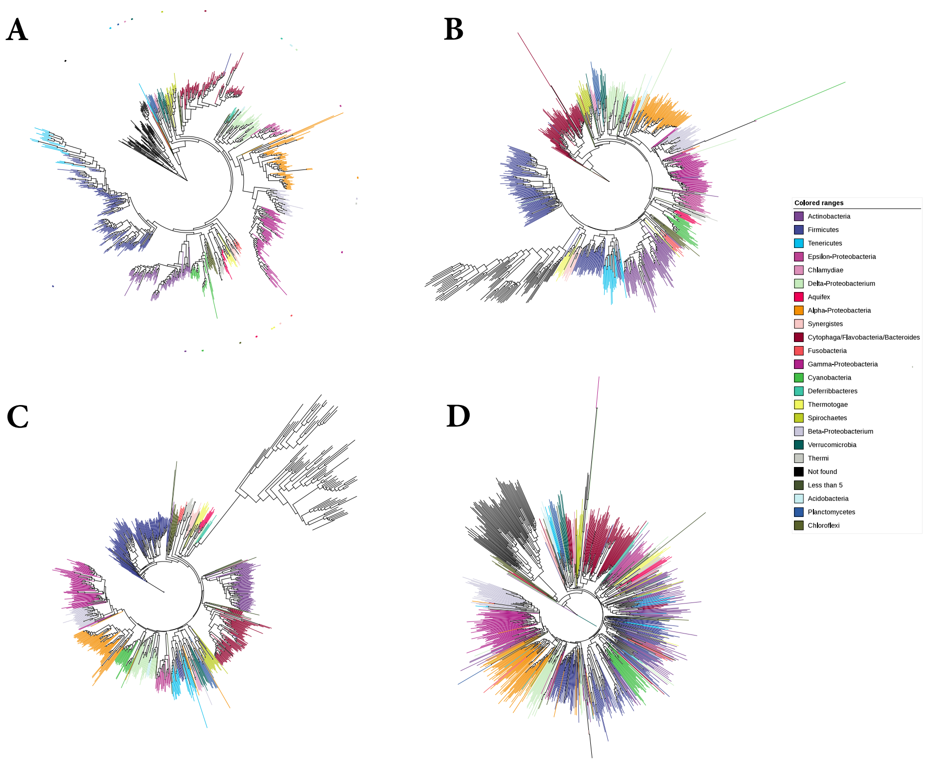

Figure 6: Phylogenetic trees for a large set of microbial taxa studied by Lang *et al.* [40]. (A) Maximum-Likelihood tree constructed by Lang *et al.* based on a super alignment of 24 selected genes, (B) tree constructed with our approach, as described in this paper, for 813 taxa for which the proteomes are available in *GenBank*, (C) tree constructed with our approach based on the proteins corresponding to the 24 genes selected by Lang *et al.* and (D) tree reconstructed using our program *FSWM* [44] on the 841 whole-genome sequences.

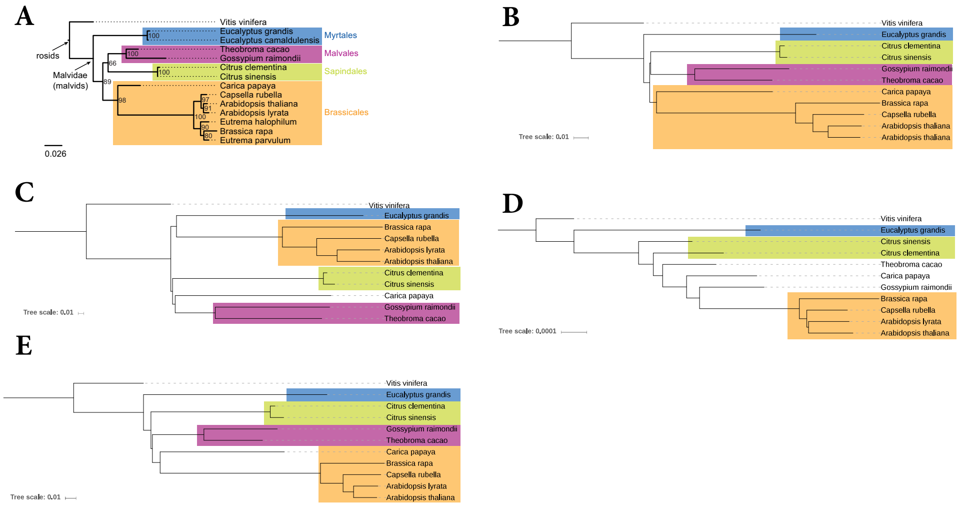

Figure 7: Phylogenetic trees of plant taxa. (A) reference tree from [26], and trees constructed with (B) the approach described in this paper, (C) *ACS* [68], (D) *FFP* [59], and (E) *kmacs* [43]. The original data set contained 14 taxa, but only for 11 taxa, the proteomes could be downloaded through *GenBank*. For completeness, we show the reference for all 14 taxa.

References

[1] Dennis A Benson, Mark Cavanaugh, Karen Clark, Ilene Karsch-Mizrachi, James Ostell, Kim D Pruitt, and Eric W Sayers. Genbank. *Nucleic Acids Research*, 46(D1):D41–D47, 2018.

[2] Guillaume Bernard, Cheong Xin Chan, Yao-ban Chan, Xin-Yi Chua, Yingnan Cong, James M. Hogan, Stefan R. Maetschke, and Mark A. Ragan. Alignment-free inference of hierarchical and reticulate phylogenomic relationships. *Briefings in Bioinformatics*, in press:bbx067, 2017.

[3] John Besemer and Mark Borodovsky. GeneMark: web software for gene finding in prokaryotes, eukaryotes and viruses. *Nucleic Acids Research*, 33:W451–W454, 2005.

[4] Olaf R.P. Bininda-Emonds. The evolution of supertrees. *Trends in Ecology and Evolution*, 19:315 – 322, 2004.

[5] Seth R. Bordenstein, Charalampos Paraskevopoulos, Julie C. Dunning Hotopp, Panagiotis Sapountzis, Nathan Lo, Claudio Bandi, Herv Tettelin, John H. Werren, and Kostas Bourtzis. Parasitism and mutualism in Wolbachia: What the phylogenomic trees can and cannot say. *Molecular Biology and Evolution*, 26:231–241, 2009.

[6] Marek L. Borowiec, Ernest K. Lee, Joanna C. Chiu, and David C. Plachetzki. Extracting phylogenetic signal and accounting for bias in whole-genome data sets supports the Ctenophora as sister to remaining Metazoa. *BMC Genomics*, 16:987, 2015.

[7] Amanda M. V. Brown, Sulochana K. Wasala, Dana K. Howe, Amy B. Peetz, Inga A. Zasada, and Dee R. Denver. Genomic evidence for plant-parasitic nematodes as the earliest Wolbachia hosts. *Scientific Reports*, 6:34955, 2016.

[8] Trevor C. Bruen, Hervé Philippe, and David Bryant. A simple and robust statistical test for detecting the presence of recombination. *Genetics*, 172:2665–2681, 2006.

[9] David Bryant and Mike Steel. Computing the distribution of a tree metric. *IEEE/ACM Trans. Comput. Biol. Bioinformatics*, 6:420–426, 2009.

[10] Olga Chernomor, Arndt von Haeseler, and Bui Quang Minh. Terrace aware data structure for phylogenomic inference from supermatrices. *Systematic Biology*, 65:997–1008, 2016.

[11] Benny Chor, David Horn, Yaron Levy, Nick Goldman, and Tim Massingham. Genomic DNA k-mer spectra: models and modalities. *Genome Biology*, 10:R108, 2009.

[12] Matteo Comin and Davide Verzotto. Alignment-free phylogeny of whole genomes using underlying subwords. *Algorithms for Molecular Biology*, 7:34, 2012.

[13] Margaret O. Dayhoff, Robert M. Schwartz, and Bruce C. Orcutt. A model of evolutionary change in proteins. *Atlas of Protein Sequence and Structure*, 6:345–362, 1978.

[14] Thomas Dencker, Chris-André Leimeister, and Burkhard Morgenstern. *Multi-SpaM*: a maximum-likelihood approach to phylogeny reconstruction based on multiple spaced-word matches. *arXiv*, 1803.09222[q-bio.PE], 2018.

[15] Casey W. Dunn, Gonzalo Giribet, Gregory D. Edgecombe, and Andreas Hejnol. Animal phylogeny and its evolutionary implications. *Annual Review of Ecology, Evolution, and Systematics*, 45:371–395, 2014.

[16] Sean R. Eddy. A new generation of homology search tools based on probabilistic inference. In *Genome Informatics 2009 - Proceedings of the 20th International Conference*, pages 205–211. Imperial College Press, 2009.

[17] David M. Emms and Steven Kelly. OrthoFinder: solving fundamental biases in whole genome comparisons dramatically improves orthogroup inference accuracy. *Genome Biology*, 16:157, 2015.

[18] Huan Fan, Anthony R. Ives, Yann Surget-Groba, and Charles H. Cannon. An assembly and alignment-free method of phylogeny reconstruction from next-generation sequencing data. *BMC Genomics*, 16:522, 2015.

[19] Joseph Felsenstein. PHYLIP - Phylogeny Inference Package (Version 3.2). *Cladistics*, 5:164–166, 1989.

[20] Umberto Ferraro-Petrillo, Gianluca Roscigno, Giuseppe Cattaneo, and Raffaele Giancarlo. Informational and linguistic analysis of large genomic sequence collections via efficient hadoop cluster algorithms. *Bioinformatics*, 34:18261833, 2018.

[21] Roberto Feuda, Martin Dohrmann, Walker Pett, Herv Philippe, Omar Rota-Stabelli, Nicolas Lartillot, Gert Wrheide, and Davide Pisani. Improved modeling of compositional heterogeneity supports sponges as sister to all other animals. *Current Biology*, 27:3864 – 3870.e4, 2017.

[22] Michael Gerth and Christoph Bleidorn. Comparative genomics provides a timeframe for *Wolbachia* evolution and exposes a recent biotin synthesis operon transfer. *Nature Microbiology*, 2:16241, 2016.

[23] Michael Gerth, Marie-Theres Gansauge, Anne Weigert, and Christoph Bleidorn. Phylogenomic analyses uncover origin and spread of the Wolbachia pandemic. *Nature Communications*, 5:5117, 2014.

[24] Eliza Glowska, Anna Dragun-Damian, Miroslawa Dabert, and Michael Gerth. New Wolbachia supergroups detected in quill mites (Acari: Syringophilidae). *Infection, Genetics and Evolution*, 30:140–146, 2015.

[25] Lars Hahn, Chris-André Leimeister, Rachid Ounit, Stefano Lonardi, and Burkhard Morgenstern. *rasbhari*: optimizing spaced seeds for database searching, read mapping and alignment-free sequence comparison. *PLOS Computational Biology*, 12(10):e1005107, 2016.

[26] Klas Hatje and Martin Kollmar. A phylogenetic analysis of the brassicales clade based on an alignment-free sequence comparison method. *Frontiers in Plant Science*, 3:192, 2012.

[27] Bernhard Haubold. Alignment-free phylogenetics and population genetics. *Briefings in Bioinformatics*, 15:407–418, 2014.

[28] Bernhard Haubold, Fabian Klötzl, and Peter Pfaffelhuber. andi: Fast and accurate estimation of evolutionary distances between closely related genomes. *Bioinformatics*, 31:1169–1175, 2015.

[29] Bernhard Haubold, Peter Pfaffelhuber, Mirjana Domazet-Loso, and Thomas Wiehe. Estimating mutation distances from unaligned genomes. *Journal of Computational Biology*, 16:1487–1500, 2009.

[30] Steven Henikoff and Jora G. Henikoff. Amino acid substitution matrices from protein blocks. *Proc. Natl. Acad. Sci. USA*, 89:10915–10919, 1992.

[31] Michael Höhl, Isidore Rigoutsos, and Mark A. Ragan. Pattern-based phylogenetic distance estimation and tree reconstruction. *Evolutionary Bioinformatics Online*, 2:359–375, 2006.

[32] Sebastian Horwege, Sebastian Lindner, Marcus Boden, Klaus Hatje, Martin Kollmar, Chris-André Leimeister, and Burkhard Morgenstern. *Spaced words* and *kmacs*: fast alignment-free sequence comparison based on inexact word matches. *Nucleic Acids Research*, 42:W7–W11, 2014.

[33] Lucian Ilie, Silvana Ilie, and Anahita M. Bigvand. SpEED: fast computation of sensitive spaced seeds. *Bioinformatics*, 27:2433–2434, 2011.

[34] David T. Jones, William R. Taylor, and Janet M. Thornton. The rapid generation of mutation data matrices from protein sequences. *Bioinformatics*, 8(3):275–282, 1992.

[35] Se-Ran Jun, Gregory E. Sims, Guohong A. Wu, and Sung-Hou Kim. Whole-proteome phylogeny of prokaryotes by feature frequency profiles: An alignment-free method with optimal feature resolution. *Proceedings of the National Academy of Sciences*, 107:133–138, 2010.

[36] Subha Kalyaanamoorthy, Minh Q. Bui, Thomas K F Wong, Arndt von Haeseler, and Lars S. Jermiin. ModelFinder: fast model selection for accurate phylogenetic estimates. *Nature Methods*, 14:587–589, 2017.

[37] Kazutaka Katoh and Daron M. Standley. MAFFT multiple sequence alignment software version 7: Improvements in performance and usability. *Molecular Biology and Evolution*, 30:772–780, 2013.

[38] Motoo Kimura. *The Neutral Theory of Molecular Evolution.* Cambridge University Press, 1983.

[39] Patrick Kück and Gary C. Longo. FASconCAT-G: extensive functions for multiple sequence alignment preparations concerning phylogenetic studies. *Frontiers in Zoology*, 11:81, 2014.

[40] Jenna Morgan Lang, Aaron E. Darling, and Jonathan A. Eisen. Phylogeny of bacterial and archaeal genomes using conserved genes: Supertrees and supermatrices. *PLOS ONE*, 8:e62510, 2013.

[41] Bret R. Larget, Satish K. Kotha, Colin N. Dewey, and Cécile Ané. BUCKy: Gene tree/species tree reconciliation with Bayesian concordance analysis. *Bioinformatics*, 26:2910–2911, 2010.

[42] Chris-André Leimeister, Marcus Boden, Sebastian Horwege, Sebastian Lindner, and Burkhard Morgenstern. Fast alignment-free sequence comparison using spaced-word frequencies. *Bioinformatics*, 30:1991–1999, 2014.

[43] Chris-André Leimeister and Burkhard Morgenstern. *kmacs*: the k-mismatch average common substring approach to alignment-free sequence comparison. *Bioinformatics*, 30:2000–2008, 2014.

[44] Chris-André Leimeister, Salma Sohrabi-Jahromi, and Burkhard Morgenstern. Fast and accurate phylogeny reconstruction using filtered spaced-word matches. *Bioinformatics*, 33:971–979, 2017.

[45] Ivica Letunic and Peer Bork. Interactive tree of life (iTOL) v3: an online tool for the display and annotation of phylogenetic and other trees. *Nucleic Acids Research*, 44:W242–W245, 2016.

[46] Liang Liu, Zhenxiang Xi, Shaoyuan Wu, Charles C. Davis, and Scott V. Edwards. Estimating phylogenetic trees from genome-scale data. *Annals of the New York Academy of Sciences*, 1360:36–53, 2015.

[47] Burkhard Morgenstern, Svenja Schöbel, and Chris-André Leimeister. Phylogeny reconstruction based on the length distribution of k-mismatch common substrings. *Algorithms for Molecular Biology*, 12:27, 2017.

[48] Burkhard Morgenstern, Bingyao Zhu, Sebastian Horwege, and Chris-André Leimeister. Estimating evolutionary distances between genomic sequences from spaced-word matches. *Algorithms for Molecular Biology*, 10:5, 2015.

[49] Lam-Tung Nguyen, Heiko A. Schmidt, Arndt von Haeseler, and Bui Quang Minh. IQ-TREE: A fast and effective stochastic algorithm for estimating maximum-likelihood phylogenies. *Molecular Biology and Evolution*, 32:268–274, 2015.

[50] Laurent Noé. Best hits of 11110110111: model-free selection and parameter-free sensitivity calculation of spaced seeds. *Algorithms for Molecular Biology*, 12:1, 2017.

[51] Hervé Philippe, R Derelle, P Lopez, Kerstin Pick, C Borchiellini, N Boury-Esnault, J Vacelet, E Renard, E. Houliston, E. Quéinnec,

C. Da Silva, P. Wincker, H. Le Guyader, S. Leys, Daniel Jackson, Fabian Schreiber, Dirk Erpenbeck, Burkhard Morgenstern, Gert Wörheide, and M Manuel. Phylogenomics restores traditional views on deep animal relationships. *Current Biology*, 19:706–712, 2009.

[52] Cinzia Pizzi. MissMax: alignment-free sequence comparison with mismatches through filtering and heuristics. *Algorithms for Molecular Biology*, 11:6, 2016.

[53] Ji Qi, Hong Luo, and Bailin Hao. CVTree: a phylogenetic tree reconstruction tool based on whole genomes. *Nucleic Acids Research*, 32(suppl 2):W45–W47, 2004.

[54] Gesine Reinert, David Chew, Fengzhu Sun, and Michael S. Waterman. Alignment-free sequence comparison (I): Statistics and power. *Journal of Computational Biology*, 16:1615–1634, 2009.

[55] Jie Ren, Xin Bai, Yang Young Lu, Kujin Tang, Ying Wang, Gesine Reinert, and Fengzhu Sun. Alignment-free sequence analysis and applications. *Annual Review of Biomedical Data Science*, 1:93–114, 2018.

[56] David F Robinson and Les Foulds. Comparison of phylogenetic trees. *Mathematical Biosciences*, 53:131–147, 1981.

[57] Fredrik Ronquist and John P. Huelsenbeck. MrBayes 3: Bayesian phylogenetic inference under mixed models. *Bioinformatics*, 19:1572–1574, 2003.

[58] Naruya Saitou and Masatoshi Nei. The neighbor-joining method: a new method for reconstructing phylogenetic trees. *Molecular Biology and Evolution*, 4:406–425, 1987.

[59] Gregory E. Sims, Se-Ran Jun, Guohong A. Wu, and Sung-Hou Kim. Alignment-free genome comparison with feature frequency profiles (FFP) and optimal resolutions. *Proceedings of the National Academy of Sciences*, 106:2677–2682, 2009.

[60] Kai Song, Jie Ren, Zhiyuan Zhai, Xuemei Liu, Minghua Deng, and Fengzhu Sun. Alignment-free sequence comparison based on next-generation sequencing reads. *Journal of Computational Biology*, 20:64–79, 2013.

[61] Stephanie J. Spielman and Claus O. Wilke. Pyvolve: A flexible python module for simulating sequences along phylogenies. *PLOS ONE*, 10(9):e0139047, 2015.

[62] Alexandros Stamatakis. RAxML-VI-HPC: maximum likelihood-based phylogenetic analyses with thousands of taxa and mixed models. *Bioinformatics*, 22:2688–2690, 2006.

[63] Alexandros Stamatakis. RAxML version 8: a tool for phylogenetic analysis and post-analysis of large phylogenies. *Bioinformatics*, 30:1312–1313, 2014.

[64] Mike Steel. Phylogenetic diversity and the greedy algorithm. *Systematic Biology*, 54:527–529, 2005.

[65] Hanno Teeling, Jost Waldmann, Thierry Lombardot, Margarete Bauer, and Frank Oliver Glöckner. Tetra: a web-service and a stand-alone program for the analysis and comparison of tetranucleotide usage patterns in dna sequences. *BMC Bioinformatics*, 5:163, 2004.

[66] Sharma V. Thankachan, Sriram P. Chockalingam, Yongchao Liu, and Ambujam Krishnan Srinivas Aluru. A greedy alignment-free distance estimator for phylogenetic inference. *BMC Bioinformatics*, 18:238, 2017.

[67] Sharma V. Thankachan, Sriram P. Chockalingam, Yongchao Liu, Alberto Apostolico, and Srinivas Aluru. ALFRED: a practical method for alignment-free distance computation. *Journal of Computational Biology*, 23:452–460, 2016.

[68] Igor Ulitsky, David Burstein, Tamir Tuller, and Benny Chor. The average common substring approach to phylogenomic reconstruction. *Journal of Computational Biology*, 13:336–350, 2006.

[69] Susana Vinga, Alexandra M. Carvalho, Alexandre P. Francisco, Luís M. S. Russo, and Jonas S. Almeida. Pattern matching through Chaos Game Representation: bridging numerical and discrete data structures for biological sequence analysis. *Algorithms for Molecular Biology*, 7:10, 2012.

[70] Lin Wan, Gesine Reinert, Fengzhu Sun, and Michael S Waterman. Alignment-free sequence comparison (II): theoretical power of comparison statistics. *Journal of Computational Biology*, 17:1467–1490, 2010.

[71] John H. Werren, Laura Baldo, and Michael E. Clark. Wolbachia: master manipulators of invertebrate biology. *Nature Reviews Microbiology*, 6:741 – 751, 2008.

[72] Huiguang Yi and Li Jin. Co-phylog: an assembly-free phylogenomic approach for closely related organisms. *Nucleic Acids Research*, 41:e75, 2013.

[73] Xiaofan Zhou, Xing-Xing Shen, Chris Todd Hittinger, and Antonis Rokas. Evaluating fast maximum likelihood-based phylogenetic programs using empirical phylogenomic data sets. *Molecular Biology and Evolution*, 35:486–503, 2018.

[74] Zhemin Zhou, Xiaomin Li, Bin Liu, Lothar Beutin, Jianguo Xu, Yan Ren, Lu Feng, Ruiting Lan, Peter R. Reeves, and Lei Wang. Derivation of *Escherichia coli* O157:H7 from Its O55:H7 Precursor. *PLOS ONE*, 5:e8700, 2010.

[75] Andrzej Zielezinski, Susana Vinga, Jonas Almeida, and Wojciech M. Karlowski. Alignment-free sequence comparison: benefits, applications, and tools. *Genome Biology*, 18:186, 2017.

Supplementary Material

Click here to access/download
Supplementary Material
protspam_supp_mat.pdf
