# Supplementary material for: Prot-SpaM: fast alignment-free phylogeny reconstruction based on whole-proteome sequences
Source: Gigascience. 2018 Dec 7;8(3):giy148. doi: 10.1093/gigascience/giy148 (PMC6436989; doi:10.1093/gigascience/giy148)
Supplement: Supplemental File [file giy148_supplemental_file.pdf]

Supplementary Material to

Prot-SpaM: Fast alignment-free phylogeny reconstruction based on whole-proteome sequences

Chris-Andre Leimeister, Jendrik Schellhorn,
Svenja Schöbel, Michael Gerth, Christoph Bleidorn,
Burkhard Morgenstern

1 The data set *Wolbachia II*

Supplementary Table 1

For the *Wolbachia* II dataset, we downloaded the proteomes for all available *Wolbachia* draft and fully assembled genomes.

Accession	Strain	Strain information
PRJNA42155	Ace	*Anaplasma centrale* str. Israel
PRJNA58577	Ama	*Anaplasma marginale* str. Florida
PRJNA57933	Ech	*Ehrlichia chaffeensis* str. Arkansas
PRJNA58245	Eru	*Ehrlichia ruminantium* str. Gardel
GCF_000242415.2	wAlbB	*Wolbachia endosymbiont of Aedes albopictus*
GCF_000167475.1	wAna	*Wolbachia endosymbiont of Drosophila ananassae*
GCF_000953315.1	wAu	*Wolbachia endosymbiont of Drosophila simulans wAu*
GCF_002318985.1	wAus	*Wolbachia endosymbiont of Plutella australiana*
GCF_000008385.1	wBm	*Wolbachia endosymbiont strain TRS of Brugia malayi*
GCF_000333775.1	wBol1	*Wolbachia* endosymbiont of *Hypolimnas bolina*
GCF_000829315.1	wCle	*Wolbachia endosymbiont of Cimex lectularius*
GCF_001648025.1	wDacA	*Wolbachia* endosymbiont of *Dactylopius coccus*
GCF_001648015.1	wDacB	*Wolbachia* endosymbiont of *Dactylopius coccus*
GCF_000331595.1	wDi	*Wolbachia endosymbiont of Diaphorina citri*
http://nematodes.org/genomes/dirofilaria_immitis/	wDim	*Wolbachia endosymbiont of Dirofilaria immitis*
GCA_000153585.1	wDwi	*Wolbachia* endosymbiont of *Drosophila willistoni* TSC#14030-0811.24
SRX512806	wFol	Wolbachia endosymbiont of *Folsomia candida*

PRJNA300838	wFol_pb	Wolbachia endosymbiont of *Folsomia candida* PacBio
GCF_000689175.1	wGmm	*Wolbachia endosymbiont of Glossina morsitans morsitans*
GCF_000376605.1	wHa	*Wolbachia* endosymbiont of *Drosophila simulans*
http://nematodes.org/genomes/litomosoides_sigmodontis/	wLs	*Wolbachia* endosymbiont of *Litomosoides sigmodontis*
GCF_000008025.1	wMel	*Wolbachia endosymbiont of Drosophila melanogaster*
GCF_000475015.1	wMelpop	*Wolbachia pipientis* wMelPop
GCF_001675785.1	wNfe	*Wolbachia* endosymbiont of *Nomada ferruginata*
GCF_001675695.1	wNfla	*Wolbachia* endosymbiont of *Nomada flava*
GCF_001675715.1	wNleu	*Wolbachia endosymbiont of Nomada leucophthalma*
GCF_000376585.1	wNo	*Wolbachia endosymbiont of Drosophila simulans*
GCF_001675775.1	wNpa	*Wolbachia endosymbiont of Nomada panzeri*
GCF_001266585.1	wObr	*Wolbachia endosymbiont of Operophtera brumata*
SRX512676	wOc	*Wolbachia* endosymbiont of *Osmia caerulescens*
GCF_000306885.1	wOo	*Wolbachia endosymbiont of Onchocerca ochengi*
GCF_000530755.1	wOv	*Wolbachia* endosymbiont of *Onchocerca volvulus* str. Cameroon
GCF_000156735.1	wPipJHB	*Wolbachia* endosymbiont of *Culex quinquefasciatus* JHB
GCF_000208785.1	wPipMol	*Wolbachia endosymbiont of Culex pipiens molestus*
GCF_000073005.1	wPipPel	*Wolbachia endosymbiont of Culex quinquefasciatus*
SRX766492	wPni	*Wolbachia* endosymbi

PRJNA300838	wFol_pb	Wolbachia endosymbiont of *Folsomia candida* PacBio
GCF_000689175.1	wGmm	*Wolbachia endosymbiont of Glossina morsitans morsitans*
GCF_000376605.1	wHa	*Wolbachia* endosymbiont of *Drosophila simulans*
http://nematodes.org/genomes/litomosoides_sigmodontis/	wLs	*Wolbachia* endosymbiont of *Litomosoides sigmodontis*
GCF_000008025.1	wMel	*Wolbachia endosymbiont of Drosophila melanogaster*
GCF_000475015.1	wMelpop	*Wolbachia pipientis* wMelPop
GCF_001675785.1	wNfe	*Wolbachia* endosymbiont of *Nomada ferruginata*
GCF_001675695.1	wNfla	*Wolbachia* endosymbiont of *Nomada flava*
GCF_001675715.1	wNleu	*Wolbachia endosymbiont of Nomada leucophthalma*
GCF_000376585.1	wNo	*Wolbachia endosymbiont of Drosophila simulans*
GCF_001675775.1	wNpa	*Wolbachia endosymbiont of Nomada panzeri*
GCF_001266585.1	wObr	*Wolbachia endosymbiont of Operophtera brumata*
SRX512676	wOc	*Wolbachia* endosymbiont of *Osmia caerulescens*
GCF_000306885.1	wOo	*Wolbachia endosymbiont of Onchocerca ochengi*
GCF_000530755.1	wOv	*Wolbachia* endosymbiont of *Onchocerca volvulus* str. Cameroon
GCF_000156735.1	wPipJHB	*Wolbachia* endosymbiont of *Culex quinquefasciatus* JHB
GCF_000208785.1	wPipMol	*Wolbachia endosymbiont of Culex pipiens molestus*
GCF_000073005.1	wPipPel	*Wolbachia endosymbiont of Culex quinquefasciatus*
SRX766492	wPni	*Wolbachia* endosymbi

2 Trees reconstructed for the set of 813 prokaryotes

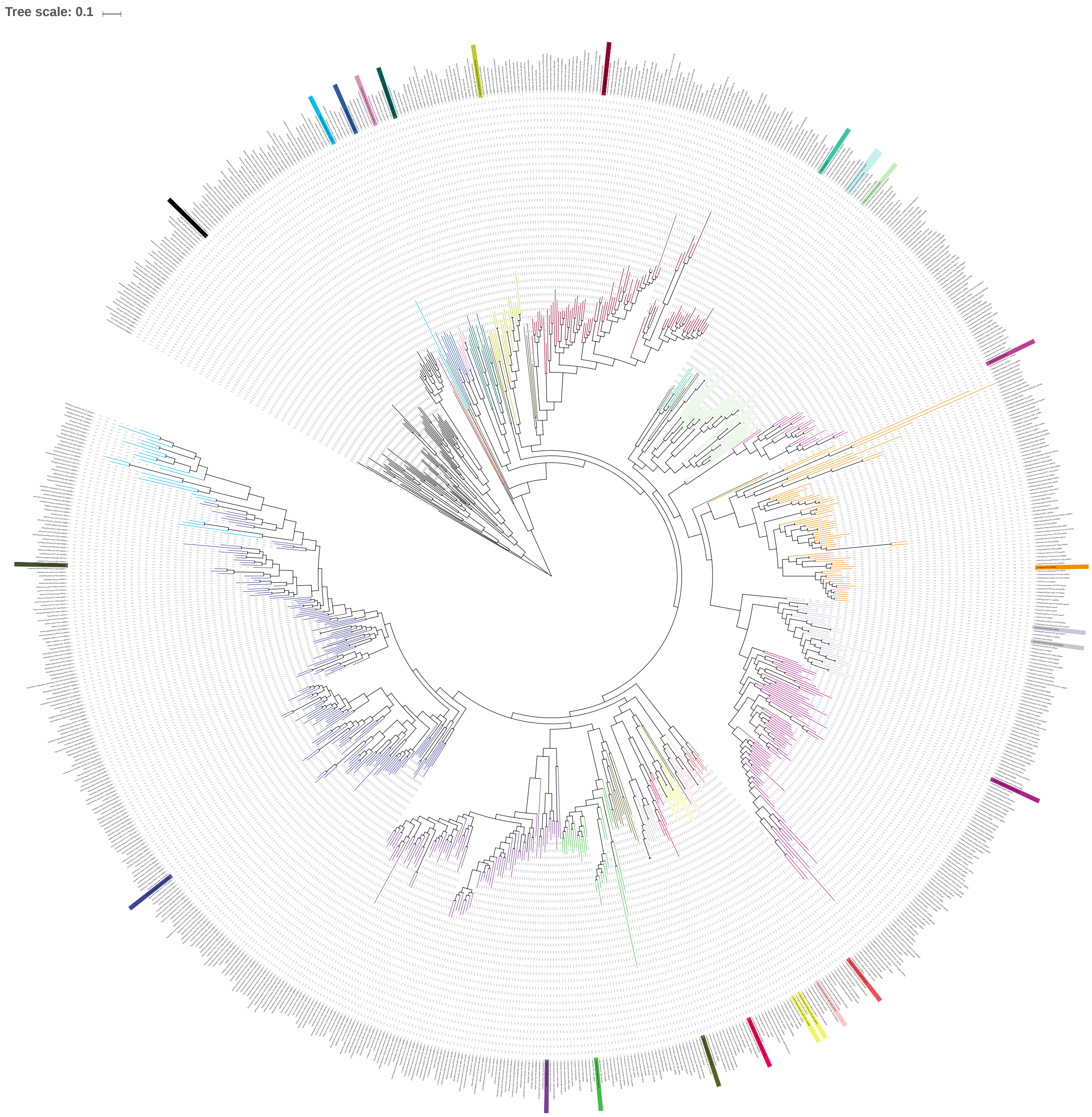

Figure 1: Maximum-Likelihood tree constructed by Lang *et al.* [3] based on a super alignment of 24 selected genes

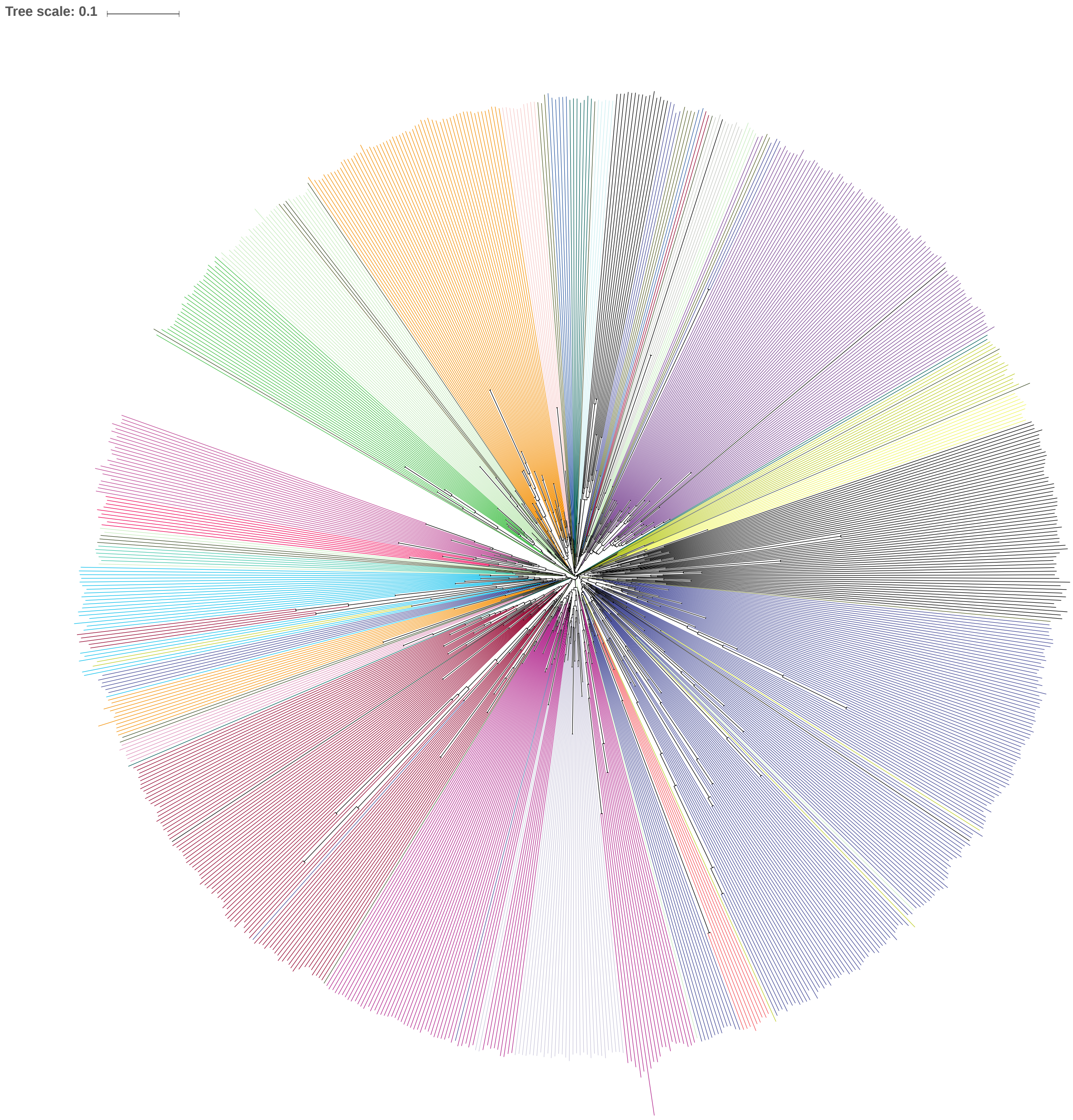

Figure 2: Tree constructed with *ACS* [7], for 813 taxa for which the proteomes are available in *GenBank* [1].

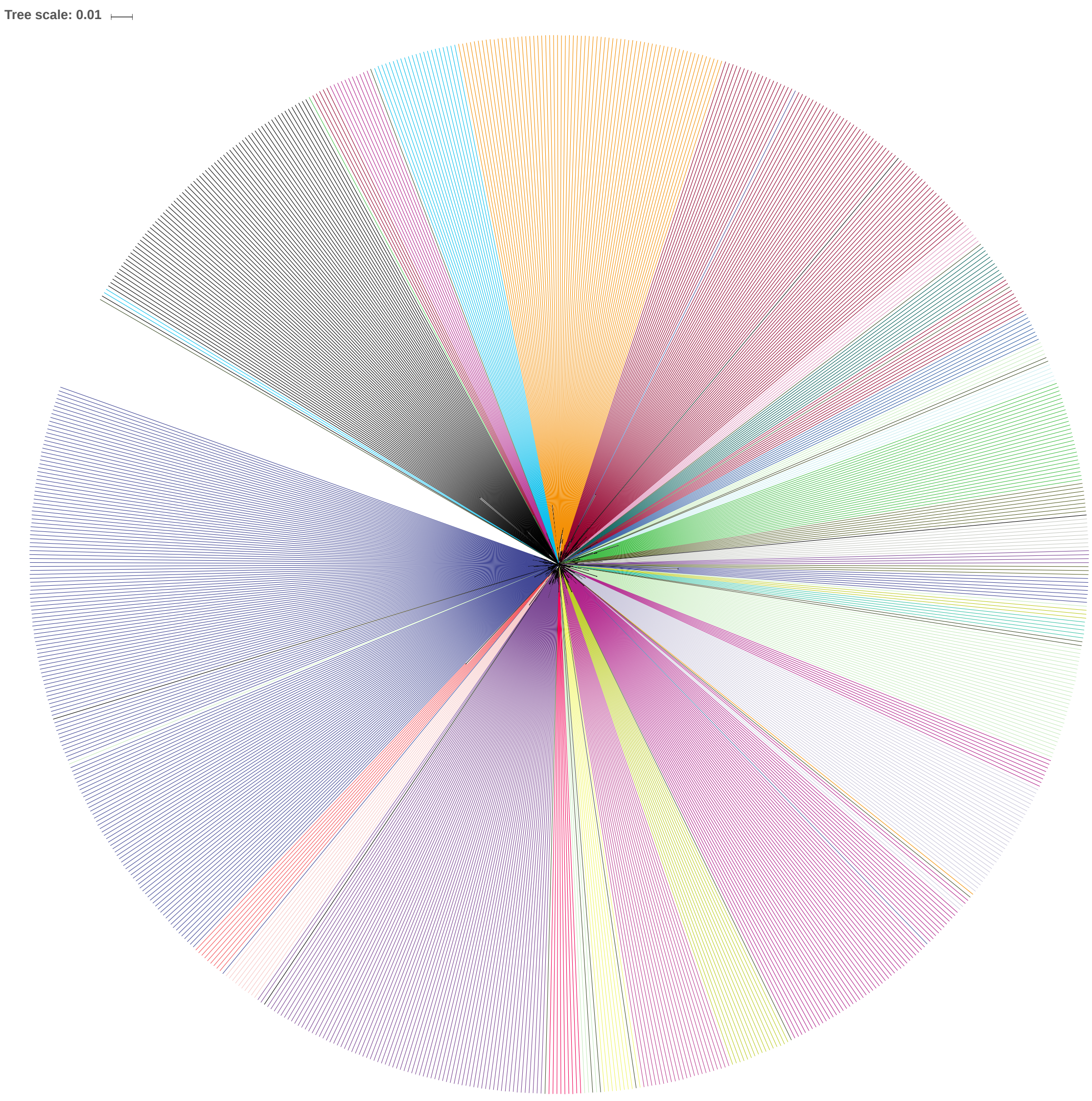

Figure 3: Tree constructed with *CVTree* [6], for 813 taxa for which the proteomes are available in GenBank

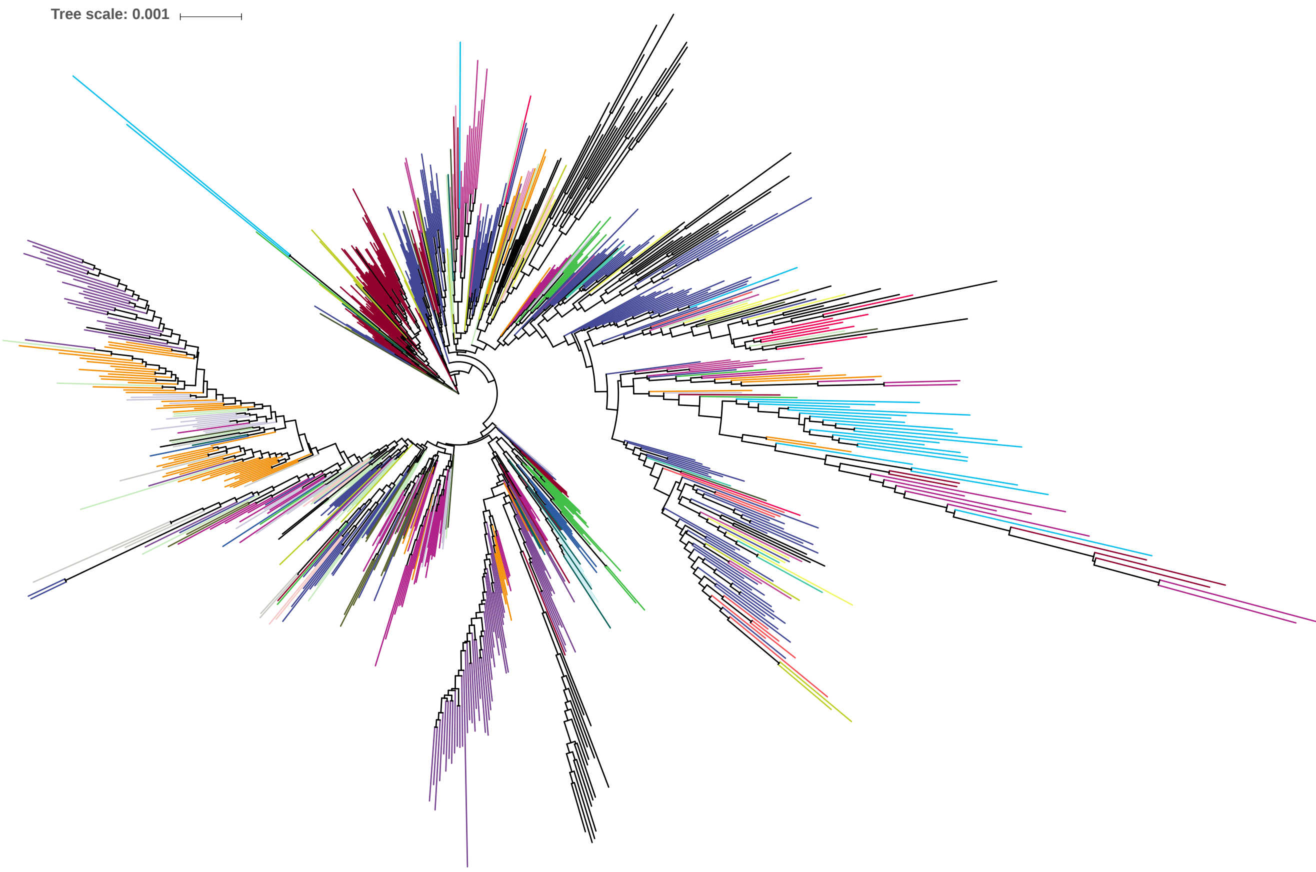

Figure 4: Tree constructed with *FFP* [2], for 813 taxa for which the proteomes are available in GenBank

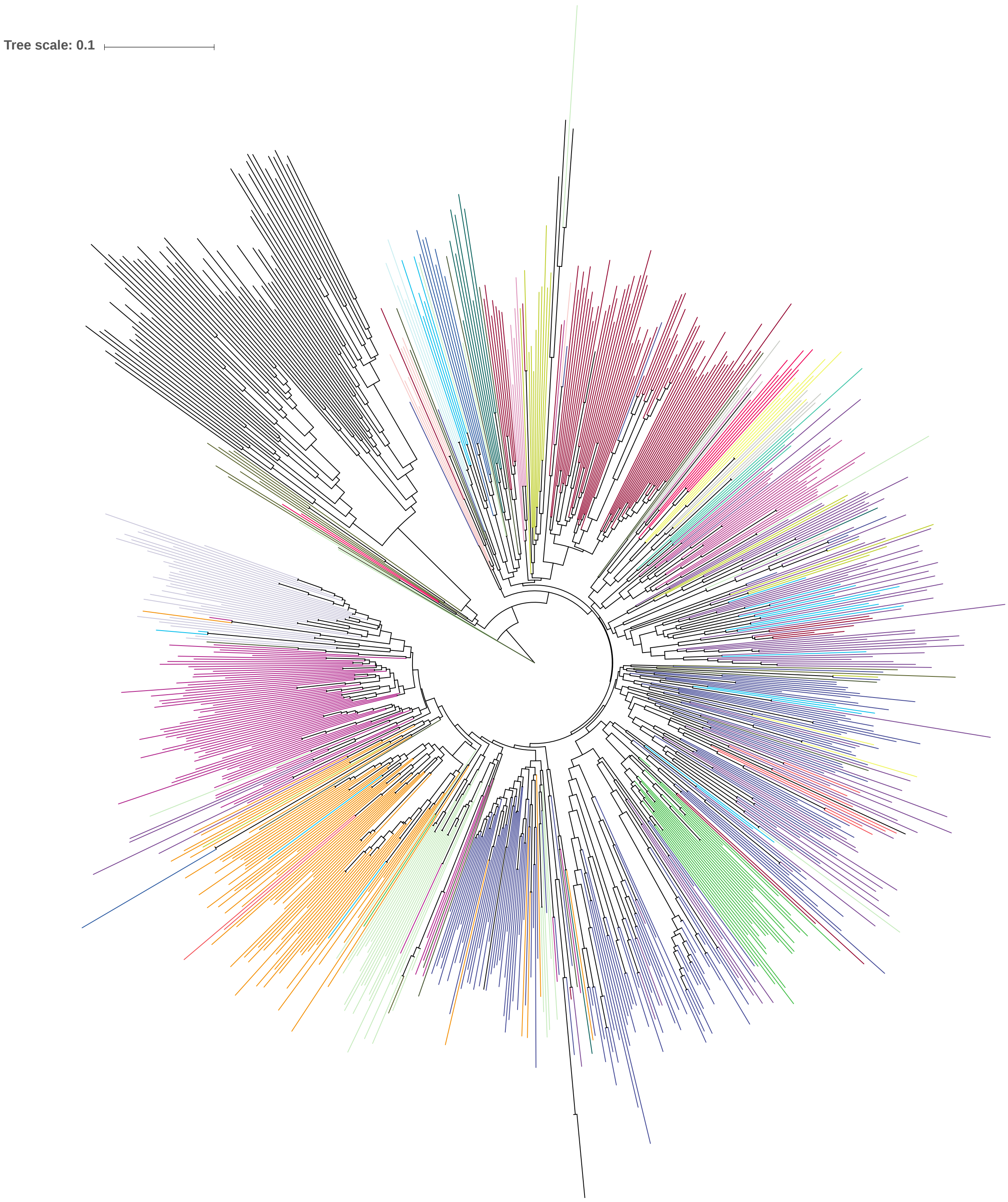

Figure 5: Tree reconstructed with *FSWM* [5] on the 813 whole-genome sequences.

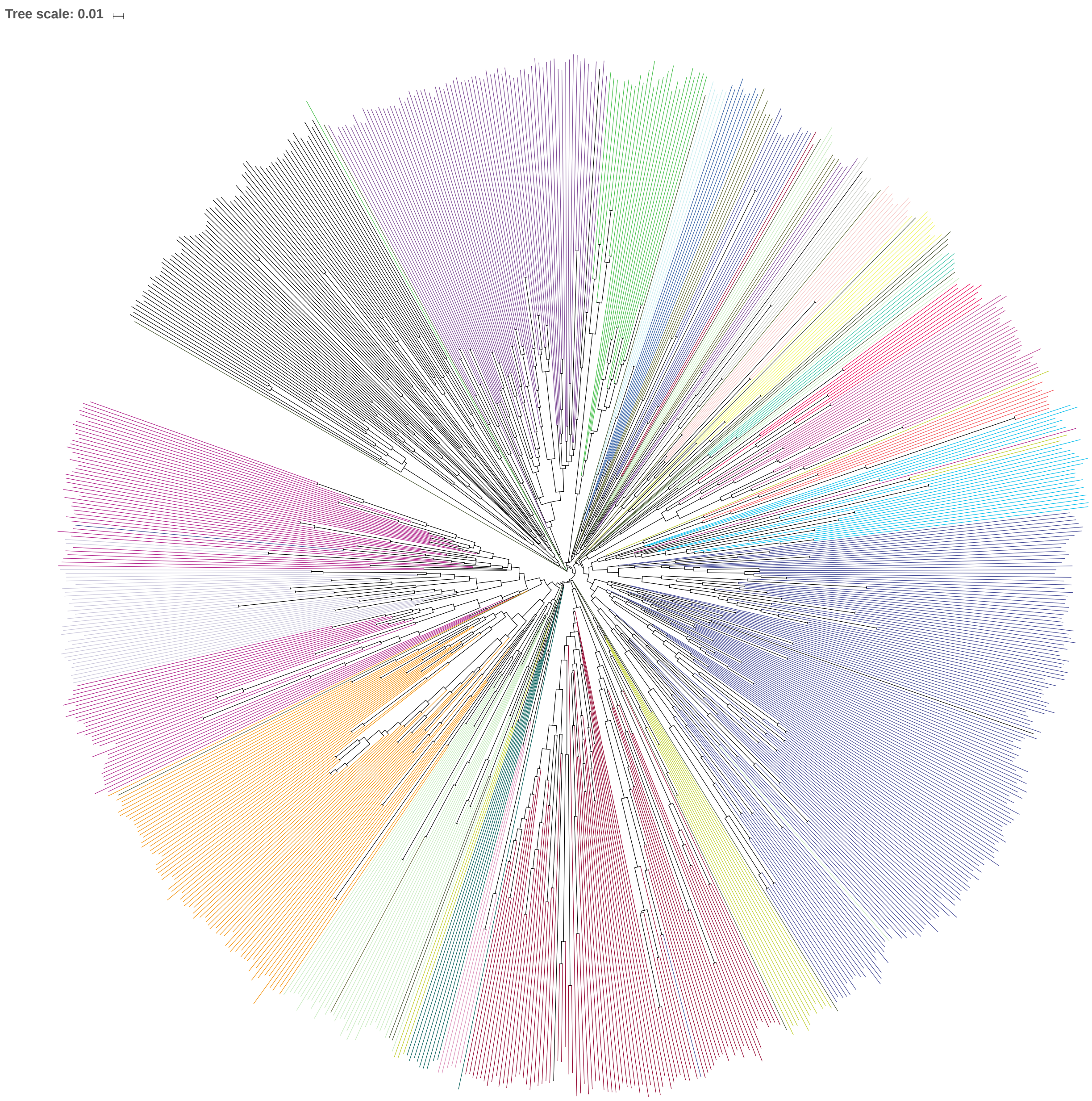

Figure 6: Tree constructed with *kmacs* [4], for 813 taxa for which the proteomes are available in *GenBank*

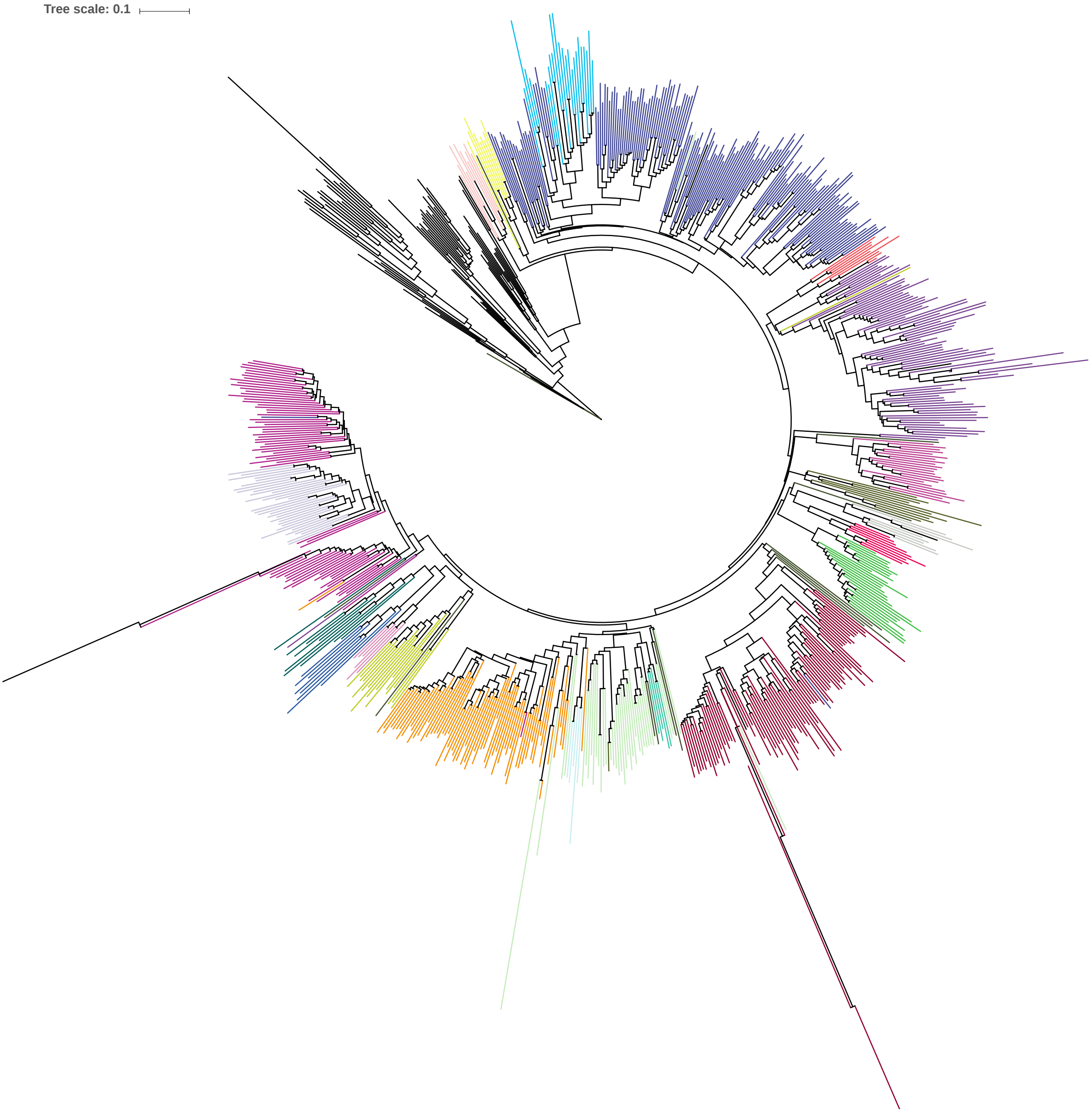

Figure 7: Tree constructed with *Prot-SpaM*, for 813 taxa for which the proteomes are available in *GenBank*

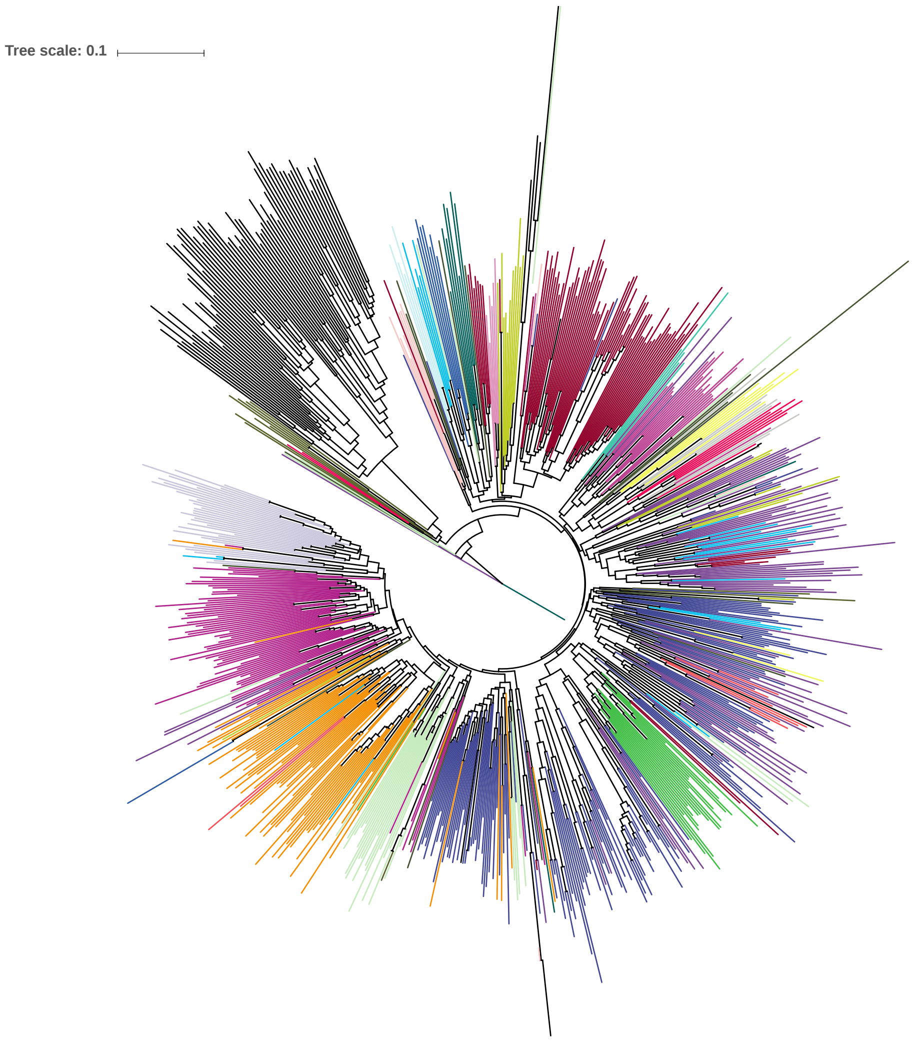

Figure 8: Tree reconstructed with *FSWM* [5] on the 841 whole-genome sequences.

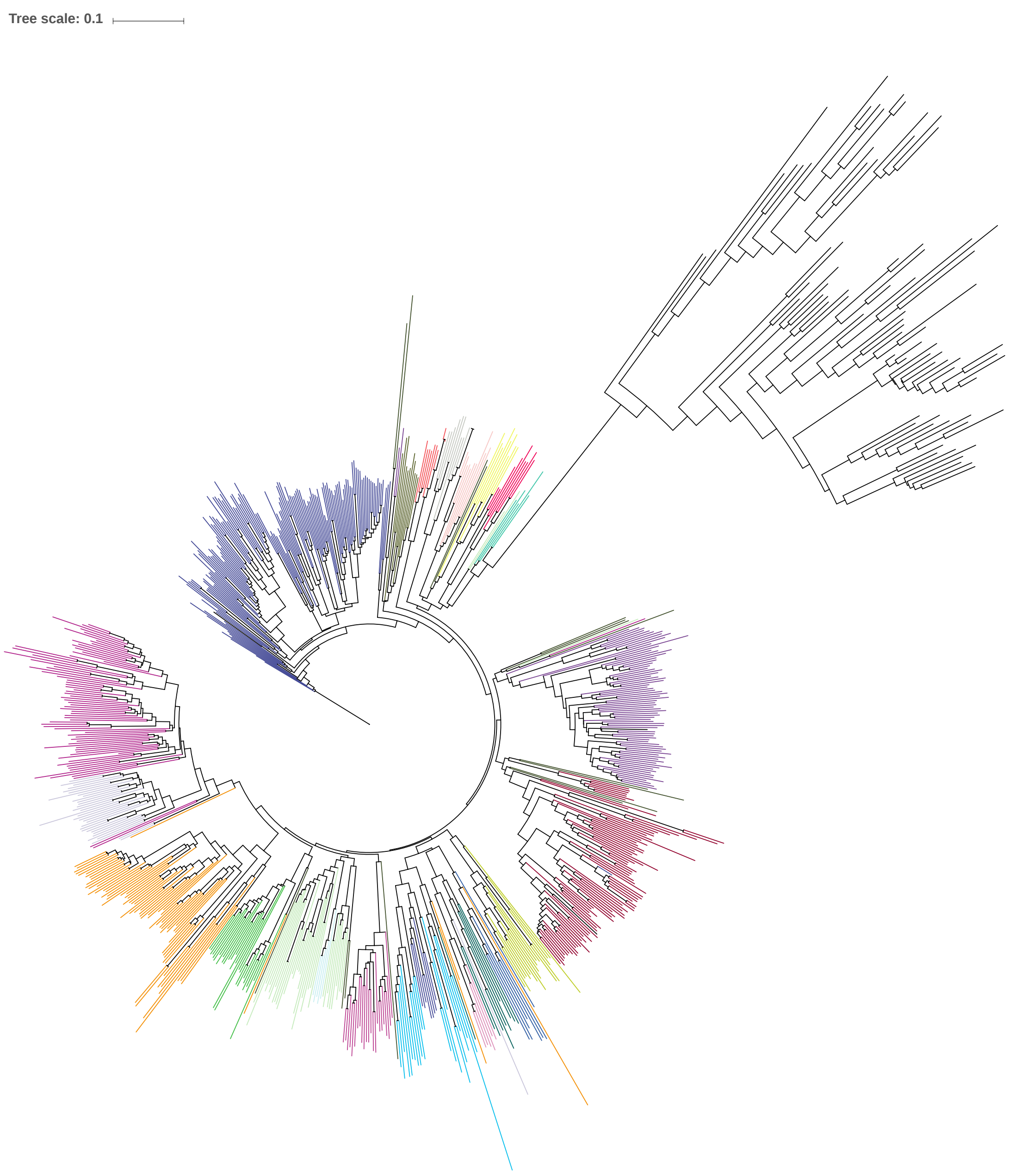

Figure 9: Tree constructed with *Prot-SpaM* on the proteins corresponding to the 24 genes selected by Lang *et al.* [3]

References

[1] Dennis A Benson, Mark Cavanaugh, Karen Clark, Ilene Karsch-Mizrachi, James Ostell, Kim D Pruitt, and Eric W Sayers. Genbank. *Nucleic Acids Research*, 46(D1):D41–D47, 2018.

[2] Se-Ran Jun, Gregory E. Sims, Guohong A. Wu, and Sung-Hou Kim. Whole-proteome phylogeny of prokaryotes by feature frequency profiles: An alignment-free method with optimal feature resolution. *Proceedings of the National Academy of Sciences*, 107:133–138, 2010.

[3] Jenna Morgan Lang, Aaron E. Darling, and Jonathan A. Eisen. Phylogeny of bacterial and archaeal genomes using conserved genes: Supertrees and supermatrices. *PLOS ONE*, 8:e62510, 2013.

[4] Chris-André Leimeister and Burkhard Morgenstern. *kmacs*: the *k*-mismatch average common substring approach to alignment-free sequence comparison. *Bioinformatics*, 30:2000–2008, 2014.

[5] Chris-André Leimeister, Salma Sohrabi-Jahromi, and Burkhard Morgenstern. Fast and accurate phylogeny reconstruction using filtered spaced-word matches. *Bioinformatics*, 33:971–979, 2017.

[6] Ji Qi, Hong Luo, and Bailin Hao. CVTree: a phylogenetic tree reconstruction tool based on whole genomes. *Nucleic Acids Research*, 32(suppl 2):W45–W47, 2004.

[7] Igor Ulitsky, David Burstein, Tamir Tuller, and Benny Chor. The average common substring approach to phylogenomic reconstruction. *Journal of Computational Biology*, 13:336–350, 2006.
