# Supplementary material for: Prot-SpaM: fast alignment-free phylogeny reconstruction based on whole-proteome sequences
Source: Gigascience. 2018 Dec 7;8(3):giy148. doi: 10.1093/gigascience/giy148 (PMC6436989; doi:10.1093/gigascience/giy148)
Supplement: Response_to_Reviewer_Comments_Original_Submission.pdf [file giy148_response_to_reviewer_comments_original_submission.pdf]

Author's Response To Reviewer Comments

Close

Reviewer #1:

REVIEWER:
All defied terms and parameters should have consistency. There are some terms which appear both in italic font and simple text font, \textit{i.e.} \textit{homologous}, \textit{spaced-word matches} and \textit{don't-care positions}. Also some words in page 3 are underlined.

REPLY:
As usual, some words are in italics to emphasize them, so if a word is in italics at some point, it does not need to be in italices in other parts of the manuscript. "i.e." is in italics throughout the paper, as it should be. We partially underlined some words to indicate where the acronym "Prot-SpaM" comes from. We don't insist on this; if that's not in line with the journal style, we will remove the underlining.

REVIEWER:
Cite Kimura model and PAM distance in line 26 of page 5 should be referenced.
MegaByte shoulb be written as "MB" not "mb".

REPLY:
Done.

===

Reviewer #3:

Minor Comments:

REVIEWER:
-. References are not ordered.

REPLY:
We used \bibliographystyle{plain}; therefore references are currently in alphabetic order. In case the paper gets accepted, we will of course use the bib style that is used by the publisher.

REVIEWER:
-. On page 4, the cartoon for explaining of concept of spaced-word matches is not correct.

REPLY:
The cartoon is correct. It seems that our previous definitions were not clear enough, and there was a misunderstanding about our concept of "spaced words". We therefore revised the definitions and tried to explain the meaning of "spaced words" and "spaced word matches" more clearly.

REVIEWER:

-. Figure 2 legend says ProtFSWM not Prot-SpaM

REPLY:
Done

REVIEWER:
-. On Page 5, authors checked if spaced-words matches in the two compared sequences are one-to-one mapping. Please describe how one-to-one mapping from one-to-multiple or multiple-to-multiple was defined?

REPLY:
See above. Our definition of "spaced-word matches" was a bit sloppy, this may have led to some misunderstandings. We now give formally more correct definitions of "spaced words" and "spaced-word matches". Also, we revised the paragraph on "one-to-one mapping" and used a more precise definition. We hope, this helps to clarify this point.

REVIEWER:
-. On page 5, authors used Kimura model to approximate PAM distance. Please put a reference for Kimura model. If there are parameters involved in Kimura model, please describe how to estimate those parameters.

REPLY:
Done.

REVIEWER:
-. Authors used BLOSUM62 when to distinguish homologous spaced-word matches with random spaced-word matches. But, authors approximated PAM distance between protein sequences. Please describe rational different substitution matrices used for different purposes?

REPLY:
Thanks for pointing this out. We added a paragraph to clarify this point (end of the "Methods" section).

REVIEWER:
-. For Table 1, Table 2, Table 3, Figure 2, and Figure 5, please describe which length of K-mer was used for FFP method.

REPLY:
We used the default value which is k=4.

REVIEWER:
-. One page 8, "One interpretation is that misleading signal stemming from recombination events between Wolbachia strains is less problematic for alignment-free analysis then a reduction in he dataset size." <= please revise the sentence.

REPLY:
We removed this sentence from the "Results" section and added two new paragraphs to the "Discussion" section, where this point is discussed in detail. We hope that this clarifies the issue.

REVIEWER:
-. On page 9, "we applied Prot-SpaM to all available protein sequences from these 813 taxa. In addition, we ran Prot-SpaM on the protein sequences encoded by the 24 marker genes from Lang et al." <= For application of Prot-SpaM to two different type of datasets, were same selected spaced-word matches and same patterns used?

REPLY:
We used the same set of parameter values in all the test runs, i.e. a pattern length of 46, a weight of 6, and 5 different patterns. However, as mentioned in the manuscript, we are using probabilistic algorithm to calculated sets of patterns. Therefore, we generally have not the same pattern sets in different program runs. We made the pattern sets that we used in the test runs available, so the results can be reproduced (see our reply to the last question of Reviewer #4)

REVIEWER:
-. For Table 3, what is the unit of computational time (seconds/minutes/hours)?

REPLY:
This is mentioned in the legend: "runtime in seconds".

REVIEWER:
Major Comments:
-. Since authors describes a new alignment-free method for whole-proteome phylogeny, please include the existing alignment-free method developed specifically for whole proteome phylogeny ("Whole-proteome phylogeny of prokaryotes by feature frequency profiles: An alignment-free method with optimal feature resolution" by Jun et al, PANS 2010; 107:133-138") for the comparison.

REPLY:
We evaluate FFP in our study (e.g. Table 1 and 2) cite a paper on FFP. We now added a reference to Jun et al, 2010.

REVIEWER:
-. No discussion found about Table 3 which summarized computational time for Prot-SpaM, and other alignment-free methods.

REPLY:
We now discuss the run time in detail in the "Discussion" section.

REVIEWER:
-. Authors claims that Prot-SpaM generates more statistically meaningful trees than other alignment-free methods. But, I don't see description of statistical confidence on internal nodes. Please describe how to impose statistical confidence on internal nodes of trees generated by Prot-SpaM.

REPLY:
This may be a misunderstanding. We emphasize that our approach estimates the *number of substitutions* per sequence position based on a stochastic model of molecular evolution. So this is different from previous alignment-free approaches that output rather rough measures

of sequence similarity/dissimilarity. But statistical confidence for specific nodes in a tree is a completely different thing. We are planning to work on this topic in the context of our approach, but this will be an entirely new project. It is not possible to do this within the current project. Therefore, we currently do not have confidence values for internal nodes.

REVIEWER:
-. On page 9, "There are some differences within the clades, though, that should be further investigated." <= please at least provide information of RF and Branch score distance between four trees in Figure 4.

REPLY:
The RF distances are given in Table 1. As explained below, we preferred to omit the "branch scores" altogether, since they only caused confusion (and branch scores are not commonly used to evaluate alignment-free methods, for good reasons).

REVIEWER:
-. In Figure 3, Did Prot-SpaM segregate E.coli from Shigella? The paper "Insights from 20 years of bacteria genome sequencing" published in Funct Integr Genomics, 2015; 15:141-161 (https://www.ncbi.nlm.nih.gov/pmc/articles/PMC4361730/) showed a tree which segregated E.coli from Shigella clearly using the method described in "Whole-proteome phylogeny of prokaryotes by feature frequency profiles: An alignment-free method with optimal feature resolution" by Jun et al, PANS 2010; 107:133-138. Please discuss about which alignment-free methods including Jun et al's method have a capability of segregating E.coli from Shigella.

Insights from 20 years of bacterial genome sequencing
www.ncbi.nlm.nih.gov
Since the first two complete bacterial genome sequences were published in 1995, the science of bacteria has dramatically changed. Using third-generation DNA sequencing, it is possible to completely sequence a bacterial genome in a few hours and identify ...

REPLY:
We used a reference tree from Zhou (2010). In this tree, Shigella is not clearly separated from E.coli. In fact, many authors argue that Shigella is not monophyletic, so it should not be separated in the tree. But since our paper is not on the evolution of Shigella, we do not want to comment on this, so we just compare our trees to a trusted reference tree (as is common practice in alignment-free papers).

REVIEWER:
-. On page 8, authors described analysis results of Wolbachia strains without presenting a tree. Please provide the tree with annotations according to discussion to support the analysis results. In the subsection of Wolbachia, authors described how to generate an alignment-based tree with Wolbachia II dataset. But, there is no discussion found about comparison results with other alignment-free methods for Wolbachia II dataset.

REPLY:
We now included a figure with the Wolbachia tree produced by our approach plus the reference tree. The results are now discussed.

REVIEWER:

-. Selected spaced-word matches and patterns are the most important factors for the method, Prot-SpaM. It seems that a length of l=46, a weight of w = 6, 40 don't-care positions and five patterns were used through the study. I don't see how authors reached these parameter values. Please describe the optimization procedure for these parameters, l, w, five patterns. For these optimized values, does selected spaced-word matches mean spaced-word matches with scores >0? Please describe meaning of 'selected' in "selected spaced-word matches".

REPLY:
Yes, this was missing in the initial submission. We used those parameter values since they worked well on a broad set of test data. To clarify this, we now added two tables with runtime and RF distances for different parameter values. Also, we clarified what "selected spaced-word matches" means. (It means score > 0 and one-to-one mapping of spaced-word occurrences).

REVIEWER:
Second, please clarify whether selected spaced-word matches with a length of l=46, a weight of w = 6, 40 don't-care positions, and five patterns fixed were for any dataset (simulated, prokaryotic, eukaryotic proteomes, protein sequences)? Please provide sets of selected string-word matches and patterns in Supplementary.

REPLY:
Yes, we used these parameter values in all test runs. It's not clear to us, however, what the reviewer means with "provide sets of selected string-word matches and patterns". We now published all pattern sets that we used, so the results can be reproduced.

REVIEWER:
Third, please describe computational time for defining selected spaced-word matches and patterns.

REPLY:
Patterns are calculated with rasbhari, the runtime of this program is described in detail in the original publication (Hahn et al., 2016). The runtime for "defining selected spaced-word matches" is difficult to specify, since selecting ("defining") spaeced-word matches and calculating the number of mismatches per position from the selected spaced-word matches is done in one single step. It's difficult to separate this in the runtime analysis. We think, what really matters is the overall runtime of the program, not so much the runtime for the single steps in of the program.

REVIEWER:
Fourth, Prot-SpaM uses only selected spaced-word matches which indicates fraction of proteomes are compared instead of whole proteomes when being built phylogenies. Please describe what fraction of proteomes on average by Prot-SpaM were being used for datasets discussed in the manuscript.

REPLY:
It is not entirely clear to us what "fraction of proteomes .. used for datasets" means. We used the full proteomes in our analyses. Does the reviewer want to know which fraction of the proteomes is covered by SW matches? We now added some data on the number of spaced-word matches that our program uses, from two extreme cases: one where a large number of

sw-matches is selected (closely related sequences) and one with a small number of sw-matches (distantly related sequences).

REVIEWER:
-. In comparing Table 1 (RF distance) with Table 2 (Branch score distance), since Prot-SpaM captures evolutionary distance between two sequences unlike other alignment-free methods, Prot-SpaM should perform better than other alignment-free methods against alignment-based reference trees by Brach score distance. But, for example, trees by CVTree method were closer to alignment-based reference trees than trees by Prot-SpaM with branch-score distance for some datasets even though CVtree doesn't capture evolutionary distance at all. Please discuss about this issue.

REPLY:
For various reasons, the "branch score" distances caused confusion in our study, see other reviewer comments and our reply to them. Therefore, we prefer to omit the branch scores and to restrict ourselves to the RF distances which are commonly used in the literature (all other alignment-free papers use RF distances, we don't know any paper where branch scores are used to evaluate alignment-free approaches). In most examples, Prot-SpaM performed better than other alignment-free methods, and we now discuss this point in the "Discussion".

REVIEWER:
-. In the manuscript, validation step was solely dependent on alignment-based reference trees which sounds like that authors tried to develop an alignment-free method which produces trees most resemblance to alignment-based tree. For example, a reference tree of 813 prokaryotes was based on 24 marker genes and was found to be very similar to be 16S rRNA-based tree. Then, according to the validation procedure, Prot-SpaM tried to prove that the method produces a tree most resemblance to a 16S rRNA-based tree which does not require orthologous analysis and might not be computationally inferior to Prot-SpaM. Furthermore, even though Prot-SpaM pairwise distance captures evolutionary distance, the distances on the Prot-SpaM tree cannot be interpreted with substitution rates since distance-based methods are only applicable. To investigate other advantages of Prot-SpaM, the method needs to be examined over taxonomic classification in comparison with other alignment-free methods
since taxonomic classification captures evolutionary information. Please examine a capability of taxonomic classification for the datasets discussed in the manuscript at least at the species level in comparison with other alignment-free methods.

REPLY:
Alignment-free approaches are certainly computationally more efficient than 16S rRNA-based approaches where one first has to identify these sequences and then construct an alignment. And it's not quite clear to us what the reviewer means with "taxonomic classification". We do this by comparing phylogenetic trees to reference trees, and this is what all papers on alignment-free methods are doing to evaluate new tools.

REVIEWER:
-. The following error messages occurred when compiling the code downloaded from "https://github.com/jschellh/ProtSpaM" by 'make'. It seems that sysinfo.h is not provided.

```
mkdir -p obj
```

```
g++ -fopenmp -c -Wall -std=c++11 -I ./include main.cpp -o obj/main.o
In file included from ./include/speedsens.hpp:6:0,
from ./include/rasbcomp.hpp:9,
from ./include/rasbhari.hpp:8,
from ./include/rasbimp.hpp:4,
from main.cpp:26:
./include/sensmem.hpp:7:25: fatal error: sys/sysinfo.h: No such file or directory
#include "sys/sysinfo.h"
^
compilation terminated.
make: *** [obj/main.o] Error 1
```

REPLY:
The error message seems to be produced by the program rasbhari, which our software calls. It seems that this error occurs if one tries to run the program on Windows. The current version of the program, however, is designed to run on Linux. We now made clear that our program should be used under Unix/Linux.

==
==

Reviewer #4:

REVIEWER:
The paper presents Prot-SpaM, a method to construct phylogenetic trees using proteome data based on the alignment-free spaced-word approach. The proposed method estimates pairwise distances for the set of sequences by computing the mapping of spaced words. The authors evaluate their approach using simulated data and also demonstrate the approach using for different real datasets, ranging from microbial data to plant proteomes. The authors also provide a rigorous method to select the threshold parameter using the spaced-word match histogram. Using the experiments using simulated data, they show Prot-SpaM proves a better approximation w.r.t to the expected distance computed by the Kimura model. The authors have made the software freely available at github, and is easy to build and run on a typical linux machine.

REPLY:
Thank you.

REVIEWER:
1. The key difficulty when using spaced-words approach in practice is the selection of the key parameters (a) pattern weight (b) number of don't care positions, (c) number of patterns and (d) minimum threshold. The paper includes a very good discussion on the selection of minimum threshold using spaced-word histogram. However, there is no guidance on how the other parameters can be selected. Even though all the experiments use the default parameters, a brief discussion on the effect of these parameters can be useful for practitioners.

REPLY:
We agree, this was missing in the previous version of the manuscript. We now added two tables with different parameter values to clarify why we are using our default values (see

also reply to Reviewer 3)

REVIEWER:
2. The paper mentions that program uses 5 patterns, by default. Is there any reason for this default ?

REPLY:
With the multiple-pattern option, the quality of the results on our benchmark data was the same as with the single-pattern approach, in view of the RF distance. However, the estimated distances are more stable with the multiple-pattern approach. We added a figure to show this. We clarified this point in the manuscript; for large data sets users can reduce the number of patterns to speed-up the program.

REVIEWER:
3. The paper mentions that the pattern set is identified using a probabilistic algorithm, rasbhari, and hence, for two different runs with the same parameters can result in two different pattern sets. How does this affect the output trees ? In the experiments with real datasets, the authors provide a single RF distance in the Table 1. How does the RF distance varies w.r.t the probabilistic pattern set selection ?

REPLY:
This is a good point. For the previous manuscript, we did a *single* program run on each data set. Now, we did 100 program runs for each data set and report the average RF distances and standard deviations (except for the large bacterial data set. Here, we did only one program run because of the long run time for this data set).

REVIEWER:
4. In the descriptions of the algorithm (specifically in the third and sixth paragraphs of the introduction), the authors use the word 'alignment' to specify the spaced word pattern matches. I think this use of alignment description here is a bit confusing because pattern matching with spaced words is scored only in terms of the hamming distance.

REPLY:
We think, aligning two equal-length substrings without gaps is still an "alignment", even if this is a trivial sort of alignment (The term "gap-free alignment" is often used in this context in the literature). Therefore we'd prefer to leave the term "alignment" as it is. (BTW we are not using the Hamming distances, but the sum of scores of "aligned" amino-acid residues).

REVIEWER:
5. For experiments with simulated data (Section 3.1), the authors mention that they evaluated 1000 sequence pairs for each distance data. They also mention that they ran with both default parameters and with only one pattern. Does the error bars (standard deviations) plotted in figure 2 include results from both runs or only the default runs ?

REPLY:
The figure shows the results for the default parameter values, as is mentioned in the legend. We now added a figure with results where we used only one pattern and compared this to the default (5 patterns). This shows, that the results are more stable with multiple patterns.

REVIEWER:

6. For the microbial phylogeny, the authors mention that the tree obtained with Prot-SpaM contains essentially the same clades. It is very difficult to see from Figure 3, in terms of the clades, how different the tree constructed by kmacs or ACS from reference tree, which has lower RF distances compared to Prot-SpaM ?

REPLY:
Does the reviewer mean Figure 4 of the bioRxiv preprint (now Figure 5 of the revised manuscript)? Because of the large number of taxa, it is indeed difficult to compare these trees by looking at them. We added larger figures of the trees calculated by our and competing methods to the supplementary material.

REVIEWER:
7. While reporting the runtime, it is instructive to provide the type of machine the software were run. Also, it seems the implementation available at https://github.com/jschellh/ProtSpaM has multi-threaded capability, but the table 3 doesn't mention if it was run using single thread or multiple threads.

REPLY:
Done (added to first paragraph of "Resuts" section).

REVIEWER:
8. While the paper says that the use 5 patterns with w = 6 as default, the software in github appears to use only one pattern with w = 8 for default. Is there a reason for this discrepancy ?

REPLY:
Thank you, this was an error, we corrected this on github.

REVIEWER:
I also reviewed the supplementary data and the documentation seems sufficient to reproduce the results. Since there is probabilistic algorithm to select of the pattern sets, it would be useful to have a few seed patterns for the simulated data, if possible. Also, it can be useful for future research if the authors can provide all the reference trees along with the supplementary data.

REPLY:
We made the pattern sets available that were used in the test runs. Also, we added the following options to our software: (a) output the pattern set used in a program run to a separate file. (b) Use a user-defined pattern set instead of the pattern set generated by rasbhari. This way, it is possible too reproduce program runs with exactly the same pattern sets (which was indeed somewhat difficult with the previous version of the program where in each program run a new pattern set was used that was generated probabilistically)

===

Reviewer #5: Genenal comments:

REVIEWER:
In this manuscript, Leimeister et al. present a novel tool for alignment-free phylogeny

reconstruction from protein sequences.
Overall, the manuscript is well structured, written in clear language and is easy to follow. Tables and plots are used adequately to represent experimental results (see some specific suggestions below).

REPLY:
Thank you.

REVIEWER:
The presented method appears to be a rather minor modification of the existing FSWM tool (Leimeister et al. 2017, Bioinformatics). In particular, the only differnces seem to be: (1) using a different input alphabet (aminoacids) and thus a different scoring matrix (BLOSUM62), (2) using multiple patterns, and (3) using Kimura distance correction. Given that optimizations (2) and (3) can also be applied for genomic/DNA sequences, is it really justified to implement these minor modifications as a separate software tool? Arguably, having a single tool which can handle both DNA and protein sequences would be more convenient for the users while also simplifying software maintenance.

REPLY:
Due to technical issues, it is not trivial to have both programs in one program code, just with different options for DNA and proteins. One thing is, for example, the different alphabet size for DNA and proteins. But we fully agree that it would be preferable to have one single piece of software, and we will see if we can achieve this in the future.

REVIEWER:
Despite limited methodological novelty, ProtSPAM could be still a valuable addition to the field if it can achieve significant improvements over existing methods in terms of the tree reconstruction accuracy and/or speed. Unfortunately, presented experimental results cannot convincingly demonstrate this. When compared to FSWM, ProtSPAM is significantly more accurate only on 1 dataset out of 6 (Tables 1 and 2). And even on this dataset (813 prokaryotes), ProtSPAM showed higher topological error (RF distance) than 3 competing methods (CVTree, kmacs and ACS). However, even the best method (kmacs) inferred a tree which is very dissimilar to the reference (relative RF distance rRF=0.54). Therefore, differences between e.g., kmacs (rRF=0.54), ProtSPAM (rRF=0.63) and FSWM (rRF=0.83) are remarkable but less important, since all methods can be considered very inaccurate. Moreover, these results can be confounded by the instability of the reference tree itself, which is quite common
for large trees inferred from a limited set of genes (see Figure 6 in Lang et al. 2013). Additionaly, it is not clear whether we can expect high level of similarity between trees built from whole proteomes and ribosome proteins, since the latter one is in turn quite different from the 16S tree (rRF=~0.5, same figure).

Given the above inherent problems with empirical datasets, I suggest to complement ProtSPAM evaluation with benchmarks on simulated data. In addition to the known reference tree, simulation allows to freely variate number of taxa, genome/proteome size and substitution rates, and thereby to compare performance of alternative methods more systematically. I can also recommend (Zhou 2017, https://academic.oup.com/mbe/article/35/2/486/4644721#113627412) for a decent set of thoroughly analyzed empirical phylogenomic datasets.

REPLY:
(a) If simulated data are used, the produced distances can be evaluated *directly*, this is what we have done. The disadvantage of simulated data is that they depend on the parameters used in the simulation. Therefore, we also added real data, as is common practice. On the real data, distances cannot be evaluated directly, so the evaluation is done *indirectly* by tree inference. We don't see why we should evaluate our program *indirectly*, considering the produced trees, on simulated data, where a direct evaluation of the distances is possible.
(b) Thanks for making us aware of the Zhou paper, this looks very useful. We now added one challenging benchmark set from this paper, namely the metazoan data set from Borowiec et al.
(c) We made some mistakes in the previous evaluation which are corrected now. The table with the RF distances shows that our method is superior to other alignment-free methods on whole proteomes. It is only slightly outperformed by other methods on selected proteins in the Wolbachia I data sets, we now address this point in the "Discussion".

REVIEWER:
In terms of computational speed, ProtSPAM seems to drastically outperform FSWM and has runtimes comparable to other methods which operate on protein sequences. Although >100x runtime difference between two highly similar methods looks somewhat surprising (see below), if this speedup is real and consistent, it could be a major argument in favor of ProtSPAM.

REPLY:
Thanks. Please note that FSWM runs on whole genomes while Prot-SpaM runs on proteomes. Since for eukaryotes, proteomes are much smaller, this is the reason why Prot-SpaM is faster. We discuss this now in the "Discussion".

REVIEWER:
In summary, the authors should try to make it clear under which conditions ProtSPAM outperforms other methods (high sequence divergence? large trees? large genomes?), and support this claim by convincing experimental results.

REPLY:
The focus of this paper is on the new algorithm to estimate distances from whole proteome sequences. We used various benchmark sets to evaluate our approach, as is common in the field, and we discuss runtime and accuracy in different situations. We now also discuss in more detail where our approach is superior to other methods and why we think this is the case. In short: it is not necessary to select orthologous genes/proteins first. However, we did not perform a comprehensive study to see under which conditions which method may perform better or worse. This would be beyond the scope of the present study.

REVIEWER:
- Figure 1: In my opinion, plots for empirical datasets (C and D) do not quite support authors' choise of the alignment score cutoff (T=0). Furthermore, the optimal cutoff value will probably depend on k-mer length/weight, as well as on the substitution matrix selected. It appears more reasonable to use an adaptive cutoff T, derived from the spamogram for the particular dataset under analysis and specific substitution matrix, seed length etc.

REPLY:

We now added a table where different values for T are used, to see how this parameter affects the resulting trees. For some of the data shown in the manuscript, a different cut-off value might be indeed a small advantage. But in some cases, the program would work better with a larger cut-off value T, while in other cases, a lower value of T would improve the results. So it is not at all obvious, how optimal threshold values can be found automatically. A systematic study of this issue is beyond the scope of the present paper. And we think that for our benchmark program runs, our threshold of T=0 works well enough in terms of the resulting trees. The figure shows that, by and large, we can well distinguish between random and homologous spaced-word matches, even with our threshold T=0.

REVIEWER:
- P4L12: can we set a fixed random seed to ensure reproducibility?

REPLY:
Yes, see our reply to the last comment from Reviewer #4. We added all pattern sets that we used in the test runs to the "supplementary material". Also, we now have an option to use a user-defined pattern set, instead of generating pattern sets probabilistically with rasbhari. This way, one can exactly reproduce the results on our benchmark data, using the same pattern sets.

REVIEWER:
- experimental setup: please add test system configuration, program versions and command lines used

REPLY:
See above, we added the system configuration (first paragraph of "Results"). We added program versions where available, but not all programs that we tested have version numbers.

REVIEWER:
- it would be helpful to have a table summarizing dataset characteristrics (#taxa, genome/proteome size, reference tree used)

REPLY:
Done

REVIEWER:
- Figure 2: could it be that extremely poor distance estimates given by competing methods are - at least partially - due to a normalization artifact? The respective curves suggest that the distances reported by other methods might be on the logarithm scale. This would also explain why their branch length estimates in the empirical phylogentic trees are only moderately worse than those of ProtSPAM (Table 2).

REPLY:
This is a very interesting question. The question is if there is a possible "transformation" that would turn the usual word-frequency distances into estimates of "real" distances, i.e. substitutions per position. More generally, it would be interesting to know why word-frequency methods lead to reasonable tree topologies, even if these distances are far from the "real" distances. But this point is clearly not within the scope of our study, this would be a completely new project, not directly related to our present paper. (Similar questions are

discussed in Morgenstern et al., 2015).

REVIEWER:
- Table 1: please (additionally) report realtive Robinson-Foulds (RF) distances since those are much easier to interpret

REPLY:
Done

REVIEWER:
- Table 2: why are both "E. coli" and "Brassicea" datasets missing from this table?

REPLY:
Table 2 in the previous version of the manuscript reported the "branch score" distances to reference trees. This assumes that the reference trees have branch lengths. For E.coli and for the plant data sets, however, we had only topologies as reference, no branch lengths. Since the "branch scores" caused also other confusion, we decided to omit them completely (see our reply to the next reviewer comment).

REVIEWER:
- Table 2: could authors please describe how they normalized the branch lengths? even for the methods that use expected number of substituions per site as their branch length unit, using different data types (DNA/AA) and genomic regions (conserved genes vs. non-coding regions) will yield different estimates.

REPLY:
We did not normalize the branch lengths in the previously included table. Generally, the branch-score distances that we reported (Table 2 of the previous manuscript) caused some confusion: (a) since the other alignment-free methods that we evaluated (except for FSWM) do not attempt to estimate phylogenetic distances, it seems unfair to evaluate their distances under the "branch-score" metric, even if they are normalized. (b) For some of the reference trees, we only have their topologies not the branch lengths, so the branch-score distance is not applicable. For these reasons, all other papers in the field only use the RF distances. We think it is best to do the same and to omit the branch scores altogether.

REVIEWER:
- Table 3: are these single-core runtimes, or was multi-threading used for some/all of the programs?

REPLY:
We used multi-threading (40 threads) for those programs that were able to use multiple threads. This is now clarified in the main text and in the table legend.

REVIEWER:
- Table 3: on "Brassicea" and "813 projaryotes" datasets, ProtSPAM runs ~10-300x faster than FSWM. This is a bit surprising, given that two methods are highly similar. Could authors please provide an explaination for this remarkable difference?
Is it due to much smaller proteome vs. genome size? Due to fewer word matches? Or inefficient implementation of FSWM? Please clarify.

REPLY:
The main reason for the difference in speed is the difference between genome and proteome size for eukaryotes (see above). This is now discussed in the "Discussion".

REVIEWER:
- Figures 3 and 5: whenever possible, please use identical branch length scaling factors for easier interpretation

REPLY:
Some trees were taken from the literature, so here it was difficult to scale them exactly.

REVIEWER:
- P9L51, Figure 4 and 800+ prokaryotes tree: Color-coded clades correspond to bacterial phyla, that is, major groups that have split very early and have high sequence divergence. It is therefore not surprising that all methods were successfull in recovering these well-established clades. The difficult parts of this phylogeny are the relationships within the clades as well as the branching order of the phyla (deep splits).

REPLY
We agree. But given the size of the data set, this is not easy to visualize. We add a figure to the supplementary material.

REVIEWER:
- one interesting question that remains unanswered by this study is which input data type is better suited for alignment-free phylogenetic inference: whole-proteome, whole-genomes or whole-exome/-transcriptome? This appears to be relevant given that proteomes might be less readily available compared to genomes, as exemplified by the fact that the authors had to reduce some of the empirical datasets due to missing proteomes. On the other hand, direct proteome vs. exome comparison could deconvolve the effects of more conserved characters (AA vs. DNA) and more conserved regions (coding vs. non-coding).

REPLY:
In general, protein sequences are used for distantly related species, while DNA sequences are more suitable for closely related sequences. This was the whole motivation for our approach, as we mention in the "Introduction". It would be indeed very interesting to study this point in more detail, i.e. to find out more precisely in which situations whole-proteome sequence comparison is superior to whole-genome comparison with our spaced-words approach. But a systematic study of this question is beyond the scope of the present paper.

REVIEWER:
- would the authors please consider depositing all relevant supplementary files (empirical and simulated sequences, trees, scripts/command lines, results obtained by different programs etc.) in a public repository?

REPLY:
We uploaded this to http://projects.gobics.de/data/protspam/paperData.tgz We will add a public link to github.

REVIEWER:

- supplementary information provided for the review seems to be incomplete: e.g., I cannot find reference trees for most datasets, as well as simulated sequences for substitution rates > 1.0

REPLY
We added this.

==
==

ANONYMOUS REVIEWER WITH USEFUL COMMENTS (Reviewer #2):

REVIEWER:
1) It will be helpful if you provide a user friendly .exe of the program for biologists with no knowledge in programming.

REPLY:
We think that a user-friendly web-interface would be most useful for wet-lab biologists, so this has priority for us. Most experienced computational biologists are working under Linux/Unix anyway. But we will consider providing an .exe in the future.

REVIEWER:
2) Abstract: " Prot-SpaM can be used to calculate
high-quality phylogenetic trees from whole-proteome sequences in
a matter of seconds or minutes and often outperforms other alignmentfree
approaches." you should provide the size of the trees that are computed in seconds or minutes.

REPLY:
Done. It's now "dozens of whole proteins" in the abstract. More details can be found in in the main text.

REVIEWER:
3) I suggest to mention in the intro and discussion the fact that in bacteria there are frequent HGT events and thus the accurate evolutionary model is network and not a tree.

REPLY:
This is certainly true and could be mentioned. However, since our goal is to estimate distances without full sequence alignments, we prefer not to address this topic as it could distract from the main content the paper.

REVIEWER:
4) "Thus, for distal species, it may be hard to detect similarities
between genes at the DNA-sequence level, while homologies may be
still detectable among protein sequences." It is important to discuss that fact that whole genomes include additional information (e.g. regulatory signals) that does not exist in the proteome.

REPLY:
We fully agree. But here, we discuss the advantage/disadvantage of DNA/proteins

depending on the evolutionary distances. So we want to focus on this point.

REVIEWER:
5) The algorithm sound similar to previous papers including previous papers of the authors (e.g. refs 38,23, 65). You need to clearly explain the improvement/changes in the methodology. Specifically, what is the different between your " previous version of our program for nucleic-acid sequences" and the current one.

REPLY:
It's a completely new implementation for whole-proteome sequences. This involves technical differences due to the different alphabet size but, above all, because of different substitution matrices, parameter settings, program evaluation etc. So that's the improvement/changes compared to our previous paper. We think that this point is already addressed in the manuscript.

REVIEWER:
6) " To achieve this, we use the same greedy algorithm that we described in our previous paper [38]. " .." rasbhari uses a hill climbing algorithm to reduce the overlap complexity ". For a better readability, please provide more details related to the different part of the method (even if they are published in a different paper).

REPLY:
Done. We shortly outline the greedy algorithm.

REVIEWER:
7) "By default, our program uses a set of 5 patterns" why 5 ?

REPLY:
See our comments above. This was indeed missing in the previous manuscript; we discuss this point now in more detail and added test results with single patterns in addition to the 5-pattern sets.

REVIEWER:
8) Table 1: other methods are better in most cases. Is this true that methods such as ACS tend to outperform your method for very large datasets?

REPLY:
We don't think that other methods are "better in most cases". As mentioned above, there were mistakes in the previous manuscript for the RF distance on the "Wolbachia II" data set (should be between 18 and 20, but was 28 in the previous table). ACS, CVtree and kmacs still outperforms Prot-SpaM on the large prokaryotic data set, but not on the other full-proteome data sets. The selected proteins from Wolbachia I are a special case which we now discuss in more detail in the "Discussion".

REVIEWER:
9) Table 3: can you discuss the features that effect the running time of your method and other methods. There is no datasets where your method is faster than all other methods.

REPLY:

We now compiled the program with a different option and used multiple cores. Therefore, the runtime is now much better than what we reported in the previous version of the paper. The runtime can be further improved, if the "single-pattern" option is used instead of the 5 patterns that we use by default. This does not affect the quality much. We now discuss this point in the "Discussion".

REVIEWER:
10) Maybe you can improve the biological discussion related to the results phylogenic trees.

REPLY:
We have to say, this comment is a bit vague. We discuss the results on Wolbachia in some detail, and we added two paragraphs on the results on whole proteomes vs. selected proteins in the "Discussion". But the focus of this paper is a new software tool, not on phylogeny as such.

REVIEWER:
11) It is very hard to see anything in figures 3-5.

REPLY:
We agree. Therefore, we added a larger figure to the supplementary material.

Close
