# Supplementary material for: Prot-SpaM: fast alignment-free phylogeny reconstruction based on whole-proteome sequences
Source: Gigascience. 2018 Dec 7;8(3):giy148. doi: 10.1093/gigascience/giy148 (PMC6436989; doi:10.1093/gigascience/giy148)
Supplement: Response_to_Reviewer_Comments_Revision_1.pdf [file giy148_response_to_reviewer_comments_revision_1.pdf]

Author's Response To Reviewer Comments

Close

Reviewer #3:

REVIEWER:
-. In the manuscript, "In addition, we ran Prot-SpaM on the protein sequences encoded by the 24 marker genes from Lang et al". However, no discussion was found.

REPLY:
This paper is on a new alignment-free approach, not on the phylogeny of prokaryotes. Therefore, we restricted ourselves in this example to compare the phylogenies produced by various alignment-free programs to a published reference phylogeny.

REVIEWER:
-. In discussion section, authors claim that "Another advantage of our approach is that it can reliably distinguish between local homologies and random background similarities. It can, thus, be applied to complete or incomplete proteomes" in comparison with alignment-based methods. I don't understand this sentence and how alignment-based approaches are lacking this advantage.

REPLY:
As we said: for alignment-based approach, orthologous genes/proteins must be selected first. With our "filtering" approach, we automatically and rather reliably discard non-homologous spaced-word matches, so it is not necessary to select orthologous genes/proteins in a first step. We think that this is an advantage. But this is the "Discussion" section and, of course, one can have different opinions on this point.

REVIEWER:
-. In response to the comment, Authors noted that K=4 was used for FFP method for proteome datasets. First of all, authors should include this information like authors noted K=10 for Kmacs whenever FFP was compared with Prot-SpaM because K-mer length is a key to FFP method. However, this information is missing in tables and figures except for Figure 2. Second, Jun et al's paper (PANS, 2010; 107:133-138) has discussed about the optimal K-mer length for FFP method for whole proteome dataset which is something near K=10~K=13, which is very different from the one used in the manuscript for comparison. Basically, authors picked up K for FFP far from optimal and compared it with Prot-SpaM. I guess that this might result in large RF scores for FFP method.

REPLY:
In the manuscript, we clarify that all competing programs were run with their default parameters. This is common practice if software programs are evaluated. In addition, we explicitly mention that, for FFP, this means k=4. We now added this information to Tables 2 and 3 for clarity.

REVIEWER:
-. For plants, authors noted that a set of plant taxa and a reference tree from the reference [26] were used. Since there are many trees shown in the reference [26], authors should

describe which tree they used as the reference tree. Also, authors described they used only partial set of plant taxa because some plants don't have proteomes deposited into GenBank. So, authors should describe how they rebuilt a reference tree for the partial set to calculate RF scores since they cannot use the reference tree directly from the reference tree [26].

REPLY:
We clarified this in the manuscript (we used the tree B from Figure 3 from [26] and removed the three species for which we could not obtain the proteomes from GenBank).

REVIEWER:
-. In response to the comment, Authors noted that "Alignment-free approaches are certainly computationally more efficient than 16S rRNA-based approaches where one first has to identify these sequences and then construct an alignment". This is not correct. Unlike Prot-SpaM which uses only space-word matches to calculate (dis)similarity between whole proteomes, other alignment free methods mentioned in the manuscript concern all information encoded in whole proteomes instead of trying to look at matched proteins/regions so that they keep all features present in whole proteomes to measure (dis)similarity between whole proteomes which is time consuming.

REPLY:
Yes, other alignment-free methods use information from the entire sequences (e.g. all k-mers or common substrings). Prot-SpaM, by contrast, restricts itself to spaced-word matches with positive score that can assumed to represent homologies. We think that this is an advantage compared to other approaches. We discuss this point in the "Discussion" but, again, one may have different views on this point. Apart from this point, we do not understand how this is related with 16S rRNA. And we still think, that alignment-free approaches are faster than alignment-based approaches with 16S RNA.

REVIEWER:
-. In response to the comment, Authors said that "We do this by comparing phylogenetic trees to reference trees, and this is what all papers on alignment-free methods are doing to evaluate new tools.". Yes, people compare the outputs (here trees) to validate new tools. But, authors compared methods indirectly through the reference trees to validate their method which means that reference trees used are one of major factors in validation steps. However, authors used different genomic features for different datasets for reference trees. For example, Ecoli/Shigella dataset used 2,034 core genes, Wolbachia dataset used 252 core genes, whole proteome dataset used 24 core genes, Metazoa 1080 core genes for reference trees. Prot-SpaM uses only space-word matches which could be similar to core genes so that I speculate could make core genes-based reference trees more similar to the ones generated by Prot-SpaM. Anyway, I believe that people validate new tools for phylogeny construction in the following way: use 16S rRNA a universal marker for Prokaryotes, and compare trees built by new tools to trees of 16S rRNA for datasets of interest (which are reference trees) in terms of RF score as authors already did. I believe that alignment free methods considered in the manuscript all compared their trees to 16S rRNA trees for prokaryotes.

REPLY:
We agree with the reviewer: evaluating phylogeny methods by comparing their results to reference trees can be problematic, since the reference trees may not be the "correct" trees. This has to be taken into account if such benchmarking results are analyzed. As reference

trees, we used trees published by experts in the literature, based on carefully selected marker genes. While we cannot be 100% sure that these trees are correct, we think that these published trees are certainly of higher quality than trees based on 16S RNA only. So it is quite natural that different marker genes were used, this has been done by experts on the respective taxa.

Reviewer #4: The authors have addressed all my concerns and I find the updates to the paper satisfactory.

Reviewer #5: In the revised version of the manuscript, the authors addressed most of my criticisms, either by undertaking the respective changes or by providing a justification for not doing so.

I have only a few minor suggestions/remarks:

- P7L29: "All test runs were done on a 10 x Intel(R) Xeon(R) CPU E7-4850 with 2.00GHz with 4 cores each summing up
to 40 cores and 1000GB RAM." Does it mean that inter-node parallelization was used for some programs? How this was achieved (MPI?). Please clarify.

REPLY:
This is not a cluster, it is one single server, so no internode communication was necessary. We used OpenMP for parallelization of Prot-SpaM and FSWM.

REVIEWER:
- P8L53: "The other alignment-free methods led to phylogenies with RF distances to the reference
tree between 22 and 44" - those numbers differ from Table 2, please double-check.

REPLY:
We corrected this.

REVIEWER:
- Table 2: please also double-check numbers in this table, e.g RF and rRF values for "Wolbachia I, whole proteomes"/FSWM appear to be inconsistent

REPLY:
We corrected this.

REVIEWER:
- Table 3: please indicate which programs where run in multi-threading mode (40 threads vs. 1 threads would make quite a difference!)

REPLY:
done

REVIEWER:
- P19L33: "This is not surprising, given the fact that in eukaryotes only a small part of the genome is protein-coding

sequence, so the proteomes are substantially smaller than the corresponding genomes." - could you please give proteome vs. genome size for this dataset to support this statement?

REPLY:
done

- Table 4 and 5: it seems that careful choice of parameter values can lead to substantial accuracy gains (which might be even higher if "favorable" values for several parameters are combined). Thus I still think that automatic parameter tuning based on dataset properties (spamograms, evolutionary distances) could be a promising future work direction.

REPLY:
Yes, we fully agree. But as the reviewer says, this will be "future work".

Close
