# Supplementary material for: Prot-SpaM: fast alignment-free phylogeny reconstruction based on whole-proteome sequences
Source: Gigascience. 2018 Dec 7;8(3):giy148. doi: 10.1093/gigascience/giy148 (PMC6436989; doi:10.1093/gigascience/giy148)
Supplement: Response_to_Reviewer_Comments_Revision_2.pdf [file giy148_response_to_reviewer_comments_revision_2.pdf]

Author's Response To Reviewer Comments

Close

REVIEWER:
Authors included the following sentence: "In addition, we ran Prot-SpaM on the protein sequences encoded by the 24 marker genes from Lang et al". But, it seems like that this is all which authors described about this test. So, What I meant with this comment is that if you aren't going to discuss about results from this test, please exclude this sentence. Or, please include analysis results.

REPLY:
It is not clear to us why we should exclude this sentence. And we do discuss these results in the "Discussion" section.

REVIEWER:
-. In response to the comment, Authors noted that K=4 was used for FFP method for proteome datasets. First of all, authors should include this information like authors noted K=10 for Kmacs whenever FFP was compared with Prot-SpaM because K-mer length is a key to FFP method. However, this information is missing in tables and figures except for Figure 2.

REPLY:
This is not true. We mentioned the parameter k=4 repeatedly (Table 2, Table 3 and main text), this should be more than enough. Normally, one would simply run competing programs with their respective default parameters, without explicitly mentioning these values. So we really do not understand why the reviewer wants us to mention the parameter value k=4 more often than we already do.

REVIEWER:
Second, Jun et al's paper (PANS, 2010; 107:133-138) has discussed about the optimal K-mer length for FFP method for whole proteome dataset which is something near K=10~K=13, which is very different from the one used in the manuscript for comparison. Basically, authors picked up K for FFP far from optimal and compared it with Prot-SpaM. I guess that this might result in large RF scores for FFP method.

REPLY:
As we said, we used the default value provided by the user. If there are better parameter values, the developers of FFP should have specified them as default values, this is not our fault.

REVIEWER:
Authors responded to this comment as follows:
In the manuscript, we clarify that all competing programs were run with their default parameters. This is common practice if software programs are evaluated. In addition, we explicitly mention that, for FFP, this means k=4. We now added this information to Tables 2 and 3 for clarity.

I understand that authors run softwares with default parameters. However, authors are now informed that K=4 is not an optimal K-mer length for FFP for proteome comparison so that

comparisons were not done fairly. If authors inform in the manuscript that "the optimal K-mer length for FFP method for proteome comparison is 10~13" along with a reference Jun et al's paper (PANS, 2010; 107:133-138) to convey accurate information, I am fine with the current comparison results.

REPLY:
We now added a whole paragraph in the "Discussion" addressing this point. Here, we explicitly mention that FFP might produce better results if larger values of k are used. We have to say, that this is very unusual, it is not our job to find out or discuss optimal parameter values for competing software programs. This should be done by their developers. So we hope very much that the reviewer is now satisfied.

REVIEWER:
-. In response to the comment, Authors said that "We do this by comparing phylogenetic trees to reference trees, and this is what all papers on alignment-free methods are doing to evaluate new tools.". Yes, people compare the outputs (here trees) to validate new tools. But, authors compared methods indirectly through the reference trees to validate their method which means that reference trees used are one of major factors in validation steps. However, authors used different genomic features for different datasets for reference trees. For example, Ecoli/Shigella dataset used 2,034 core genes, Wolbachia dataset used 252 core genes, whole proteome dataset used 24 core genes, Metazoa 1080 core genes for reference trees. Prot-SpaM uses only space-word matches which could be similar to core genes so that I speculate could make core genes-based reference trees more similar to the ones generated by Prot-SpaM. Anyway, I believe that people validate new tools for phylogeny construction in the following way: use 16S rRNA a universal marker for Prokaryotes, and compare trees built by new tools to trees of 16S rRNA for datasets of interest (which are reference trees) in terms of RF score as authors already did. I believe that alignment free methods considered in the manuscript all compared their trees to 16S rRNA trees for prokaryotes.

Authors responded to the comment as follows:
As reference trees, we used trees published by experts in the literature, based on carefully selected marker genes. While we cannot be 100% sure that these trees are correct, we think that these published trees are certainly of higher quality than trees based on 16S RNA only.

It is generally understood that the more genes are included, the better resolution is obtained. So, I agree with authors that reference trees used in the manuscript are better than 16S RNA-based trees since all reference trees were built based on sets of genes, not just one gene. But, the point I made previously was that reference trees used in the manuscript could favor authors' method since authors' method and reference trees were all based on partial regions of proteomes unlike all other alignment-free methods included for comparison. That's is why I suggested a more object way for fair comparison. However, it seems like that authors don't want to take further steps. Then, I am fine with the current manuscript if authors describe which methods use whole proteomes and which methods use partial information of whole proteomes when compared.

REPLY:
We already explained how we chose the reference trees for our evaluation. This is common practice, everybody in the field uses reference trees provided by experts, and these trees are usually based on multiple marker genes, not just on 16S RNA. Also, we already added a

paragraph in the "Discussion" where we discussed the point with the "partial information" vs. "whole proteomes". So we don't think that anything is missing here.

Close
