# Supplementary material for: Prot-SpaM: fast alignment-free phylogeny reconstruction based on whole-proteome sequences
Source: Gigascience. 2018 Dec 7;8(3):giy148. doi: 10.1093/gigascience/giy148 (PMC6436989; doi:10.1093/gigascience/giy148)
Supplement: Reviewer_3_Report_(Original_Submission) -- Se-Ran Jun — 6/18/2018 Reviewed [file giy148_reviewer_3_report_(original_submission).pdf]

Reviewer Report

Title: **Prot-SpaM: Fast alignment-free phylogeny reconstruction based on whole-proteome sequences**

Version: **Original Submission** **Date:** 6/18/2018

Reviewer name: **Se-Ran Jun**

Reviewer Comments to Author:

The authors presented an alignment-free method named Prot-SpaM which could estimate phylogenetic distances between incomplete or complete proteomes, and compared Prot-SpaM with other alignment-free methods in terms of computational time and how similar to reference trees using simulated data, prokaryotic, and eukaryotic datasets.

Recommendation: Author should prepare a major revision for a second review.

Minor Comments:
-. References are not ordered.
-. On page 4, the cartoon for explaining of concept of spaced-word matches is not correct.
-. Figure 2 legend says ProtFSWM not Prot-SpaM
-. On Page 5, authors checked if spaced-words matches in the two compared sequences are one-to-one mapping. Please describe how one-to-one mapping from one-to-multiple or multiple-to-multiple was defined?
-. On page 5, authors used Kimura model to approximate PAM distance. Please put a reference for Kimura model. If there are parameters involved in Kimura model, please describe how to estimate those parameters.
-. Authors used BLOSUM62 when to distinguish homologous spaced-word matches with random spaced-word matches. But, authors approximated PAM distance between protein sequences. Please describe rational different substitution matrices used for different purposes?
-. For Table 1, Table 2, Table 3, Figure 2, and Figure 5, please describe which length of K-mer was used for FFP method.
-. One page 8, "One interpretation is that misleading signal stemming from recombination events between Wolbachia strains is less problematic for alignment-free analysis then a reduction in he dataset size." <= please revise the sentence.
-. On page 9, "we applied Prot-SpaM to all available protein sequences from these 813 taxa. In addition, we ran Prot-SpaM on the protein sequences encoded by the 24 marker genes from Lang et al." <= For application of Prot-SpaM to two different type of datasets, were same selected spaced-word matches and same patterns used?
-. For Table 3, what is the unit of computational time (seconds/minutes/hours)?

Major Comments:
-. Since authors describes a new alignment-free method for whole-proteome phylogeny, please include the existing alignment-free method developed specifically for whole proteome phylogeny ("Whole-proteome phylogeny of prokaryotes by feature frequency profiles: An alignment-free method with optimal feature resolution" by Jun et al, PANS 2010; 107:133-138") for the comparison.
-. No discussion found about Table 3 which summarized computational time for Prot-SpaM, and other alignment-free methods.
-. Authors claims that Prot-SpaM generates more statistically meaningful trees than other alignment-free methods. But, I don't see description of statistical confidence on internal nodes. Please describe how to impose statistical confidence on internal nodes of trees generated by Prot-SpaM.
-. On page 9, "There are some differences within the clades, though, that should be further investigated."

<= please at least provide information of RF and Branch score distance between four trees in Figure 4.
-. In Figure 3, Did Prot-SpaM segregate E.coli from Shigella? The paper "Insights from 20 years of bacteria genome sequencing" published in Funct Integr Genomics, 2015; 15:141-161 (https://www.ncbi.nlm.nih.gov/pmc/articles/PMC4361730/) showed a tree which segregated E.coli from Shigella clearly using the method described in "Whole-proteome phylogeny of prokaryotes by feature frequency profiles: An alignment-free method with optimal feature resolution" by Jun et al, PANS 2010; 107:133-138. Please discuss about which alignment-free methods including Jun et al's method have a capability of segregating E.coli from Shigella.
-. On page 8, authors described analysis results of Wolbachia strains without presenting a tree. Please provide the tree with annotations according to discussion to support the analysis results. In the subsection of Wolbachia, authors described how to generate an alignment-based tree with Wolbachia II dataset. But, there is no discussion found about comparison results with other alignment-free methods for Wolbachia II dataset.
-. Selected spaced-word matches and patterns are the most important factors for the method, Prot-SpaM. It seems that a length of l=46, a weight of w = 6, 40 don't-care positions and five patterns were used through the study. I don't see how authors reached these parameter values. Please describe the optimization procedure for these parameters, l, w, five patterns. For these optimized values, does selected spaced-word matches mean spaced-word matches with scores >0? Please describe meaning of 'selected' in "selected spaced-word matches". Second, please clarify whether selected spaced-word matches with a length of l=46, a weight of w = 6, 40 don't-care positions, and five patterns fixed were for any dataset (simulated, prokaryotic, eukaryotic proteomes, protein sequences)? Please provide sets of selected string-word matches and patterns in Supplementary. Third, please describe computational time for defining selected spaced-word matches and patterns. Fourth, Prot-SpaM uses only selected spaced-word matches which indicates fraction of proteomes are compared instead of whole proteomes when being built phylogenies. Please describe what fraction of proteomes on average by Prot-SpaM were being used for datasets discussed in the manuscript.
-. In comparing Table 1 (RF distance) with Table 2 (Branch score distance), since Prot-SpaM captures evolutionary distance between two sequences unlike other alignment-free methods, Prot-SpaM should perform better than other alignment-free methods against alignment-based reference trees by Brach score distance. But, for example, trees by CVTree method were closer to alignment-based reference trees than trees by Prot-SpaM with branch-score distance for some datasets even though CVtree doesn't capture evolutionary distance at all. Please discuss about this issue.
-. In the manuscript, validation step was solely dependent on alignment-based reference trees which sounds like that authors tried to develop an alignment-free method which produces trees most resemblance to alignment-based tree. For example, a reference tree of 813 prokaryotes was based on 24 marker genes and was found to be very similar to be 16S rRNA-based tree. Then, according to the validation procedure, Prot-SpaM tried to prove that the method produces a tree most resemblance to a 16S rRNA-based tree which does not require orthologous analysis and might not be computationally inferior to Prot-SpaM. Furthermore, even though Prot-SpaM pairwise distance captures evolutionary distance, the distances on the Prot-SpaM tree cannot be interpreted with substitution rates since distance-based methods are only applicable. To investigate other advantages of Prot-SpaM, the method needs to be examined over taxonomic classification in comparison with other alignment-free methods since taxonomic classification captures evolutionary information. Please examine a capability of taxonomic classification for the datasets discussed in the manuscript at least at the species level in comparison with other alignment-free methods.
-. The following error messages occurred when compiling the code downloaded from "https://github.com/jschellh/ProtSpaM" by 'make'. It seems that sysinfo.h is not provided.

```
mkdir -p obj
g++ -fopenmp -c -Wall -std=c++11 -I ./include main.cpp -o obj/main.o
In file included from ./include/speedsens.hpp:6:0,
from ./include/rasbcomp.hpp:9,
from ./include/rasbhari.hpp:8,
from ./include/rasbimp.hpp:4,
from main.cpp:26:
./include/sensmem.hpp:7:25: fatal error: sys/sysinfo.h: No such file or directory
```

```
#include "sys/sysinfo.h"
^
compilation terminated.
make: *** [obj/main.o] Error 1
```

Methods

Are the methods appropriate to the aims of the study, are they well described, and are necessary controls included? Choose an item.

Conclusions

Are the conclusions adequately supported by the data shown? Choose an item.

Reporting Standards

Does the manuscript adhere to the journal's guidelines on minimum standards of reporting? Choose an item.

Choose an item.

Statistics

Are you able to assess all statistics in the manuscript, including the appropriateness of statistical tests used? Choose an item.

Quality of Written English

Please indicate the quality of language in the manuscript: Choose an item.

Declaration of Competing Interests

Please complete a declaration of competing interests, considering the following questions:

- Have you in the past five years received reimbursements, fees, funding, or salary from an organisation that may in any way gain or lose financially from the publication of this manuscript, either now or in the future?
- Do you hold any stocks or shares in an organisation that may in any way gain or lose financially from the publication of this manuscript, either now or in the future?
- Do you hold or are you currently applying for any patents relating to the content of the manuscript?
- Have you received reimbursements, fees, funding, or salary from an organization that holds or has applied for patents relating to the content of the manuscript?
- Do you have any other financial competing interests?
- Do you have any non-financial competing interests in relation to this paper?

If you can answer no to all of the above, write 'I declare that I have no competing interests' below. If your reply is yes to any, please give details below.

I declare that I have no competing.

I agree to the open peer review policy of the journal. I understand that my name will be included on my report to the authors and, if the manuscript is accepted for publication, my named report including any attachments I upload will be posted on the website along with the authors' responses. I agree for my report to be made available under an Open Access Creative Commons CC-BY license (http://creativecommons.org/licenses/by/4.0/). I understand that any comments which I do not wish to be included in my named report can be included as confidential comments to the editors, which will not be published.

I agree to the open peer review policy of the journal

To further support our reviewers, we have joined with Publons, where you can gain additional credit to further highlight your hard work (see: https://publons.com/journal/530/gigascience). On publication of this paper, your review will be automatically added to Publons, you can then choose whether or not to claim your Publons credit. I understand this statement.

Yes Choose an item.
