# Supplementary material for: Prot-SpaM: fast alignment-free phylogeny reconstruction based on whole-proteome sequences
Source: Gigascience. 2018 Dec 7;8(3):giy148. doi: 10.1093/gigascience/giy148 (PMC6436989; doi:10.1093/gigascience/giy148)
Supplement: Reviewer_3_Report_revision_1 -- Se-Ran Jun — 9/17/2018 Reviewed [file giy148_reviewer_3_report_revision_1.pdf]

Reviewer Report

Title: Prot-SpaM: Fast alignment-free phylogeny reconstruction based on whole-proteome sequences

Version: Revision 1 Date: 9/17/2018

Reviewer name: Se-Ran Jun

Reviewer Comments to Author:

Methods

Recommendation: Author should prepare a major revision for a third review.

Thanks for the update of definition of spaced-word matches.

Major comments:

-. In the manuscript, "In addition, we ran Prot-SpaM on the protein sequences encoded by the 24 marker genes from Lang et al". However, no discussion was found.

-. In discussion section, authors claim that "Another advantage of our approach is that it can reliably distinguish between local homologies and random background similarities. It can, thus, be applied to complete or incomplete proteomes" in comparison with alignment-based methods. I don't understand this sentence and how alignment-based approaches are lacking this advantage.

-. In response to the comment, Authors noted that K=4 was used for FFP method for proteome datasets. First of all, authors should include this information like authors noted K=10 for Kmacs whenever FFP was compared with Prot-SpaM because K-mer length is a key to FFP method. However, this information is missing in tables and figures except for Figure 2. Second, Jun et al's paper (PANS, 2010; 107:133-138) has discussed about the optimal K-mer length for FFP method for whole proteome dataset which is something near K=10~K=13, which is very different from the one used in the manuscript for comparison. Basically, authors picked up K for FFP far from optimal and compared it with Prot-SpaM. I guess that this might result in large RF scores for FFP method.

-. For plants, authors noted that a set of plant taxa and a reference tree from the reference [26] were used. Since there are many trees shown in the reference [26], authors should describe which tree they used as the reference tree. Also, authors described they used only partial set of plant taxa because some plants don't have proteomes deposited into GenBank. So, authors should describe how they rebuilt a reference tree for the partial set to calculate RF scores since they cannot use the reference tree directly from the reference tree [26].

-. In response to the comment, Authors noted that "Alignment-free approaches are certainly computationally more efficient than 16S rRNA-based approaches where one first has to identify these sequences and then construct an alignment". This is not correct. Unlike Prot-SpaM which uses only space-word matches to calculate (dis)similarity between whole proteomes, other alignment free methods mentioned in the manuscript concern all information encoded in whole proteomes instead of trying to look at matched proteins/regions so that they keep all features present in whole proteomes to measure (dis)similarity between whole proteomes which is time consuming.

-. In response to the comment, Authors said that "We do this by comparing phylogenetic trees to reference trees, and this is what all papers on alignment-free methods are doing to evaluate new tools.". Yes, people compare the outputs (here trees) to validate new tools. But, authors compared methods indirectly through the reference trees to validate their method which means that reference trees used are one of major factors in validation steps. However, authors used different genomic features for different datasets for reference trees. For example, Ecoli/Shigella dataset used 2,034 core genes, Wolbachia dataset used 252 core genes, whole proteome dataset used 24 core genes, Metazoa 1080 core genes for reference trees. Prot-SpaM uses only space-word matches which could be similar to core genes so that I speculate could make core genes-based reference trees more similar to the ones generated by Prot-SpaM. Anyway, I believe that people validate new tools for phylogeny construction in the following way: use 16S rRNA a universal marker for Prokaryotes, and compare trees built by new tools to trees of 16S rRNA for datasets of interest (which are reference trees) in terms of RF score as authors already did. I believe that alignment free methods considered in the manuscript all compared their trees to 16S rRNA trees for prokaryotes.

Are the methods appropriate to the aims of the study, are they well described, and are necessary controls included? Choose an item.

Conclusions

Are the conclusions adequately supported by the data shown? Choose an item.

Reporting Standards

Does the manuscript adhere to the journal's guidelines on minimum standards of reporting? Choose an item.

Choose an item.

Statistics

Are you able to assess all statistics in the manuscript, including the appropriateness of statistical tests used? Choose an item.

Quality of Written English

Please indicate the quality of language in the manuscript: Choose an item.

Declaration of Competing Interests

Please complete a declaration of competing interests, considering the following questions:

- Have you in the past five years received reimbursements, fees, funding, or salary from an organisation that may in any way gain or lose financially from the publication of this manuscript, either now or in the future?
- Do you hold any stocks or shares in an organisation that may in any way gain or lose financially from the publication of this manuscript, either now or in the future?
- Do you hold or are you currently applying for any patents relating to the content of the manuscript?

- Have you received reimbursements, fees, funding, or salary from an organization that holds or has applied for patents relating to the content of the manuscript?
- Do you have any other financial competing interests?
- Do you have any non-financial competing interests in relation to this paper?

If you can answer no to all of the above, write 'I declare that I have no competing interests' below. If your reply is yes to any, please give details below.

I declare that I have no competing.

I agree to the open peer review policy of the journal. I understand that my name will be included on my report to the authors and, if the manuscript is accepted for publication, my named report including any attachments I upload will be posted on the website along with the authors' responses. I agree for my report to be made available under an Open Access Creative Commons CC-BY license (http://creativecommons.org/licenses/by/4.0/). I understand that any comments which I do not wish to be included in my named report can be included as confidential comments to the editors, which will not be published.

I agree to the open peer review policy of the journal

To further support our reviewers, we have joined with Publons, where you can gain additional credit to further highlight your hard work (see: https://publons.com/journal/530/gigascience). On publication of this paper, your review will be automatically added to Publons, you can then choose whether or not to claim your Publons credit. I understand this statement.

Yes Choose an item.
