# Supplementary material for: Prot-SpaM: fast alignment-free phylogeny reconstruction based on whole-proteome sequences
Source: Gigascience. 2018 Dec 7;8(3):giy148. doi: 10.1093/gigascience/giy148 (PMC6436989; doi:10.1093/gigascience/giy148)
Supplement: Reviewer_4_Report_(Original_Submission) -- Sriram Chockalingam — 6/20/2018 Reviewed [file giy148_reviewer_4_report_(original_submission).pdf]

Reviewer Report

Title: **Prot-SpaM: Fast alignment-free phylogeny reconstruction based on whole-proteome sequences**

Version: **Original Submission** **Date:** 6/20/2018

Reviewer name: **Sriram Chockalingam**

Reviewer Comments to Author:

The paper presents Prot-SpaM, a method to construct phylogenetic trees using proteome data based on the alignment-free spaced-word approach. The proposed method estimates pairwise distances for the set of sequences by computing the mapping of spaced words. The authors evaluate their approach using simulated data and also demonstrate the approach using for different real datasets, ranging from microbial data to plant proteomes. The authors also provide a rigorous method to select the threshold parameter using the spaced-word match histogram. Using the experiments using simulated data, they show Prot-SpaM proves a better approximation w.r.t to the expected distance computed by the Kimura model. The authors have made the software freely available at github, and is easy to build and run on a typical linux machine.

While the approach appears to be sound and reasonable, I have the following concerns which I would like to be addressed.

1. The key difficulty when using spaced-words approach in practice is the selection of the key parameters (a) pattern weight (b) number of don't care positions, (c) number of patterns and (d) minimum threshold. The paper includes a very good discussion on the selection of minimum threshold using spaced-word histogram. However, there is no guidance on how the other parameters can be selected. Even though all the experiments use the default parameters, a brief discussion on the effect of these parameters can be useful for practitioners.
2. The paper mentions that program uses 5 patterns, by default. Is there any reason for this default ?
3. The paper mentions that the pattern set is identified using a probabilistic algorithm, rasbhari, and hence, for two different runs with the same parameters can result in two different pattern sets. How does this affect the output trees ? In the experiments with real datasets, the authors provide a single RF distance in the Table 1. How does the RF distance varies w.r.t the probabilistic pattern set selection ?
4. In the descriptions of the algorithm (specifically in the third and sixth paragraphs of the introduction), the authors use the word 'alignment' to specify the spaced word pattern matches. I think this use of alignment description here is a bit confusing because pattern matching with spaced words is scored only in terms of the hamming distance.
5. For experiments with simulated data (Section 3.1), the authors mention that they evaluated 1000 sequence pairs for each distance data. They also mention that they ran with both default parameters and with only one pattern. Does the error bars (standard deviations) plotted in figure 2 include results from both runs or only the default runs ?
6. For the microbial phylogeny, the authors mention that the tree obtained with Prot-SpaM contains essentially the same clades. It is very difficult to see from Figure 3, in terms of the clades, how different the tree constructed by kmacs or ACS from reference tree, which has lower RF distances compared to Prot-SpaM ?
7. While reporting the runtime, it is instructive to provide the type of machine the software were run. Also, it seems the implementation available at https://github.com/jschellh/ProtSpaM has multi-threaded capability, but the table 3 doesn't mention if it was run using single thread or multiple threads.
8. While the paper says that the use 5 patterns with w = 6 as default, the software in github appears to use only one pattern with w = 8 for default. Is there a reason for this discrepancy ?

I also reviewed the supplementary data and the documentation seems sufficient to reproduce the results.

Since there is probabilistic algorithm to select of the pattern sets, it would be useful to have a few seed patterns for the simulated data, if possible. Also, it can be useful for future research if the authors can provide all the reference trees along with the supplementary data.

Methods

Are the methods appropriate to the aims of the study, are they well described, and are necessary controls included? Choose an item.

Conclusions

Are the conclusions adequately supported by the data shown? Choose an item.

Reporting Standards

Does the manuscript adhere to the journal's guidelines on minimum standards of reporting? Choose an item.

Choose an item.

Statistics

Are you able to assess all statistics in the manuscript, including the appropriateness of statistical tests used? Choose an item.

Quality of Written English

Please indicate the quality of language in the manuscript: Choose an item.

Declaration of Competing Interests

Please complete a declaration of competing interests, considering the following questions:

- Have you in the past five years received reimbursements, fees, funding, or salary from an organisation that may in any way gain or lose financially from the publication of this manuscript, either now or in the future?
- Do you hold any stocks or shares in an organisation that may in any way gain or lose financially from the publication of this manuscript, either now or in the future?
- Do you hold or are you currently applying for any patents relating to the content of the manuscript?
- Have you received reimbursements, fees, funding, or salary from an organization that holds or has applied for patents relating to the content of the manuscript?
- Do you have any other financial competing interests?
- Do you have any non-financial competing interests in relation to this paper?

If you can answer no to all of the above, write 'I declare that I have no competing interests' below. If your reply is yes to any, please give details below.

I declare that I have no competing.

I agree to the open peer review policy of the journal. I understand that my name will be included on my report to the authors and, if the manuscript is accepted for publication, my named report including any attachments I upload will be posted on the website along with the authors' responses. I agree for my report to be made available under an Open Access Creative Commons CC-BY license (http://creativecommons.org/licenses/by/4.0/). I understand that any comments which I do not wish to be included in my named report can be included as confidential comments to the editors, which will not be published.

I agree to the open peer review policy of the journal

To further support our reviewers, we have joined with Publons, where you can gain additional credit to further highlight your hard work (see: https://publons.com/journal/530/gigascience). On publication of this paper, your review will be automatically added to Publons, you can then choose whether or not to claim your Publons credit. I understand this statement.

Yes Choose an item.
