# Supplementary material for: Prot-SpaM: fast alignment-free phylogeny reconstruction based on whole-proteome sequences
Source: Gigascience. 2018 Dec 7;8(3):giy148. doi: 10.1093/gigascience/giy148 (PMC6436989; doi:10.1093/gigascience/giy148)
Supplement: Reviewer_5_Report_(Original_Submission) -- Alexey Kozlov — 6/21/2018 Reviewed [file giy148_reviewer_5_report_(original_submission).pdf]

Reviewer Report

Title: Prot-SpaM: Fast alignment-free phylogeny reconstruction based on whole-proteome sequences

Version: Original Submission **Date:** 6/21/2018

Reviewer name: Alexey Kozlov

Reviewer Comments to Author:

Genenal comments:

In this manuscript, Leimeister et al. present a novel tool for alignment-free phylogeny reconstruction from protein sequences.
Overall, the manuscript is well structured, written in clear language and is easy to follow. Tables and plots are used adequately to represent experimental results (see some specific suggestions below).

The presented method appears to be a rather minor modification of the existing FSWM tool (Leimeister et al. 2017, Bioinformatics). In particular, the only differnces seem to be: (1) using a different input alphabet (aminoacids) and thus a different scoring matrix (BLOSUM62), (2) using multiple patterns, and (3) using Kimura distance correction. Given that optimizations (2) and (3) can also be applied for genomic/DNA sequences, is it really justified to implement these minor modifications as a separate software tool? Arguably, having a single tool which can handle both DNA and protein sequences would be more convenient for the users while also simplifying software maintenance.

Despite limited methodological novelty, ProtSPAM could be still a valuable addition to the field if it can achieve significant improvements over existing methods in terms of the tree reconstruction accuracy and/or speed. Unfortunately, presented experimental results cannot convincingly demonstrate this. When compared to FSWM, ProtSPAM is significantly more accurate only on 1 dataset out of 6 (Tables 1 and 2). And even on this dataset (813 prokaryotes), ProtSPAM showed higher topological error (RF distance) than 3 competing methods (CVTree, kmacs and ACS). However, even the best method (kmacs) inferred a tree which is very dissimilar to the reference (relative RF distance rRF=0.54). Therefore, differences between e.g., kmacs (rRF=0.54), ProtSPAM (rRF=0.63) and FSWM (rRF=0.83) are remarkable but less important, since all methods can be considered very inaccurate. Moreover, these results can be confounded by the instability of the reference tree itself, which is quite common for large trees inferred from a limited set of genes (see Figure 6 in Lang et al. 2013). Additionaly, it is not clear whether we can expect high level of similarity between trees built from whole proteomes and ribosome proteins, since the latter one is in turn quite different from the 16S tree (rRF=~0.5, same figure).

Given the above inherent problems with empirical datasets, I suggest to complement ProtSPAM evaluation with benchmarks on simulated data. In addition to the known reference tree, simulation allows to freely variate number of taxa, genome/proteome size and substitution rates, and thereby to compare performance of alternative methods more systematically. I can also recommend (Zhou 2017, https://academic.oup.com/mbe/article/35/2/486/4644721#113627412) for a decent set of thoroughly analyzed empirical phylogenomic datasets.

In terms of computational speed, ProtSPAM seems to drastically outperform FSWM and has runtimes comparable to other methods which operate on protein sequences. Although >100x runtime difference between two highly similar methods looks somewhat surprising (see below), if this speedup is real and consistent, it could be a major argument in favor of ProtSPAM.

In summary, the authors should try to make it clear under which conditions ProtSPAM outperforms other

methods (high sequence divergence? large trees? large genomes?), and support this claim by convincing experimental results.

Specific comments:

METHODS

- Figure 1: In my opinion, plots for empirical datasets (C and D) do not quite support authors' choise of the alignment score cutoff (T=0). Furthermore, the optimal cutoff value will probably depend on k-mer length/weight, as well as on the substitution matrix selected. It appears more reasonable to use an adaptive cutoff T, derived from the spamogram for the particular dataset under analysis and specific substitution matrix, seed length etc.

- P4L12: can we set a fixed random seed to ensure reproducibility?

RESULTS

- experimental setup: please add test system configuration, program versions and command lines used

- it would be helpful to have a table summarizing dataset characteristrics (#taxa, genome/proteome size, reference tree used)

- Figure 2: could it be that extremely poor distance estimates given by competing methods are - at least partially - due to a normalization artifact? The respective curves suggest that the distances reported by other methods might be on the logarithm scale. This would also explain why their branch length estimates in the empirical phylogentic trees are only moderately worse than those of ProtSPAM (Table 2).

- Table 1: please (additionally) report realtive Robinson-Foulds (RF) distances since those are much easier to interpret

- Table 2: why are both "E. coli" and "Brassicea" datasets missing from this table?

- Table 2: could authors please describe how they normalized the branch lengths? even for the methods that use expected number of substituions per site as their branch length unit, using different data types (DNA/AA) and genomic regions (conserved genes vs. non-coding regions) will yield different estimates.

- Table 3: are these single-core runtimes, or was multi-threading used for some/all of the programs?

- Table 3: on "Brassicea" and "813 projaryotes" datasets, ProtSPAM runs ~10-300x faster than FSWM. This is a bit surprising, given that two methods are highly similar. Could authors please provide an explaination for this remarkable difference?
Is it due to much smaller proteome vs. genome size? Due to fewer word matches? Or inefficient implementation of FSWM? Please clarify.

- Figures 3 and 5: whenever possible, please use identical branch length scaling factors for easier interpretation

- P9L51, Figure 4 and 800+ prokaryotes tree: Color-coded clades correspond to bacterial phyla, that is, major groups that have split very early and have high sequence divergence. It is therefore not surprising that all methods were successfull in recovering these well-established clades. The difficult parts of this phylogeny are the relationships within the clades as well as the branching order of the phyla (deep splits).

DISCUSSION

- one interesting question that remains unanswered by this study is which input data type is better suited for alignment-free phylogenetic inference: whole-proteome, whole-genomes or whole-exome/-transcriptome? This appears to be relevant given that proteomes might be less readily available compared to genomes, as exemplified by the fact that the authors had to reduce some of the empirical datasets due to missing proteomes. On the other hand, direct proteome vs. exome comparison could deconvolve the effects of more conserved characters (AA vs. DNA) and more conserved regions (coding vs. non-coding).

DATA AVAILABILITY:

- would the authors please consider depositing all relevant supplementary files (empirical and simulated sequences, trees, scripts/command lines, results obtained by different programs etc.) in a public repository?

- supplementary information provided for the review seems to be incomplete: e.g., I cannot find reference trees for most datasets, as well as simulated sequences for substitution rates > 1.0

Methods

Are the methods appropriate to the aims of the study, are they well described, and are necessary controls included? Choose an item.

Conclusions

Are the conclusions adequately supported by the data shown? Choose an item.

Reporting Standards

Does the manuscript adhere to the journal's guidelines on minimum standards of reporting? Choose an item.

Choose an item.

Statistics

Are you able to assess all statistics in the manuscript, including the appropriateness of statistical tests used? Choose an item.

Quality of Written English

Please indicate the quality of language in the manuscript: Choose an item.

Declaration of Competing Interests

Please complete a declaration of competing interests, considering the following questions:

- Have you in the past five years received reimbursements, fees, funding, or salary from an organisation that may in any way gain or lose financially from the publication of this manuscript, either now or in the future?
- Do you hold any stocks or shares in an organisation that may in any way gain or lose financially from the publication of this manuscript, either now or in the future?

- Do you hold or are you currently applying for any patents relating to the content of the manuscript?
- Have you received reimbursements, fees, funding, or salary from an organization that holds or has applied for patents relating to the content of the manuscript?
- Do you have any other financial competing interests?
- Do you have any non-financial competing interests in relation to this paper?

If you can answer no to all of the above, write 'I declare that I have no competing interests' below. If your reply is yes to any, please give details below.

I declare that I have no competing.

I agree to the open peer review policy of the journal. I understand that my name will be included on my report to the authors and, if the manuscript is accepted for publication, my named report including any attachments I upload will be posted on the website along with the authors' responses. I agree for my report to be made available under an Open Access Creative Commons CC-BY license (http://creativecommons.org/licenses/by/4.0/). I understand that any comments which I do not wish to be included in my named report can be included as confidential comments to the editors, which will not be published.

I agree to the open peer review policy of the journal

To further support our reviewers, we have joined with Publons, where you can gain additional credit to further highlight your hard work (see: https://publons.com/journal/530/gigascience). On publication of this paper, your review will be automatically added to Publons, you can then choose whether or not to claim your Publons credit. I understand this statement.

Yes Choose an item.
