# Supplementary material for: Prot-SpaM: fast alignment-free phylogeny reconstruction based on whole-proteome sequences
Source: Gigascience. 2018 Dec 7;8(3):giy148. doi: 10.1093/gigascience/giy148 (PMC6436989; doi:10.1093/gigascience/giy148)
Supplement: Reviewer_5_Report_Revision_1 -- Alexey Kozlov — 9/18/2018 Reviewed [file giy148_reviewer_5_report_revision_1.pdf]

Reviewer Report

Title: **Prot-SpaM: Fast alignment-free phylogeny reconstruction based on whole-proteome sequences**

Version: **Revision 1** **Date:** 9/18/2018

Reviewer name: **Alexey Kozlov**

Reviewer Comments to Author:

In the revised version of the manuscript, the authors addressed most of my criticisms, either by undertaking the respective changes or by providing a justification for not doing so.

I have only a few minor suggestions/remarks:

- P7L29: "All test runs were done on a 10 x Intel(R) Xeon(R) CPU E7-4850 with 2.00GHz with 4 cores each summing up
to 40 cores and 1000GB RAM." Does it mean that inter-node parallelization was used for some programs? How this was achieved (MPI?). Please clarify.

- P8L53: "The other alignment-free methods led to phylogenies with RF distances to the reference tree between 22 and 44" - those numbers differ from Table 2, please double-check.

- Table 2: please also double-check numbers in this table, e.g RF and rRF values for "Wolbachia I, whole proteomes"/FSWM appear to be inconsistent

- Table 3: please indicate which programs where run in multi-threading mode (40 threads vs. 1 threads would make quite a difference!)

- P19L33: "This is not surprising, given the fact that in eukaryotes only a small part of the genome is protein-coding
sequence, so the proteomes are substantially smaller than the corresponding genomes." - could you please give proteome vs. genome size for this dataset to support this statement?

- Table 4 and 5: it seems that careful choice of parameter values can lead to substantial accuracy gains (which might be even higher if "favorable" values for several parameters are combined). Thus I still think that automatic parameter tuning based on dataset properties (spamograms, evolutionary distances) could be a promising future work direction.

Methods

Are the methods appropriate to the aims of the study, are they well described, and are necessary controls included? Choose an item.

Conclusions

Are the conclusions adequately supported by the data shown? Choose an item.

Reporting Standards

Does the manuscript adhere to the journal's guidelines on minimum standards of reporting? Choose an item.

Choose an item.

Statistics

Are you able to assess all statistics in the manuscript, including the appropriateness of statistical tests used? Choose an item.

Quality of Written English

Please indicate the quality of language in the manuscript: Choose an item.

Declaration of Competing Interests

Please complete a declaration of competing interests, considering the following questions:

- Have you in the past five years received reimbursements, fees, funding, or salary from an organisation that may in any way gain or lose financially from the publication of this manuscript, either now or in the future?
- Do you hold any stocks or shares in an organisation that may in any way gain or lose financially from the publication of this manuscript, either now or in the future?
- Do you hold or are you currently applying for any patents relating to the content of the manuscript?
- Have you received reimbursements, fees, funding, or salary from an organization that holds or has applied for patents relating to the content of the manuscript?
- Do you have any other financial competing interests?
- Do you have any non-financial competing interests in relation to this paper?

If you can answer no to all of the above, write 'I declare that I have no competing interests' below. If your reply is yes to any, please give details below.

I declare that I have no competing.

I agree to the open peer review policy of the journal. I understand that my name will be included on my report to the authors and, if the manuscript is accepted for publication, my named report including any attachments I upload will be posted on the website along with the authors' responses. I agree for my report to be made available under an Open Access Creative Commons CC-BY license (http://creativecommons.org/licenses/by/4.0/). I understand that any comments which I do not wish to be included in my named report can be included as confidential comments to the editors, which will not be published.

I agree to the open peer review policy of the journal

To further support our reviewers, we have joined with Publons, where you can gain additional credit to further highlight your hard work (see: https://publons.com/journal/530/gigascience). On publication of this paper, your review will be automatically added to Publons, you can then choose whether or not to claim your Publons credit. I understand this statement.

Yes Choose an item.
